# XRD and spectral dataset of the UV-A stable nanotubes of 3,5-bis(trifluoromethyl)benzylamine derivative of tyrosine

**DOI:** 10.1016/j.dib.2017.08.001

**Published:** 2017-08-15

**Authors:** R. Govindhan, B. Karthikeyan

**Affiliations:** Department of Chemistry, Annamalai University, Annamalainagar 608002, Tamilnadu, India

**Keywords:** Single amino acid, Photo-degradation, BTTPNTs, XRD, UV–vis, data

## Abstract

The data presented in this article are related to the research entitled of UV-A stable nanotubes. The nanotubes have been prepared from 3,5-bis(trifluoromethyl)benzylamine derivative of tyrosine (BTTP). XRD data reveals the size of the nanotubes. As-synthesized nanotubes (BTTPNTs) are characterized by UV–vis optical absorption studies [1] and photo physical degradation kinetics. The resulted dataset is made available to enable critical or extended analyzes of the BTTPNTs as an excellent light resistive materials.

**Specifications Table**

**Table:** 

**Subject area**	Chemistry
**More specific subject area**	Biomaterials and bio nanotechnology
**Type of data**	Tables, figures and schemes.
**How data was acquired**	Analysis of self-assembled single amino acid derived nanotubes having promising UV-A light stability.
**Data format**	Raw data
**Experimental factors**	UV-visible (UV–vis) spectroscopy measurements of coated quartz glass slides and XRD.
**Experimental features**	The UV spectral data and photo degradation results are related with UV-stability.
**Data source location**	XRD,UV –Vis absorbance and Photo reactors of Department of Chemistry, Annamalai University, India.
**Data accessibility**	Data included in this article. and accessible

**Value of the data**

• Data of this article is a collection of all XRD, UV–vis spectroscopic analysis and photo degradation of the synthesized nanotubes.
• The empirical graphs and plots provide a novel way to look at the application of the transmittance and absorption frequencies
• Light resistive property of the nanotubes is presumed from the data
• Raw Data tabulated will give an idea for the researchers to develop certain UV stable nanomaterials.

## 1 Data

**Fig. 1:**
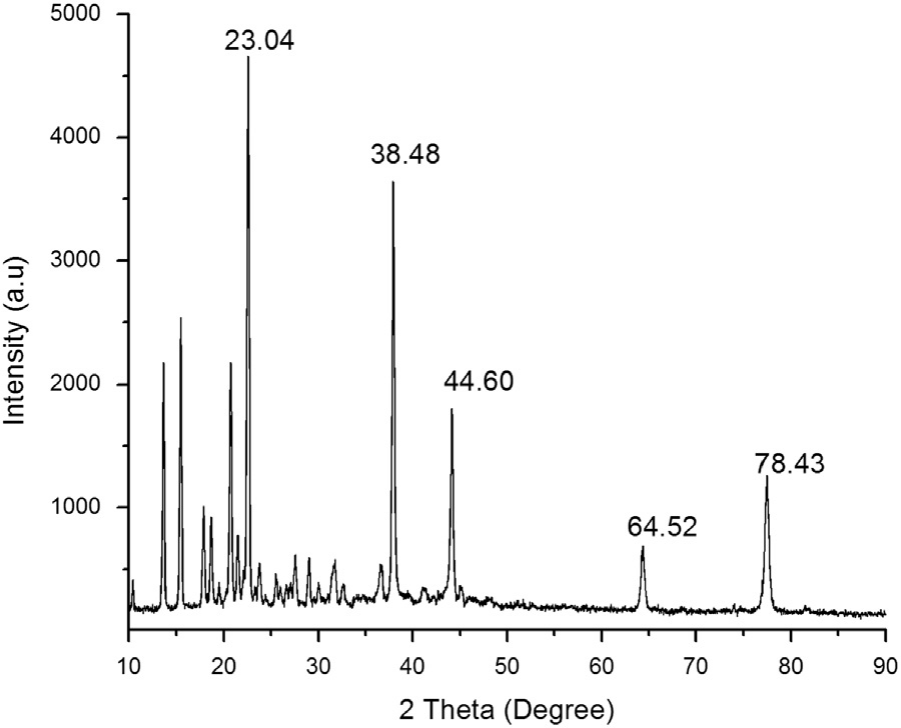
XRD pattern of BTTPNTs.

**Fig. 2:**
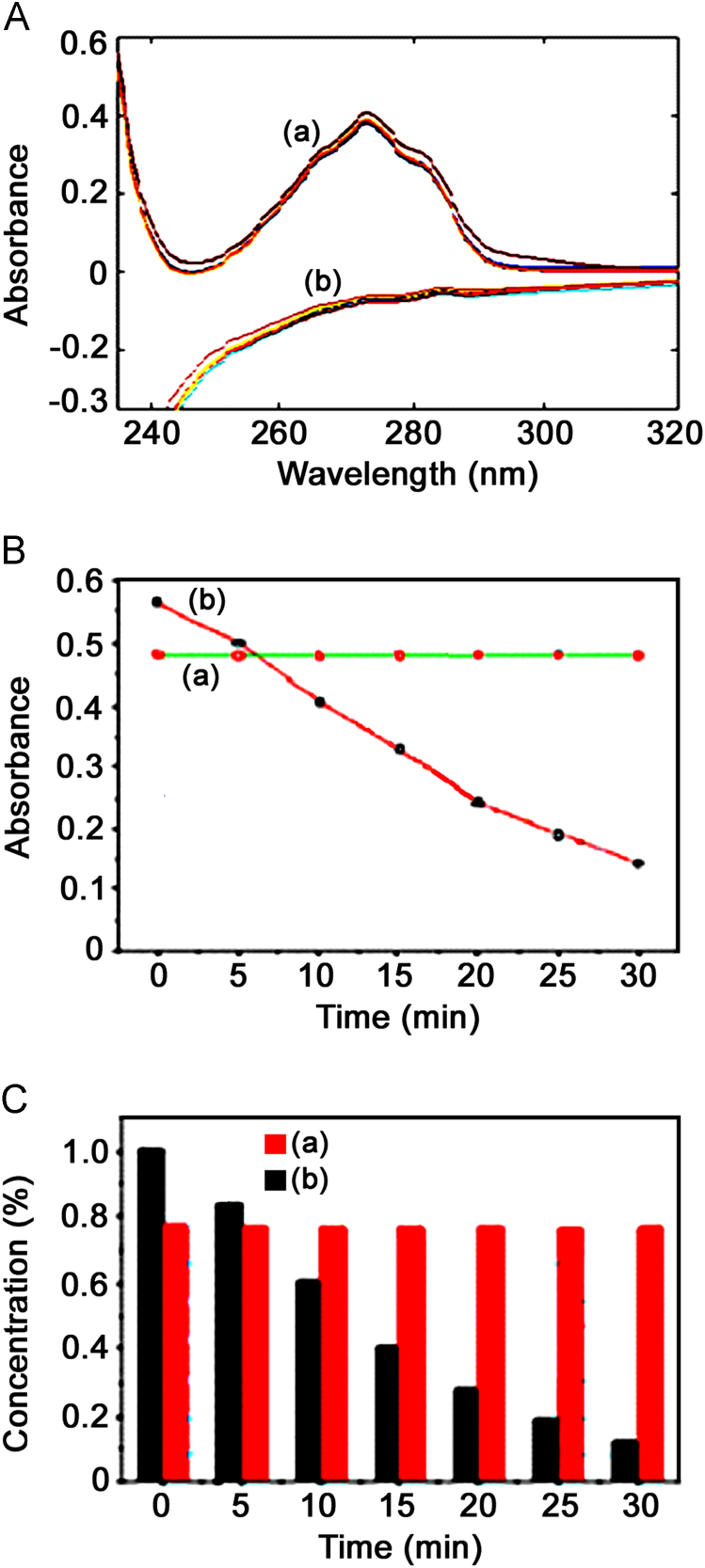
(A) Absorption spectrum of BTTPNTs and BTTP (UV-A) irradiated at 30 min time scale, (B) the absorbance plot between Abs Vs time (min) and (C) the bar charts represents to the change in concentration with respect to time (min).

**Scheme 1:**
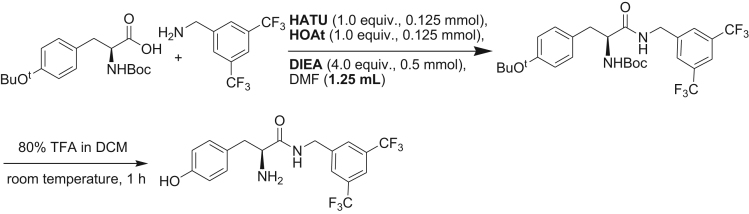
Schematic illustration for the solid phase synthesis of 3,5-bis(trifluoromethyl)benzylamine derivative of tyrosine (BTTP).

**Scheme 2:**
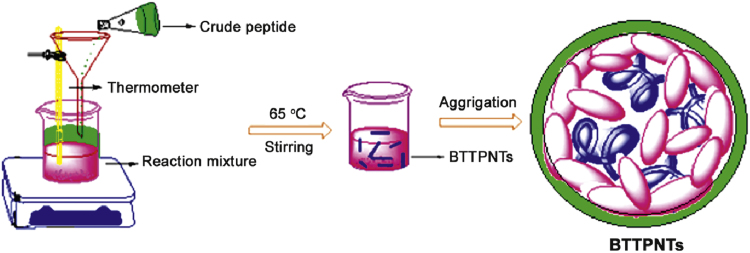
Synthesis of BTTPNTs and nanovesicles.

The dataset of this article provides information on the UV stable BTTPNTs. Scheme 1, Scheme 2 show the synthesis scheme and Fig. 1, Fig. 2 shows XRD and spectra. Kinetic graphs. Resulted data are provided in Supplementary Tables 1, 2 and 3.

## 2 Materials and methods

### 2.1 Materials

3,5-Bis(trifluoromethyl)benzylamine, 98.0%, Boc-AA-OH (AA = Tyr, 1.0 equiv, 0.125 mmol, 42 mg for Tyr), HATU (1.0 equiv., 0.125 mmol, 47.5 mg), HOAc (1.0 equiv., 0.125 mmol, 17 mg) and DIEA (4.0 equiv., 0.500 mmol, 0.087 mL) in dry DMF (1.25 mL) were bought from AAPPTEC, USA. KHSO_4,_ 99.0%, DCM, 98.0%, TFA, 98.0%, and other reagents and solvents were purchased from HiMedia Laboratories Pvt. Ltd., (Mumbai, India) and were used without further purification. All aqueous solutions were prepared with nanopure water. All apparatus and glassware's are washed with acetone, rinsed with deionized water (DIW) and dried with air hot owen at 100 °C, then used throughout the studies.

### 2.2 Synthesis of 3,5-bis(trifluoromethyl)benzylamine derivatives of tyrosine

In 3,5-bis(trifluoromethyl)benzylamine (1.0 equiv., 0.125 mmol, 30.5 mg) was added to a solution of Boc-AA-OH (AA = Tyr, 1.0 equiv, 0.125 mmol, 42 mg for Tyr), HATU (1.0 equiv., 0.125 mmol, 47.5 mg), HOAc (1.0 equiv., 0.125 mmol, 17 mg) and DIEA (4.0 equiv., 0.500 mmol, 0.087 mL) in dry DMF (1.25 mL) at room temperature. The reaction mixture was stirred for overnight (20 h) and then quenched with 0.5 M aqueous solution of KHSO_4_ (5 mL). It was then extracted with DCM (3×15 mL). The combined organic part was subsequently washed with brine, saturated sodium bicarbonate and again brine. Evaporation of DCM under reduced pressure using rotary evaporator gave the crude product in quantitative yield. The Boc group was removed by treating the product with 80% TFA in DCM for 1 h at room temperature. Removal of volatiles provided the unprotected 3,5-bis(trifluoromethyl)benzylamine derivative of the respective single amino acid. Washing with 1:4 solutions of ether and hexanes (3×10 mL) provided the final product in >80% yield with <95% (HPLC) (Scheme 1).

### 2.3 Synthesis of self-assembled nanotubes

The procedure was adopted from a reported method [2] as follows (Scheme 2). About 10 mg of aqueous solution (in nanopure water) of single amino acid derivatives was prepared in a closed Erlenmeyer flask with appropriate concentration. The flask containing desired solution and a magnetic stir bar was placed in pre warmed silicon oil bath (65 °C) and moderate stirring was continued for 30 min at that temperature. Heating was then stopped and the solution was brought to room temperature with gentle stirring for over a period of 3 h.

### 2.4 Characterization techniques

UV–vis (ultraviolet and visible light) absorbance spectra were recorded over a range of 800–200 nm with a Shimadzu UV-1650 PC spectrophotometer, operated at a resolution of 0.5 nm. The samples were filled in a quartz cuvette of 1 cm light-path length, and the absorption spectra were recorded with reference to deionized water and distilled ethanol.

Artificial UV-A environmental situation is created by photo reactor. UV-A absorption wavelength range was fixed at 365 nm. The concentration of 2×10^–4^ M FPNTs solution was used for the study. UV–vis (ultraviolet and visible light) absorbance spectra were recorded over a range of 800–200 nm with a Shimadzu UV-1650 PC spectrophotometer, operated at a resolution of 0.5 nm (see supporting information Tables 1–3).

## Conflict of interest

The authors declare no competing financial interest.

## Footnotes

Appendix A Supplementary data associated with this article can be found in the online version at http://dx.doi.org/10.1016/j.dib.2017.08.001.

## References

[1] Karthikeyan B., Giri A.K., Hruby V.J. (2014). J. Sol.-Gel Sci. Technol..

[2] Song Y., Challa S.R., Medforth C.J., Qiu Y., Watt R.K., Pena D., Miller J.E., Swol F.V., Shelnutt J.A. (2004). Chem. Commun..

