# Supplementary material for: XRD and spectral dataset of the UV-A stable nanotubes of 3,5-bis(trifluoromethyl)benzylamine derivative of tyrosine
Source: Data Brief. 2017 Aug 15;14:579–83. doi: 10.1016/j.dib.2017.08.001 (PMC5570576; doi:10.1016/j.dib.2017.08.001)
Supplement: Supplementary file 2 — Supplementary material [file mmc2.docx]

**Supplementary Material**

**Dataset on the UV-A stable 3,5-bis(trifluoromethyl)benzylamine derivative of tyrosine peptide nanotubes**

R. Govindhan and **B. Karthikeyan***

Department of Chemistry, Annamalai University, Annamalainagar 608 002, Tamilnadu, India.

**Table S1.** XRD data of BTTPNTs.

| **2 Theta (Degree)** | **Intensity (a.u)** | **FWHM** |
| --- | --- | --- |
| 5.98 | 1.4 | 0.1217 |
| 6.11 | 1.3 | 0.1217 |
| 6.36 | 1.2 | 0.1217 |
| 6.63 | 1 | 0.1217 |
| 6.83 | 1 | 0.1217 |
| 7.02 | 0.9 | 0.1217 |
| 7.16 | 0.9 | 0.1217 |
| 7.54 | 0.7 | 0.1217 |
| 7.85 | 0.7 | 0.1217 |
| 8.03 | 0.7 | 0.1217 |
| 8.41 | 0.6 | 0.1217 |
| 9.09 | 2.8 | 0.2218 |
| 9.37 | 0.4 | 0.1599 |
| 9.49 | 0.3 | 0.1344 |
| 9.73 | 0.6 | 0.1403 |
| 10.03 | 0.4 | 0.2738 |
| 10.42 | 3.4 | 0.1711 |
| 10.8 | 0.3 | 0.1523 |
| 11.04 | 0.1 | 0.008 |
| 13.69 | 30.7 | 0.173 |
| 14.02 | 0 | 0.0008 |
| 14.68 | 14.68 | 0 |
| 14.99 | 14.99 | 0 |
| 15.51 | 39.3 | 0.1824 |
| 15.82 | 0 | 0.0008 |
| 16.1 | 0.2 | 0.1217 |
| 16.59 | 16.59 | 0 |
| 17.02 | 17.02 | 0 |
| 17.93 | 14.8 | 0.2082 |
| 18.51 | 18.51 | 0 |
| 18.66 | 13.5 | 0.213 |
| 19.06 | 0 | 0.0262 |
| 19.48 | 2.1 | 0.148 |
| 19.93 | 19.93 | 0 |
| 20.11 | 20.11 | 0 |
| 20.15 | 20.15 | 0 |
| 20.28 | 0 | 0.0351 |
| 20.72 | 38.1 | 0.2185 |
| 21.49 | 10.6 | 0.2172 |
| 22.11 | 0 | 0.1217 |
| 22.58 | 94.9 | 0.2306 |
| 23.31 | 1.4 | 0.1854 |
| 23.77 | 7.3 | 0.2569 |
| 24.43 | 1.4 | 0.2421 |
| 25.01 | 0.1 | 0.1493 |
| 25.12 | 0 | 0.1916 |
| 25.54 | 5.9 | 0.2669 |
| 25.99 | 2.7 | 0.2271 |
| 26.06 | 26.06 | 0 |
| 26.61 | 3 | 0.2421 |
| 27.07 | 3.1 | 0.2577 |
| 27.57 | 8.5 | 0.2537 |
| 28.03 | 28.03 | 0 |
| 28.2 | 28.2 | 0 |
| 28.22 | 28.22 | 0 |
| 28.23 | 28.23 | 0 |
| 28.36 | 28.36 | 0 |
| 28.39 | 28.39 | 0 |
| 29.01 | 7.7 | 0.2413 |
| 29.54 | 0.2 | 0.0959 |
| 30.05 | 2.6 | 0.2171 |
| 30.43 | 0.1 | 0.029 |
| 30.55 | 30.55 | 0 |
| 30.89 | 30.89 | 0 |
| 31.01 | 0.4 | 0.1436 |
| 31.75 | 7.7 | 0.3287 |
| 32.26 | 0.1 | 0.1893 |
| 32.65 | 3.5 | 0.2912 |
| 32.95 | 0.3 | 0.184 |
| 33.78 | 0.4 | 0.1343 |
| 34.05 | 0.7 | 0.1761 |
| 34.26 | 1 | 0.3299 |
| 34.75 | 1.2 | 0.2593 |
| 34.95 | 34.95 | 0 |
| 35.45 | 35.45 | 0 |
| 35.49 | 35.49 | 0 |
| 35.52 | 35.52 | 0 |
| 35.56 | 35.56 | 0 |
| 35.61 | 35.61 | 0 |
| 35.73 | 35.73 | 0 |
| 35.8 | 35.8 | 0 |
| 36 | 36 | 0 |
| 36.01 | 36.01 | 0 |
| 36.01 | 36.01 | 0 |
| 36.01 | 36.01 | 0 |
| 36.07 | 36.07 | 0 |
| 36.68 | 2.6 | 0.1217 |
| 37.21 | 37.21 | 0 |
| 37.23 | 37.23 | 0 |
| 37.47 | 37.47 | 0 |
| 37.95 | 69.9 | 0.2387 |
| 38.55 | 38.55 | 0 |
| 38.63 | 0.3 | 0.1217 |
| 38.75 | 0.3 | 0.1217 |
| 39.08 | 0.2 | 0.1217 |
| 39.47 | 0.4 | 0.1217 |
| 39.67 | 0.4 | 0.1217 |
| 39.93 | 39.93 | 0 |
| 39.93 | 39.93 | 0 |
| 40.36 | 40.36 | 0 |
| 40.46 | 40.46 | 0 |
| 40.46 | 40.46 | 0 |
| 40.53 | 40.53 | 0 |
| 41.08 | 2.4 | 0.2947 |
| 41.34 | 2 | 0.2857 |
| 41.61 | 0.4 | 0.2474 |
| 41.92 | 0 | 0.1217 |
| 42.14 | 1.4 | 0.3493 |
| 42.7 | 0.5 | 0.1217 |
| 42.8 | 42.8 | 0 |
| 42.95 | 42.95 | 0 |
| 42.95 | 42.95 | 0 |
| 42.99 | 0.6 | 0.1217 |
| 43.14 | 43.14 |  |
| 43.53 | 1.1 | 0.1217 |
| 43.56 | 43.56 | 0 |
| 44.15 | 38.6 | 0.284 |
| 44.76 | 0 | 0.1217 |
| 45.06 | 1.6 | 0.1217 |
| 45.82 | 0.3 | 0.1217 |
| 46 | 0.5 | 0.1217 |
| 46.3 | 0.2 | 0.1217 |
| 46.56 | 46.56 | 0 |
| 46.61 | 0.3 | 0.1217 |
| 47.02 | 47.02 | 0 |
| 47.06 | 47.06 | 0 |
| 47.23 | 47.23 | 0 |
| 47.35 | 47.35 | 0 |
| 47.62 | 0.1 | 0.0008 |
| 47.91 | 1.1 | 0.3465 |
| 48.07 | 0 | 0.0752 |
| 48.2 | 1.1 | 0.3175 |
| 48.55 | 48.55 | 0 |
| 48.87 | 0.4 | 0.2209 |
| 49.11 | 49.11 | 0 |
| 49.11 | 49.11 | 0 |
| 49.36 | 49.36 | 0 |
| 49.52 | 49.52 | 0 |
| 49.52 | 49.52 | 0 |
| 49.67 | 49.67 | 0 |
| 49.67 | 49.67 | 0 |
| 49.8 | 49.8 | 0 |
| 49.8 | 49.8 | 0 |
| 49.88 | 49.88 | 0 |
| 50.37 | 0.3 | 0.0668 |
| 50.8 | 0.1 | 0.2811 |
| 51.03 | 0.7 | 0.305 |
| 51.48 | 0.2 | 0.132 |
| 51.55 | 51.55 | 0 |
| 51.65 | 0.2 | 0.091 |
| 51.77 | 0.1 | 0.1217 |
| 51.88 | 51.88 | 0 |
| 51.88 | 51.88 | 0 |
| 52.21 | 52.21 | 0 |
| 52.22 | 52.22 | 0 |
| 52.22 | 52.22 | 0 |
| 52.34 | 52.34 | 0 |
| 52.48 | 52.48 | 0 |
| 52.48 | 52.48 | 0 |
| 52.69 | 0.4 | 0.1893 |
| 52.94 | 52.94 | 0 |
| 52.94 | 52.94 | 0 |
| 53.13 | 53.13 | 0 |
| 53.15 | 53.15 | 0 |
| 53.26 | 53.26 | 0 |
| 53.45 | 53.45 | 0 |
| 53.52 | 53.52 | 0 |
| 53.62 | 53.62 | 0 |
| 53.62 | 53.62 | 0 |
| 53.64 | 53.64 | 0 |
| 53.64 | 53.64 | 0 |
| 53.74 | 53.74 | 0 |
| 53.76 | 53.76 | 0 |
| 53.87 | 53.87 | 0 |
| 54.11 | 54.11 | 0 |
| 54.22 | 0.2 | 0.2635 |
| 54.27 | 54.27 | 0 |
| 54.43 | 54.43 | 0 |
| 54.43 | 54.43 | 0 |
| 54.71 | 54.71 | 0 |
| 54.71 | 54.71 | 0 |
| 55.17 | 0.3 | 0.2196 |
| 55.64 | 0.2 | 0.3047 |
| 55.92 | 0.4 | 0.3601 |
| 56.05 | 0.5 | 0.2479 |
| 56.51 | 0.3 | 0.3558 |
| 56.83 | 0.6 | 0.2323 |
| 57.14 | 0.2 | 0.1034 |
| 57.71 | 57.71 | 0 |
| 57.71 | 57.71 | 0 |
| 57.94 | 57.94 | 0 |
| 57.94 | 57.94 | 0 |
| 58.32 | 58.32 | 0 |
| 58.44 | 58.44 | 0 |
| 58.44 | 58.44 | 0 |
| 58.58 | 58.58 | 0 |
| 58.58 | 58.58 | 0 |
| 58.72 | 58.72 | 0 |
| 58.72 | 58.72 | 0 |
| 58.8 | 58.8 | 0 |
| 58.8 | 58.8 | 0 |
| 58.9 | 58.9 | 0 |
| 59.01 | 59.01 | 0 |
| 59.02 | 59.02 | 0 |
| 59.18 | 59.18 | 0 |
| 59.18 | 59.18 | 0 |
| 59.29 | 59.29 | 0 |
| 59.29 | 59.29 | 0 |
| 59.52 | 59.52 | 0 |
| 59.53 | 59.53 | 0 |
| 59.76 | 59.76 | 0 |
| 59.76 | 59.76 | 0 |
| 59.98 | 59.98 | 0 |
| 59.98 | 59.98 | 0 |
| 60.36 | 60.36 | 0 |
| 61.02 | 0.2 | 0.093 |
| 61.49 | 0.2 | 0.0925 |
| 61.79 | 0.1 | 0.0976 |
| 62.05 | 0.3 | 0.2682 |
| 62.33 | 0.5 | 0.1932 |
| 62.49 | 0.3 | 0.1226 |
| 62.8 | 0.6 | 0.2953 |
| 63.09 | 0.4 | 0.2526 |
| 63.24 | 0 | 0.3509 |
| 63.36 | 0.5 | 0.3477 |
| 63.66 | 0.4 | 0.1217 |
| 64.36 | 5.4 | 0.1217 |
| 65.18 | 0.2 | 0.1217 |
| 65.54 | 0.3 | 0.1217 |
| 65.69 | 0.3 | 0.1217 |
| 65.97 | 0.4 | 0.1217 |
| 66.42 | 0.2 | 0.1217 |
| 68.46 | 0.5 | 0.3005 |
| 68.63 | 0.1 | 0.1906 |
| 68.73 | 0.3 | 0.1565 |
| 69.39 | 0.3 | 0.2757 |
| 69.53 | 0.1 | 0.2757 |
| 69.84 | 0.3 | 0.2323 |
| 70.15 | 0.2 | 0.1277 |
| 70.3 | 0.2 | 0.1217 |
| 70.44 | 0.2 | 0.1217 |
| 70.57 | 0.2 | 0.1217 |
| 70.85 | 0.1 | 0.1217 |
| 71.17 | 0.1 | 0.1217 |
| 71.28 | 0.2 | 0.1217 |
| 74 | 0.6 | 0.2846 |
| 76.58 | 0.2 | 0.1217 |
| 77.44 | 10.9 | 0.1217 |
| 78.42 | 0.1 | 0.1217 |
| 81.46 | 0.8 | 0.2243 |
| 81.74 | 0.2 | 0.3116 |
| 81.87 | 0.8 | 0.33 |
| 83.49 | 0.2 | 0.1128 |
| 84.47 | 0.2 | 0.2055 |
| 85.16 | 0.3 | 0.2012 |
| 85.48 | 0.3 | 0.1721 |
| 87.02 | 0.3 | 0.2841 |
| 87.78 | 0.3 | 0.2356 |
| 88.25 | 0.4 | 0.2396 |
| 89.45 | 0.3 | 0.2575 |
| 89.89 | 0.2 | 0.1046 |
| 90.91 | 0.1 | 0.1386 |
| 91.04 | 0.1 | 0.1938 |
| 91.2 | 0.4 | 0.2552 |
| 91.52 | 0.2 | 0.291 |
| 91.67 | 0.1 | 0.2661 |
| 91.83 | 0.4 | 0.2543 |
| 92.06 | 0.4 | 0.2224 |
| 92.38 | 0.7 | 0.269 |
| 92.52 | 0 | 0.1371 |
| 92.69 | 0.7 | 0.2797 |
| 92.93 | 0.1 | 0.1275 |
| 93.05 | 0.4 | 0.2298 |
| 93.36 | 0.4 | 0.2214 |
| 93.49 | 0.2 | 0.1635 |
| 93.84 | 0.6 | 0.2638 |
| 94.21 | 0.5 | 0.2268 |
| 94.44 | 0.2 | 0.0964 |
| 94.73 | 0.5 | 0.3492 |
| 94.83 | 0.1 | 0.1644 |
| 95.01 | 0.4 | 0.2245 |
| 95.31 | 0.2 | 0.2582 |
| 95.44 | 0.3 | 0.3024 |
| 95.88 | 0.2 | 0.1399 |
| 98.23 | 0.1 | 0.2519 |
| 98.35 | 0.5 | 0.3625 |
| 99.98 | 0.3 | 0.3537 |
| 100.14 | 0.2 | 0.3428 |
| 100.37 | 0.2 | 0.131 |
| 100.64 | 0.3 | 0.2117 |
| 100.76 | 0.4 | 0.2794 |
| 101.13 | 0.4 | 0.2371 |
| 101.24 | 0.4 | 0.3339 |
| 101.64 | 0.3 | 0.2767 |
| 101.89 | 0.4 | 0.2516 |
| 102.6 | 0.2 | 0.1306 |
| 105.4 | 0.1 | 0.1217 |
| 111.34 | 3.5 | 0.1217 |
| 111.48 | 3.4 | 0.1217 |
| 112.24 | 0.2 | 0.1217 |
| 113.79 | 0.3 | 0.365 |
| 114.06 | 0 | 0.1217 |
| 114.18 | 0 | 0.365 |
| 114.45 | 0 | 0.0008 |
| 114.71 | 17.1 | 0.365 |
| 115.13 | 78 | 0.1217 |
| 116.77 | 0 | 0.0008 |
| 117.32 | 986.2 | 0.2086 |
| 117.73 | 571 | 0.1411 |
| 118.05 | 188.3 | 0.1124 |
| 118.38 | 1028.4 | 0.2022 |
| 118.51 | 1839.3 | 0.3236 |

**Table S2.** UV-vis absorption spectral data of BTTPNTs on UV-A light irradiation with respect to time.

| **Wavelength (nm)** | **Absorbance (a. u)** | | | | | |
| --- | --- | --- | --- | --- | --- | --- |
|  | **5 min** | **10 min** | **15 min** | **20 min** | **25 min** | **30 min** |
| 200 | 1.549 | 1.569 | 1.549 | 1.593 | 1.579 | 1.567 |
| 200.5 | 1.559 | 1.57 | 1.557 | 1.573 | 1.565 | 1.57 |
| 201 | 1.583 | 1.58 | 1.573 | 1.575 | 1.555 | 1.586 |
| 201.5 | 1.616 | 1.603 | 1.587 | 1.602 | 1.582 | 1.608 |
| 202 | 1.613 | 1.627 | 1.61 | 1.626 | 1.609 | 1.635 |
| 202.5 | 1.611 | 1.641 | 1.637 | 1.652 | 1.63 | 1.649 |
| 203 | 1.643 | 1.647 | 1.637 | 1.657 | 1.665 | 1.662 |
| 203.5 | 1.659 | 1.666 | 1.662 | 1.672 | 1.686 | 1.668 |
| 204 | 1.692 | 1.674 | 1.68 | 1.698 | 1.683 | 1.695 |
| 204.5 | 1.71 | 1.688 | 1.677 | 1.71 | 1.704 | 1.707 |
| 205 | 1.715 | 1.693 | 1.702 | 1.715 | 1.705 | 1.714 |
| 205.5 | 1.725 | 1.712 | 1.717 | 1.713 | 1.707 | 1.741 |
| 206 | 1.735 | 1.754 | 1.729 | 1.731 | 1.724 | 1.755 |
| 206.5 | 1.765 | 1.763 | 1.761 | 1.76 | 1.746 | 1.777 |
| 207 | 1.776 | 1.785 | 1.776 | 1.776 | 1.785 | 1.766 |
| 207.5 | 1.768 | 1.806 | 1.779 | 1.8 | 1.783 | 1.76 |
| 208 | 1.769 | 1.803 | 1.78 | 1.808 | 1.788 | 1.791 |
| 208.5 | 1.79 | 1.82 | 1.784 | 1.81 | 1.791 | 1.822 |
| 209 | 1.811 | 1.817 | 1.806 | 1.825 | 1.797 | 1.832 |
| 209.5 | 1.82 | 1.821 | 1.818 | 1.818 | 1.82 | 1.832 |
| 210 | 1.829 | 1.838 | 1.825 | 1.826 | 1.838 | 1.841 |
| 210.5 | 1.845 | 1.834 | 1.836 | 1.838 | 1.845 | 1.862 |
| 211 | 1.856 | 1.849 | 1.856 | 1.836 | 1.847 | 1.882 |
| 211.5 | 1.866 | 1.862 | 1.864 | 1.85 | 1.854 | 1.886 |
| 212 | 1.874 | 1.878 | 1.85 | 1.866 | 1.866 | 1.882 |
| 212.5 | 1.888 | 1.886 | 1.849 | 1.882 | 1.87 | 1.892 |
| 213 | 1.9 | 1.886 | 1.856 | 1.896 | 1.878 | 1.892 |
| 213.5 | 1.896 | 1.892 | 1.866 | 1.898 | 1.886 | 1.89 |
| 214 | 1.894 | 1.9 | 1.882 | 1.894 | 1.894 | 1.902 |
| 214.5 | 1.892 | 1.902 | 1.89 | 1.896 | 1.888 | 1.913 |
| 215 | 1.9 | 1.907 | 1.888 | 1.892 | 1.866 | 1.913 |
| 215.5 | 1.9 | 1.886 | 1.894 | 1.892 | 1.876 | 1.931 |
| 216 | 1.876 | 1.88 | 1.892 | 1.898 | 1.888 | 1.938 |
| 216.5 | 1.87 | 1.884 | 1.888 | 1.9 | 1.89 | 1.909 |
| 217 | 1.878 | 1.878 | 1.89 | 1.89 | 1.9 | 1.896 |
| 217.5 | 1.874 | 1.878 | 1.876 | 1.874 | 1.884 | 1.913 |
| 218 | 1.87 | 1.866 | 1.862 | 1.882 | 1.858 | 1.918 |
| 218.5 | 1.853 | 1.874 | 1.866 | 1.874 | 1.868 | 1.918 |
| 219 | 1.851 | 1.87 | 1.862 | 1.868 | 1.864 | 1.92 |
| 219.5 | 1.862 | 1.852 | 1.836 | 1.855 | 1.843 | 1.915 |
| 220 | 1.831 | 1.838 | 1.823 | 1.828 | 1.825 | 1.903 |
| 220.5 | 1.804 | 1.807 | 1.816 | 1.825 | 1.813 | 1.878 |
| 221 | 1.788 | 1.798 | 1.792 | 1.807 | 1.803 | 1.866 |
| 221.5 | 1.782 | 1.8 | 1.774 | 1.791 | 1.791 | 1.862 |
| 222 | 1.771 | 1.768 | 1.765 | 1.77 | 1.776 | 1.852 |
| 222.5 | 1.734 | 1.745 | 1.748 | 1.738 | 1.743 | 1.822 |
| 223 | 1.722 | 1.723 | 1.731 | 1.722 | 1.725 | 1.79 |
| 223.5 | 1.71 | 1.698 | 1.709 | 1.703 | 1.717 | 1.782 |
| 224 | 1.678 | 1.685 | 1.683 | 1.686 | 1.686 | 1.763 |
| 224.5 | 1.658 | 1.657 | 1.668 | 1.672 | 1.659 | 1.738 |
| 225 | 1.637 | 1.636 | 1.645 | 1.654 | 1.647 | 1.724 |
| 225.5 | 1.613 | 1.621 | 1.623 | 1.632 | 1.626 | 1.699 |
| 226 | 1.592 | 1.59 | 1.601 | 1.604 | 1.596 | 1.672 |
| 226.5 | 1.556 | 1.558 | 1.57 | 1.569 | 1.567 | 1.646 |
| 227 | 1.512 | 1.526 | 1.535 | 1.533 | 1.539 | 1.607 |
| 227.5 | 1.476 | 1.49 | 1.493 | 1.496 | 1.503 | 1.568 |
| 228 | 1.443 | 1.451 | 1.454 | 1.458 | 1.46 | 1.523 |
| 228.5 | 1.401 | 1.408 | 1.415 | 1.417 | 1.415 | 1.477 |
| 229 | 1.349 | 1.361 | 1.364 | 1.366 | 1.367 | 1.433 |
| 229.5 | 1.292 | 1.304 | 1.309 | 1.309 | 1.312 | 1.374 |
| 230 | 1.232 | 1.246 | 1.249 | 1.246 | 1.252 | 1.311 |
| 230.5 | 1.173 | 1.187 | 1.184 | 1.184 | 1.188 | 1.245 |
| 231 | 1.107 | 1.114 | 1.119 | 1.118 | 1.122 | 1.176 |
| 231.5 | 1.03 | 1.039 | 1.044 | 1.044 | 1.049 | 1.101 |
| 232 | 0.952 | 0.963 | 0.964 | 0.964 | 0.969 | 1.02 |
| 232.5 | 0.874 | 0.883 | 0.884 | 0.882 | 0.888 | 0.939 |
| 233 | 0.796 | 0.803 | 0.803 | 0.802 | 0.806 | 0.857 |
| 233.5 | 0.719 | 0.724 | 0.724 | 0.723 | 0.728 | 0.776 |
| 234 | 0.645 | 0.65 | 0.649 | 0.649 | 0.654 | 0.7 |
| 234.5 | 0.577 | 0.58 | 0.579 | 0.58 | 0.584 | 0.628 |
| 235 | 0.513 | 0.514 | 0.513 | 0.513 | 0.518 | 0.561 |
| 235.5 | 0.452 | 0.451 | 0.45 | 0.45 | 0.455 | 0.497 |
| 236 | 0.394 | 0.393 | 0.391 | 0.391 | 0.397 | 0.438 |
| 236.5 | 0.339 | 0.338 | 0.336 | 0.336 | 0.341 | 0.381 |
| 237 | 0.287 | 0.285 | 0.283 | 0.284 | 0.288 | 0.327 |
| 237.5 | 0.241 | 0.238 | 0.237 | 0.238 | 0.242 | 0.28 |
| 238 | 0.204 | 0.2 | 0.199 | 0.2 | 0.204 | 0.241 |
| 238.5 | 0.171 | 0.167 | 0.166 | 0.167 | 0.17 | 0.207 |
| 239 | 0.142 | 0.138 | 0.137 | 0.137 | 0.141 | 0.177 |
| 239.5 | 0.117 | 0.112 | 0.112 | 0.112 | 0.116 | 0.152 |
| 240 | 0.095 | 0.091 | 0.09 | 0.091 | 0.094 | 0.129 |
| 240.5 | 0.077 | 0.072 | 0.071 | 0.072 | 0.075 | 0.109 |
| 241 | 0.06 | 0.056 | 0.055 | 0.056 | 0.059 | 0.092 |
| 241.5 | 0.046 | 0.042 | 0.041 | 0.042 | 0.045 | 0.077 |
| 242 | 0.035 | 0.031 | 0.03 | 0.03 | 0.033 | 0.064 |
| 242.5 | 0.025 | 0.021 | 0.02 | 0.021 | 0.023 | 0.054 |
| 243 | 0.018 | 0.014 | 0.013 | 0.013 | 0.016 | 0.045 |
| 243.5 | 0.012 | 0.009 | 0.008 | 0.008 | 0.01 | 0.039 |
| 244 | 0.007 | 0.004 | 0.003 | 0.003 | 0.006 | 0.033 |
| 244.5 | 0.003 | 0 | 0 | 0 | 0.002 | 0.029 |
| 245 | 0.001 | -0.002 | -0.002 | -0.003 | 0 | 0.026 |
| 245.5 | 0 | -0.003 | -0.004 | -0.004 | -0.001 | 0.024 |
| 246 | -0.001 | -0.004 | -0.005 | -0.005 | -0.002 | 0.023 |
| 246.5 | -0.001 | -0.003 | -0.004 | -0.004 | -0.002 | 0.022 |
| 247 | 0 | -0.003 | -0.003 | -0.003 | -0.001 | 0.022 |
| 247.5 | 0.002 | -0.001 | -0.002 | -0.002 | 0 | 0.023 |
| 248 | 0.004 | 0.001 | 0 | 0 | 0.002 | 0.025 |
| 248.5 | 0.006 | 0.004 | 0.003 | 0.003 | 0.005 | 0.027 |
| 249 | 0.009 | 0.007 | 0.007 | 0.006 | 0.008 | 0.029 |
| 249.5 | 0.013 | 0.011 | 0.01 | 0.01 | 0.012 | 0.032 |
| 250 | 0.017 | 0.015 | 0.015 | 0.014 | 0.016 | 0.036 |
| 250.5 | 0.022 | 0.02 | 0.019 | 0.019 | 0.021 | 0.04 |
| 251 | 0.027 | 0.025 | 0.025 | 0.025 | 0.027 | 0.045 |
| 251.5 | 0.032 | 0.031 | 0.03 | 0.03 | 0.032 | 0.05 |
| 252 | 0.038 | 0.037 | 0.036 | 0.036 | 0.038 | 0.055 |
| 252.5 | 0.044 | 0.044 | 0.043 | 0.042 | 0.045 | 0.061 |
| 253 | 0.05 | 0.05 | 0.05 | 0.049 | 0.052 | 0.067 |
| 253.5 | 0.057 | 0.057 | 0.057 | 0.056 | 0.059 | 0.074 |
| 254 | 0.064 | 0.065 | 0.064 | 0.064 | 0.066 | 0.081 |
| 254.5 | 0.072 | 0.072 | 0.071 | 0.072 | 0.074 | 0.088 |
| 255 | 0.081 | 0.081 | 0.08 | 0.08 | 0.083 | 0.097 |
| 255.5 | 0.09 | 0.09 | 0.089 | 0.089 | 0.091 | 0.105 |
| 256 | 0.098 | 0.099 | 0.098 | 0.098 | 0.101 | 0.114 |
| 256.5 | 0.107 | 0.108 | 0.108 | 0.108 | 0.11 | 0.123 |
| 257 | 0.117 | 0.118 | 0.118 | 0.118 | 0.119 | 0.133 |
| 257.5 | 0.126 | 0.127 | 0.127 | 0.127 | 0.129 | 0.142 |
| 258 | 0.135 | 0.137 | 0.137 | 0.136 | 0.138 | 0.151 |
| 258.5 | 0.144 | 0.146 | 0.146 | 0.146 | 0.147 | 0.161 |
| 259 | 0.153 | 0.155 | 0.155 | 0.155 | 0.156 | 0.17 |
| 259.5 | 0.161 | 0.164 | 0.164 | 0.164 | 0.165 | 0.178 |
| 260 | 0.171 | 0.173 | 0.174 | 0.174 | 0.175 | 0.188 |
| 260.5 | 0.18 | 0.183 | 0.184 | 0.183 | 0.185 | 0.198 |
| 261 | 0.19 | 0.193 | 0.194 | 0.193 | 0.195 | 0.208 |
| 261.5 | 0.2 | 0.204 | 0.205 | 0.204 | 0.206 | 0.219 |
| 262 | 0.21 | 0.214 | 0.215 | 0.214 | 0.217 | 0.229 |
| 262.5 | 0.222 | 0.225 | 0.227 | 0.226 | 0.228 | 0.24 |
| 263 | 0.233 | 0.237 | 0.238 | 0.237 | 0.239 | 0.252 |
| 263.5 | 0.244 | 0.248 | 0.249 | 0.249 | 0.251 | 0.263 |
| 264 | 0.255 | 0.26 | 0.26 | 0.26 | 0.262 | 0.274 |
| 264.5 | 0.266 | 0.271 | 0.271 | 0.271 | 0.273 | 0.285 |
| 265 | 0.276 | 0.281 | 0.281 | 0.281 | 0.283 | 0.296 |
| 265.5 | 0.285 | 0.289 | 0.29 | 0.29 | 0.292 | 0.304 |
| 266 | 0.292 | 0.297 | 0.297 | 0.297 | 0.299 | 0.312 |
| 266.5 | 0.298 | 0.302 | 0.303 | 0.303 | 0.305 | 0.318 |
| 267 | 0.302 | 0.307 | 0.309 | 0.308 | 0.31 | 0.323 |
| 267.5 | 0.307 | 0.312 | 0.313 | 0.313 | 0.315 | 0.328 |
| 268 | 0.311 | 0.317 | 0.318 | 0.318 | 0.319 | 0.333 |
| 268.5 | 0.317 | 0.323 | 0.324 | 0.324 | 0.325 | 0.34 |
| 269 | 0.324 | 0.329 | 0.331 | 0.33 | 0.332 | 0.347 |
| 269.5 | 0.332 | 0.337 | 0.339 | 0.339 | 0.34 | 0.355 |
| 270 | 0.34 | 0.346 | 0.348 | 0.347 | 0.349 | 0.364 |
| 270.5 | 0.349 | 0.356 | 0.358 | 0.357 | 0.359 | 0.374 |
| 271 | 0.359 | 0.366 | 0.367 | 0.367 | 0.369 | 0.385 |
| 271.5 | 0.368 | 0.375 | 0.377 | 0.377 | 0.378 | 0.395 |
| 272 | 0.375 | 0.383 | 0.384 | 0.384 | 0.385 | 0.402 |
| 272.5 | 0.379 | 0.386 | 0.388 | 0.387 | 0.389 | 0.406 |
| 273 | 0.38 | 0.387 | 0.389 | 0.388 | 0.39 | 0.407 |
| 273.5 | 0.378 | 0.385 | 0.386 | 0.385 | 0.387 | 0.405 |
| 274 | 0.372 | 0.38 | 0.38 | 0.381 | 0.382 | 0.4 |
| 274.5 | 0.365 | 0.372 | 0.373 | 0.374 | 0.374 | 0.393 |
| 275 | 0.357 | 0.364 | 0.365 | 0.365 | 0.366 | 0.385 |
| 275.5 | 0.349 | 0.355 | 0.356 | 0.356 | 0.357 | 0.377 |
| 276 | 0.339 | 0.346 | 0.346 | 0.346 | 0.348 | 0.368 |
| 276.5 | 0.329 | 0.336 | 0.336 | 0.336 | 0.337 | 0.358 |
| 277 | 0.319 | 0.326 | 0.326 | 0.326 | 0.328 | 0.348 |
| 277.5 | 0.31 | 0.317 | 0.316 | 0.316 | 0.319 | 0.34 |
| 278 | 0.301 | 0.308 | 0.307 | 0.307 | 0.31 | 0.332 |
| 278.5 | 0.294 | 0.3 | 0.3 | 0.3 | 0.302 | 0.324 |
| 279 | 0.288 | 0.294 | 0.293 | 0.293 | 0.295 | 0.319 |
| 279.5 | 0.283 | 0.29 | 0.289 | 0.289 | 0.291 | 0.315 |
| 280 | 0.279 | 0.286 | 0.285 | 0.286 | 0.287 | 0.312 |
| 280.5 | 0.276 | 0.283 | 0.281 | 0.282 | 0.283 | 0.309 |
| 281 | 0.272 | 0.279 | 0.278 | 0.278 | 0.28 | 0.306 |
| 281.5 | 0.267 | 0.274 | 0.273 | 0.274 | 0.275 | 0.301 |
| 282 | 0.262 | 0.268 | 0.266 | 0.267 | 0.269 | 0.295 |
| 282.5 | 0.254 | 0.26 | 0.258 | 0.259 | 0.26 | 0.287 |
| 283 | 0.244 | 0.25 | 0.247 | 0.248 | 0.25 | 0.277 |
| 283.5 | 0.231 | 0.237 | 0.234 | 0.235 | 0.237 | 0.265 |
| 284 | 0.217 | 0.221 | 0.219 | 0.22 | 0.221 | 0.25 |
| 284.5 | 0.2 | 0.204 | 0.202 | 0.204 | 0.205 | 0.234 |
| 285 | 0.182 | 0.187 | 0.185 | 0.185 | 0.186 | 0.216 |
| 285.5 | 0.165 | 0.169 | 0.166 | 0.167 | 0.168 | 0.198 |
| 286 | 0.148 | 0.152 | 0.149 | 0.15 | 0.151 | 0.181 |
| 286.5 | 0.131 | 0.135 | 0.132 | 0.133 | 0.134 | 0.165 |
| 287 | 0.114 | 0.118 | 0.115 | 0.115 | 0.117 | 0.147 |
| 287.5 | 0.098 | 0.103 | 0.099 | 0.099 | 0.101 | 0.132 |
| 288 | 0.083 | 0.088 | 0.085 | 0.084 | 0.086 | 0.118 |
| 288.5 | 0.07 | 0.075 | 0.071 | 0.071 | 0.073 | 0.105 |
| 289 | 0.059 | 0.064 | 0.06 | 0.06 | 0.062 | 0.094 |
| 289.5 | 0.05 | 0.054 | 0.05 | 0.05 | 0.053 | 0.085 |
| 290 | 0.042 | 0.046 | 0.042 | 0.042 | 0.044 | 0.077 |
| 290.5 | 0.035 | 0.04 | 0.035 | 0.036 | 0.038 | 0.071 |
| 291 | 0.029 | 0.034 | 0.03 | 0.03 | 0.032 | 0.065 |
| 291.5 | 0.025 | 0.03 | 0.026 | 0.026 | 0.027 | 0.061 |
| 292 | 0.021 | 0.026 | 0.021 | 0.022 | 0.023 | 0.057 |
| 292.5 | 0.017 | 0.023 | 0.018 | 0.018 | 0.02 | 0.053 |
| 293 | 0.015 | 0.02 | 0.016 | 0.016 | 0.017 | 0.05 |
| 293.5 | 0.012 | 0.018 | 0.014 | 0.014 | 0.015 | 0.048 |
| 294 | 0.011 | 0.016 | 0.012 | 0.012 | 0.014 | 0.046 |
| 294.5 | 0.009 | 0.015 | 0.01 | 0.01 | 0.012 | 0.044 |
| 295 | 0.008 | 0.013 | 0.009 | 0.009 | 0.011 | 0.042 |
| 295.5 | 0.006 | 0.013 | 0.008 | 0.008 | 0.01 | 0.04 |
| 296 | 0.006 | 0.012 | 0.007 | 0.007 | 0.009 | 0.039 |
| 296.5 | 0.005 | 0.011 | 0.006 | 0.006 | 0.008 | 0.038 |
| 297 | 0.004 | 0.011 | 0.006 | 0.006 | 0.007 | 0.037 |
| 297.5 | 0.004 | 0.011 | 0.005 | 0.005 | 0.007 | 0.036 |
| 298 | 0.004 | 0.01 | 0.005 | 0.005 | 0.007 | 0.035 |
| 298.5 | 0.003 | 0.01 | 0.005 | 0.005 | 0.006 | 0.034 |
| 299 | 0.003 | 0.01 | 0.005 | 0.005 | 0.006 | 0.033 |
| 299.5 | 0.003 | 0.01 | 0.005 | 0.005 | 0.006 | 0.032 |
| 300 | 0.002 | 0.01 | 0.005 | 0.004 | 0.006 | 0.031 |
| 300.5 | 0.002 | 0.01 | 0.004 | 0.004 | 0.005 | 0.03 |
| 301 | 0.002 | 0.01 | 0.004 | 0.004 | 0.005 | 0.029 |
| 301.5 | 0.002 | 0.009 | 0.004 | 0.004 | 0.005 | 0.027 |
| 302 | 0.001 | 0.009 | 0.004 | 0.004 | 0.005 | 0.026 |
| 302.5 | 0.001 | 0.009 | 0.004 | 0.003 | 0.005 | 0.025 |
| 303 | 0.001 | 0.009 | 0.004 | 0.004 | 0.005 | 0.024 |
| 303.5 | 0.001 | 0.009 | 0.004 | 0.004 | 0.005 | 0.023 |
| 304 | 0.001 | 0.009 | 0.004 | 0.004 | 0.005 | 0.022 |
| 304.5 | 0.001 | 0.009 | 0.004 | 0.003 | 0.005 | 0.021 |
| 305 | 0.001 | 0.009 | 0.003 | 0.003 | 0.005 | 0.02 |
| 305.5 | 0.001 | 0.009 | 0.004 | 0.004 | 0.005 | 0.019 |
| 306 | 0.001 | 0.01 | 0.004 | 0.004 | 0.005 | 0.018 |
| 306.5 | 0.001 | 0.01 | 0.004 | 0.004 | 0.005 | 0.017 |
| 307 | 0.001 | 0.01 | 0.004 | 0.004 | 0.005 | 0.017 |
| 307.5 | 0.001 | 0.01 | 0.004 | 0.004 | 0.005 | 0.016 |
| 308 | 0.001 | 0.01 | 0.004 | 0.004 | 0.005 | 0.016 |
| 308.5 | 0.001 | 0.01 | 0.004 | 0.004 | 0.005 | 0.015 |
| 309 | 0 | 0.01 | 0.004 | 0.004 | 0.005 | 0.014 |
| 309.5 | 0 | 0.01 | 0.004 | 0.004 | 0.005 | 0.013 |
| 310 | 0.001 | 0.01 | 0.004 | 0.004 | 0.005 | 0.013 |
| 310.5 | 0.001 | 0.01 | 0.004 | 0.004 | 0.005 | 0.012 |
| 311 | 0 | 0.01 | 0.004 | 0.004 | 0.005 | 0.011 |
| 311.5 | 0 | 0.01 | 0.004 | 0.004 | 0.005 | 0.011 |
| 312 | 0 | 0.01 | 0.004 | 0.004 | 0.005 | 0.01 |
| 312.5 | 0 | 0.01 | 0.004 | 0.004 | 0.005 | 0.01 |
| 313 | 0 | 0.01 | 0.004 | 0.004 | 0.005 | 0.01 |
| 313.5 | 0 | 0.01 | 0.004 | 0.003 | 0.005 | 0.009 |
| 314 | 0 | 0.01 | 0.004 | 0.004 | 0.005 | 0.009 |
| 314.5 | 0 | 0.01 | 0.004 | 0.004 | 0.004 | 0.009 |
| 315 | 0 | 0.01 | 0.004 | 0.003 | 0.004 | 0.008 |
| 315.5 | 0 | 0.01 | 0.004 | 0.004 | 0.005 | 0.008 |
| 316 | 0 | 0.01 | 0.004 | 0.004 | 0.005 | 0.008 |
| 316.5 | 0 | 0.01 | 0.004 | 0.004 | 0.005 | 0.008 |
| 317 | 0 | 0.01 | 0.004 | 0.004 | 0.005 | 0.008 |
| 317.5 | 0 | 0.01 | 0.004 | 0.003 | 0.004 | 0.008 |
| 318 | 0 | 0.01 | 0.004 | 0.004 | 0.005 | 0.007 |
| 318.5 | 0 | 0.01 | 0.004 | 0.004 | 0.005 | 0.007 |
| 319 | 0 | 0.01 | 0.004 | 0.004 | 0.005 | 0.007 |
| 319.5 | 0 | 0.01 | 0.004 | 0.004 | 0.005 | 0.007 |
| 320 | 0 | 0.01 | 0.004 | 0.004 | 0.005 | 0.007 |
| 320.5 | 0 | 0.01 | 0.004 | 0.004 | 0.005 | 0.007 |
| 321 | 0 | 0.01 | 0.004 | 0.004 | 0.005 | 0.007 |
| 321.5 | 0 | 0.01 | 0.004 | 0.004 | 0.005 | 0.007 |
| 322 | 0 | 0.01 | 0.004 | 0.004 | 0.005 | 0.007 |
| 322.5 | 0 | 0.01 | 0.004 | 0.003 | 0.005 | 0.007 |
| 323 | 0 | 0.01 | 0.004 | 0.003 | 0.004 | 0.007 |
| 323.5 | -0.001 | 0.01 | 0.004 | 0.003 | 0.004 | 0.006 |
| 324 | -0.001 | 0.01 | 0.004 | 0.003 | 0.004 | 0.006 |
| 324.5 | -0.001 | 0.01 | 0.004 | 0.004 | 0.004 | 0.006 |
| 325 | -0.001 | 0.009 | 0.004 | 0.004 | 0.004 | 0.006 |
| 325.5 | 0 | 0.009 | 0.004 | 0.003 | 0.004 | 0.007 |
| 326 | 0 | 0.01 | 0.004 | 0.003 | 0.004 | 0.006 |
| 326.5 | -0.001 | 0.01 | 0.004 | 0.003 | 0.004 | 0.006 |
| 327 | -0.001 | 0.01 | 0.004 | 0.004 | 0.004 | 0.006 |
| 327.5 | 0 | 0.01 | 0.004 | 0.004 | 0.004 | 0.006 |
| 328 | -0.001 | 0.01 | 0.004 | 0.004 | 0.004 | 0.006 |
| 328.5 | -0.001 | 0.009 | 0.004 | 0.003 | 0.004 | 0.005 |
| 329 | -0.001 | 0.009 | 0.004 | 0.003 | 0.004 | 0.005 |
| 329.5 | -0.001 | 0.009 | 0.004 | 0.003 | 0.004 | 0.005 |
| 330 | -0.001 | 0.009 | 0.004 | 0.003 | 0.004 | 0.005 |
| 330.5 | -0.001 | 0.009 | 0.004 | 0.003 | 0.004 | 0.005 |
| 331 | 0 | 0.01 | 0.004 | 0.004 | 0.005 | 0.006 |
| 331.5 | 0 | 0.01 | 0.004 | 0.004 | 0.005 | 0.006 |
| 332 | -0.001 | 0.01 | 0.004 | 0.003 | 0.004 | 0.005 |
| 332.5 | -0.001 | 0.009 | 0.004 | 0.003 | 0.004 | 0.005 |
| 333 | -0.001 | 0.009 | 0.003 | 0.003 | 0.004 | 0.005 |
| 333.5 | -0.001 | 0.009 | 0.003 | 0.003 | 0.003 | 0.005 |
| 334 | -0.001 | 0.009 | 0.004 | 0.003 | 0.004 | 0.005 |
| 334.5 | -0.001 | 0.009 | 0.004 | 0.003 | 0.004 | 0.005 |
| 335 | -0.001 | 0.009 | 0.003 | 0.003 | 0.004 | 0.005 |
| 335.5 | -0.001 | 0.009 | 0.003 | 0.003 | 0.004 | 0.005 |
| 336 | -0.002 | 0.009 | 0.003 | 0.003 | 0.004 | 0.005 |
| 336.5 | -0.002 | 0.009 | 0.003 | 0.003 | 0.004 | 0.005 |
| 337 | -0.001 | 0.009 | 0.003 | 0.003 | 0.003 | 0.005 |
| 337.5 | -0.002 | 0.009 | 0.003 | 0.003 | 0.003 | 0.005 |
| 338 | -0.002 | 0.009 | 0.003 | 0.003 | 0.003 | 0.004 |
| 338.5 | -0.002 | 0.009 | 0.003 | 0.003 | 0.003 | 0.004 |
| 339 | -0.002 | 0.009 | 0.003 | 0.003 | 0.003 | 0.004 |
| 339.5 | -0.002 | 0.008 | 0.003 | 0.003 | 0.003 | 0.004 |
| 340 | -0.002 | 0.008 | 0.003 | 0.003 | 0.003 | 0.004 |
| 340.5 | -0.002 | 0.008 | 0.003 | 0.003 | 0.003 | 0.004 |
| 341 | -0.002 | 0.008 | 0.003 | 0.003 | 0.004 | 0.004 |
| 341.5 | -0.002 | 0.008 | 0.003 | 0.002 | 0.004 | 0.004 |
| 342 | -0.002 | 0.008 | 0.003 | 0.002 | 0.003 | 0.004 |
| 342.5 | -0.002 | 0.008 | 0.003 | 0.003 | 0.003 | 0.004 |
| 343 | -0.002 | 0.008 | 0.003 | 0.003 | 0.003 | 0.004 |
| 343.5 | -0.002 | 0.008 | 0.003 | 0.002 | 0.003 | 0.004 |
| 344 | -0.002 | 0.007 | 0.002 | 0.002 | 0.003 | 0.003 |
| 344.5 | -0.003 | 0.007 | 0.002 | 0.002 | 0.003 | 0.003 |
| 345 | -0.003 | 0.008 | 0.003 | 0.002 | 0.003 | 0.004 |
| 345.5 | -0.002 | 0.008 | 0.003 | 0.002 | 0.003 | 0.003 |
| 346 | -0.003 | 0.007 | 0.003 | 0.002 | 0.003 | 0.003 |
| 346.5 | -0.003 | 0.007 | 0.003 | 0.002 | 0.003 | 0.003 |
| 347 | -0.003 | 0.007 | 0.003 | 0.002 | 0.003 | 0.003 |
| 347.5 | -0.003 | 0.007 | 0.003 | 0.002 | 0.003 | 0.003 |
| 348 | -0.003 | 0.007 | 0.003 | 0.002 | 0.003 | 0.003 |
| 348.5 | -0.002 | 0.007 | 0.003 | 0.002 | 0.003 | 0.003 |
| 349 | -0.002 | 0.008 | 0.003 | 0.002 | 0.003 | 0.004 |
| 349.5 | -0.002 | 0.008 | 0.003 | 0.002 | 0.003 | 0.004 |
| 350 | -0.003 | 0.007 | 0.003 | 0.003 | 0.003 | 0.003 |
| 350.5 | -0.003 | 0.007 | 0.003 | 0.002 | 0.003 | 0.003 |
| 351 | -0.002 | 0.007 | 0.003 | 0.002 | 0.003 | 0.003 |
| 351.5 | -0.003 | 0.007 | 0.003 | 0.003 | 0.003 | 0.003 |
| 352 | -0.002 | 0.007 | 0.003 | 0.003 | 0.003 | 0.003 |
| 352.5 | -0.002 | 0.007 | 0.003 | 0.003 | 0.003 | 0.003 |
| 353 | -0.002 | 0.007 | 0.003 | 0.003 | 0.003 | 0.003 |
| 353.5 | -0.002 | 0.007 | 0.003 | 0.003 | 0.003 | 0.003 |
| 354 | -0.003 | 0.007 | 0.003 | 0.002 | 0.003 | 0.003 |
| 354.5 | -0.003 | 0.007 | 0.003 | 0.002 | 0.003 | 0.003 |
| 355 | -0.002 | 0.007 | 0.003 | 0.002 | 0.003 | 0.003 |
| 355.5 | -0.002 | 0.007 | 0.003 | 0.002 | 0.003 | 0.003 |
| 356 | -0.002 | 0.007 | 0.003 | 0.002 | 0.003 | 0.003 |
| 356.5 | -0.002 | 0.007 | 0.003 | 0.002 | 0.003 | 0.003 |
| 357 | -0.002 | 0.007 | 0.003 | 0.002 | 0.003 | 0.003 |
| 357.5 | -0.002 | 0.007 | 0.003 | 0.003 | 0.003 | 0.003 |
| 358 | -0.002 | 0.006 | 0.003 | 0.003 | 0.003 | 0.003 |
| 358.5 | -0.002 | 0.006 | 0.003 | 0.003 | 0.003 | 0.003 |
| 359 | -0.003 | 0.006 | 0.003 | 0.002 | 0.003 | 0.003 |
| 359.5 | -0.002 | 0.006 | 0.003 | 0.002 | 0.003 | 0.003 |
| 360 | -0.003 | 0.006 | 0.003 | 0.002 | 0.003 | 0.003 |
| 360.5 | -0.003 | 0.006 | 0.003 | 0.002 | 0.003 | 0.003 |
| 361 | -0.003 | 0.006 | 0.003 | 0.002 | 0.003 | 0.003 |
| 361.5 | -0.002 | 0.006 | 0.003 | 0.002 | 0.003 | 0.003 |
| 362 | -0.002 | 0.006 | 0.003 | 0.002 | 0.003 | 0.003 |
| 362.5 | -0.002 | 0.006 | 0.003 | 0.002 | 0.003 | 0.003 |
| 363 | -0.002 | 0.006 | 0.003 | 0.002 | 0.003 | 0.003 |
| 363.5 | -0.002 | 0.006 | 0.002 | 0.002 | 0.003 | 0.003 |
| 364 | -0.003 | 0.006 | 0.003 | 0.002 | 0.003 | 0.003 |
| 364.5 | -0.003 | 0.005 | 0.003 | 0.002 | 0.003 | 0.003 |
| 365 | -0.003 | 0.005 | 0.002 | 0.002 | 0.003 | 0.003 |
| 365.5 | -0.003 | 0.005 | 0.002 | 0.002 | 0.003 | 0.003 |
| 366 | -0.002 | 0.005 | 0.003 | 0.002 | 0.003 | 0.003 |
| 366.5 | -0.002 | 0.006 | 0.003 | 0.002 | 0.003 | 0.003 |
| 367 | -0.002 | 0.006 | 0.003 | 0.002 | 0.003 | 0.004 |
| 367.5 | -0.003 | 0.006 | 0.003 | 0.002 | 0.003 | 0.004 |
| 368 | -0.002 | 0.005 | 0.003 | 0.002 | 0.003 | 0.004 |
| 368.5 | -0.002 | 0.005 | 0.003 | 0.002 | 0.003 | 0.004 |
| 369 | -0.003 | 0.005 | 0.003 | 0.002 | 0.003 | 0.004 |
| 369.5 | -0.003 | 0.005 | 0.002 | 0.002 | 0.002 | 0.003 |
| 370 | -0.003 | 0.005 | 0.002 | 0.002 | 0.003 | 0.003 |
| 370.5 | -0.003 | 0.005 | 0.003 | 0.002 | 0.003 | 0.003 |
| 371 | -0.003 | 0.005 | 0.003 | 0.002 | 0.003 | 0.004 |
| 371.5 | -0.003 | 0.005 | 0.003 | 0.002 | 0.003 | 0.004 |
| 372 | -0.003 | 0.005 | 0.003 | 0.002 | 0.003 | 0.003 |
| 372.5 | -0.003 | 0.005 | 0.003 | 0.002 | 0.003 | 0.004 |
| 373 | -0.002 | 0.005 | 0.003 | 0.002 | 0.003 | 0.004 |
| 373.5 | -0.003 | 0.005 | 0.003 | 0.002 | 0.003 | 0.003 |
| 374 | -0.003 | 0.005 | 0.002 | 0.002 | 0.003 | 0.003 |
| 374.5 | -0.003 | 0.005 | 0.003 | 0.002 | 0.003 | 0.003 |
| 375 | -0.003 | 0.005 | 0.003 | 0.002 | 0.003 | 0.003 |
| 375.5 | -0.003 | 0.005 | 0.003 | 0.002 | 0.003 | 0.003 |
| 376 | -0.003 | 0.005 | 0.003 | 0.002 | 0.003 | 0.003 |
| 376.5 | -0.003 | 0.005 | 0.003 | 0.002 | 0.003 | 0.003 |
| 377 | -0.003 | 0.005 | 0.003 | 0.002 | 0.003 | 0.003 |
| 377.5 | -0.003 | 0.005 | 0.003 | 0.002 | 0.003 | 0.003 |
| 378 | -0.003 | 0.004 | 0.002 | 0.002 | 0.003 | 0.003 |
| 378.5 | -0.003 | 0.004 | 0.002 | 0.002 | 0.003 | 0.003 |
| 379 | -0.003 | 0.005 | 0.003 | 0.002 | 0.003 | 0.003 |
| 379.5 | -0.003 | 0.004 | 0.002 | 0.002 | 0.003 | 0.003 |
| 380 | -0.003 | 0.004 | 0.002 | 0.002 | 0.003 | 0.003 |
| 380.5 | -0.003 | 0.005 | 0.003 | 0.002 | 0.003 | 0.003 |
| 381 | -0.003 | 0.004 | 0.003 | 0.002 | 0.003 | 0.003 |
| 381.5 | -0.003 | 0.004 | 0.003 | 0.002 | 0.003 | 0.003 |
| 382 | -0.003 | 0.004 | 0.003 | 0.002 | 0.003 | 0.003 |
| 382.5 | -0.003 | 0.004 | 0.003 | 0.002 | 0.003 | 0.003 |
| 383 | -0.003 | 0.004 | 0.002 | 0.002 | 0.003 | 0.003 |
| 383.5 | -0.003 | 0.004 | 0.002 | 0.002 | 0.002 | 0.003 |
| 384 | -0.003 | 0.004 | 0.002 | 0.002 | 0.002 | 0.003 |
| 384.5 | -0.003 | 0.004 | 0.002 | 0.002 | 0.002 | 0.003 |
| 385 | -0.003 | 0.004 | 0.002 | 0.002 | 0.002 | 0.003 |
| 385.5 | -0.003 | 0.004 | 0.002 | 0.002 | 0.002 | 0.003 |
| 386 | -0.003 | 0.004 | 0.002 | 0.002 | 0.002 | 0.003 |
| 386.5 | -0.003 | 0.004 | 0.003 | 0.002 | 0.003 | 0.003 |
| 387 | -0.003 | 0.004 | 0.003 | 0.002 | 0.003 | 0.003 |
| 387.5 | -0.003 | 0.004 | 0.003 | 0.002 | 0.003 | 0.003 |
| 388 | -0.003 | 0.004 | 0.003 | 0.002 | 0.003 | 0.003 |
| 388.5 | -0.003 | 0.004 | 0.003 | 0.002 | 0.003 | 0.003 |
| 389 | -0.003 | 0.004 | 0.002 | 0.002 | 0.003 | 0.003 |
| 389.5 | -0.003 | 0.004 | 0.002 | 0.002 | 0.003 | 0.003 |
| 390 | -0.003 | 0.004 | 0.002 | 0.002 | 0.003 | 0.003 |
| 390.5 | -0.003 | 0.004 | 0.002 | 0.002 | 0.003 | 0.003 |
| 391 | -0.003 | 0.004 | 0.002 | 0.002 | 0.003 | 0.003 |
| 391.5 | -0.003 | 0.004 | 0.002 | 0.002 | 0.003 | 0.003 |
| 392 | -0.003 | 0.004 | 0.003 | 0.002 | 0.003 | 0.003 |
| 392.5 | -0.003 | 0.004 | 0.002 | 0.002 | 0.003 | 0.003 |
| 393 | -0.003 | 0.004 | 0.002 | 0.002 | 0.003 | 0.003 |
| 393.5 | -0.003 | 0.004 | 0.002 | 0.002 | 0.003 | 0.003 |
| 394 | -0.003 | 0.004 | 0.002 | 0.002 | 0.002 | 0.003 |
| 394.5 | -0.003 | 0.004 | 0.002 | 0.002 | 0.002 | 0.003 |
| 395 | -0.003 | 0.004 | 0.002 | 0.002 | 0.002 | 0.003 |
| 395.5 | -0.003 | 0.004 | 0.002 | 0.002 | 0.003 | 0.003 |
| 396 | -0.003 | 0.004 | 0.002 | 0.002 | 0.003 | 0.003 |
| 396.5 | -0.003 | 0.004 | 0.002 | 0.002 | 0.003 | 0.003 |
| 397 | -0.003 | 0.004 | 0.002 | 0.002 | 0.002 | 0.003 |
| 397.5 | -0.003 | 0.004 | 0.002 | 0.002 | 0.002 | 0.003 |
| 398 | -0.003 | 0.004 | 0.002 | 0.002 | 0.002 | 0.003 |
| 398.5 | -0.003 | 0.004 | 0.002 | 0.002 | 0.003 | 0.003 |
| 399 | -0.003 | 0.004 | 0.002 | 0.002 | 0.003 | 0.003 |
| 399.5 | -0.003 | 0.004 | 0.002 | 0.002 | 0.003 | 0.003 |
| 400 | -0.003 | 0.004 | 0.002 | 0.002 | 0.003 | 0.003 |
| 400.5 | -0.003 | 0.004 | 0.002 | 0.002 | 0.003 | 0.003 |
| 401 | -0.003 | 0.004 | 0.002 | 0.002 | 0.003 | 0.003 |
| 401.5 | -0.003 | 0.004 | 0.002 | 0.002 | 0.003 | 0.003 |
| 402 | -0.003 | 0.004 | 0.002 | 0.002 | 0.003 | 0.003 |
| 402.5 | -0.003 | 0.004 | 0.002 | 0.002 | 0.003 | 0.003 |
| 403 | -0.003 | 0.004 | 0.002 | 0.002 | 0.002 | 0.003 |
| 403.5 | -0.003 | 0.004 | 0.002 | 0.002 | 0.002 | 0.003 |
| 404 | -0.003 | 0.004 | 0.002 | 0.002 | 0.002 | 0.003 |
| 404.5 | -0.003 | 0.004 | 0.002 | 0.002 | 0.002 | 0.003 |
| 405 | -0.003 | 0.004 | 0.002 | 0.002 | 0.003 | 0.003 |
| 405.5 | -0.003 | 0.004 | 0.002 | 0.002 | 0.003 | 0.003 |
| 406 | -0.003 | 0.004 | 0.002 | 0.002 | 0.003 | 0.002 |
| 406.5 | -0.003 | 0.004 | 0.002 | 0.002 | 0.003 | 0.002 |
| 407 | -0.003 | 0.004 | 0.002 | 0.002 | 0.003 | 0.002 |
| 407.5 | -0.003 | 0.004 | 0.002 | 0.002 | 0.003 | 0.002 |
| 408 | -0.003 | 0.004 | 0.002 | 0.002 | 0.002 | 0.002 |
| 408.5 | -0.003 | 0.004 | 0.002 | 0.002 | 0.002 | 0.002 |
| 409 | -0.003 | 0.004 | 0.002 | 0.002 | 0.002 | 0.002 |
| 409.5 | -0.003 | 0.004 | 0.002 | 0.002 | 0.002 | 0.002 |
| 410 | -0.003 | 0.004 | 0.002 | 0.002 | 0.002 | 0.002 |
| 410.5 | -0.003 | 0.004 | 0.002 | 0.002 | 0.003 | 0.002 |
| 411 | -0.003 | 0.004 | 0.002 | 0.002 | 0.003 | 0.002 |
| 411.5 | -0.003 | 0.004 | 0.002 | 0.002 | 0.002 | 0.002 |
| 412 | -0.003 | 0.004 | 0.002 | 0.002 | 0.002 | 0.002 |
| 412.5 | -0.003 | 0.004 | 0.002 | 0.002 | 0.002 | 0.002 |
| 413 | -0.003 | 0.004 | 0.002 | 0.002 | 0.002 | 0.002 |
| 413.5 | -0.003 | 0.004 | 0.002 | 0.002 | 0.002 | 0.002 |
| 414 | -0.003 | 0.004 | 0.002 | 0.002 | 0.002 | 0.002 |
| 414.5 | -0.003 | 0.004 | 0.002 | 0.002 | 0.002 | 0.002 |
| 415 | -0.003 | 0.004 | 0.002 | 0.002 | 0.003 | 0.002 |
| 415.5 | -0.003 | 0.004 | 0.002 | 0.002 | 0.002 | 0.002 |
| 416 | -0.003 | 0.004 | 0.002 | 0.002 | 0.002 | 0.002 |
| 416.5 | -0.003 | 0.004 | 0.002 | 0.002 | 0.002 | 0.002 |
| 417 | -0.003 | 0.004 | 0.002 | 0.002 | 0.002 | 0.002 |
| 417.5 | -0.003 | 0.004 | 0.002 | 0.002 | 0.002 | 0.001 |
| 418 | -0.003 | 0.004 | 0.002 | 0.002 | 0.002 | 0.001 |
| 418.5 | -0.003 | 0.004 | 0.002 | 0.002 | 0.002 | 0.001 |
| 419 | -0.003 | 0.004 | 0.002 | 0.002 | 0.002 | 0.001 |
| 419.5 | -0.003 | 0.004 | 0.002 | 0.002 | 0.002 | 0.001 |
| 420 | -0.003 | 0.004 | 0.002 | 0.002 | 0.002 | 0.001 |
| 420.5 | -0.003 | 0.004 | 0.002 | 0.002 | 0.002 | 0.001 |
| 421 | -0.003 | 0.004 | 0.002 | 0.002 | 0.002 | 0.001 |
| 421.5 | -0.003 | 0.004 | 0.002 | 0.002 | 0.002 | 0.001 |
| 422 | -0.003 | 0.004 | 0.002 | 0.002 | 0.002 | 0.001 |
| 422.5 | -0.003 | 0.004 | 0.002 | 0.002 | 0.002 | 0.001 |
| 423 | -0.003 | 0.004 | 0.002 | 0.002 | 0.002 | 0.001 |
| 423.5 | -0.003 | 0.004 | 0.002 | 0.002 | 0.002 | 0.001 |
| 424 | -0.003 | 0.004 | 0.002 | 0.002 | 0.002 | 0.001 |
| 424.5 | -0.003 | 0.004 | 0.002 | 0.002 | 0.002 | 0.001 |
| 425 | -0.003 | 0.004 | 0.002 | 0.002 | 0.002 | 0.001 |
| 425.5 | -0.003 | 0.004 | 0.002 | 0.002 | 0.002 | 0.001 |
| 426 | -0.003 | 0.004 | 0.002 | 0.002 | 0.002 | 0.001 |
| 426.5 | -0.003 | 0.004 | 0.002 | 0.002 | 0.002 | 0.001 |
| 427 | -0.003 | 0.004 | 0.002 | 0.002 | 0.002 | 0.001 |
| 427.5 | -0.003 | 0.004 | 0.002 | 0.002 | 0.002 | 0.001 |
| 428 | -0.003 | 0.004 | 0.002 | 0.002 | 0.002 | 0.001 |
| 428.5 | -0.003 | 0.004 | 0.002 | 0.002 | 0.002 | 0.001 |
| 429 | -0.003 | 0.004 | 0.002 | 0.002 | 0.002 | 0.001 |
| 429.5 | -0.003 | 0.004 | 0.002 | 0.002 | 0.002 | 0.001 |
| 430 | -0.003 | 0.004 | 0.002 | 0.002 | 0.002 | 0.001 |
| 430.5 | -0.003 | 0.004 | 0.002 | 0.002 | 0.003 | 0.001 |
| 431 | -0.002 | 0.004 | 0.003 | 0.002 | 0.003 | 0.001 |
| 431.5 | -0.003 | 0.004 | 0.002 | 0.002 | 0.003 | 0.001 |
| 432 | -0.003 | 0.004 | 0.002 | 0.002 | 0.002 | 0.001 |
| 432.5 | -0.003 | 0.004 | 0.002 | 0.002 | 0.002 | 0.001 |
| 433 | -0.003 | 0.004 | 0.002 | 0.002 | 0.002 | 0.001 |
| 433.5 | -0.003 | 0.004 | 0.002 | 0.002 | 0.002 | 0.001 |
| 434 | -0.003 | 0.004 | 0.002 | 0.002 | 0.002 | 0.001 |
| 434.5 | -0.003 | 0.004 | 0.002 | 0.002 | 0.002 | 0.001 |
| 435 | -0.003 | 0.004 | 0.002 | 0.002 | 0.002 | 0.001 |
| 435.5 | -0.003 | 0.004 | 0.002 | 0.002 | 0.002 | 0.001 |
| 436 | -0.003 | 0.004 | 0.002 | 0.002 | 0.002 | 0.001 |
| 436.5 | -0.003 | 0.004 | 0.002 | 0.002 | 0.002 | 0.001 |
| 437 | -0.003 | 0.004 | 0.002 | 0.002 | 0.002 | 0.001 |
| 437.5 | -0.003 | 0.004 | 0.002 | 0.002 | 0.002 | 0.001 |
| 438 | -0.003 | 0.004 | 0.002 | 0.002 | 0.002 | 0.001 |
| 438.5 | -0.003 | 0.004 | 0.002 | 0.002 | 0.002 | 0.001 |
| 439 | -0.003 | 0.004 | 0.002 | 0.002 | 0.002 | 0.001 |
| 439.5 | -0.003 | 0.004 | 0.002 | 0.002 | 0.002 | 0.001 |
| 440 | -0.003 | 0.004 | 0.002 | 0.002 | 0.002 | 0.001 |
| 440.5 | -0.003 | 0.004 | 0.002 | 0.002 | 0.002 | 0.001 |
| 441 | -0.003 | 0.004 | 0.002 | 0.002 | 0.002 | 0.001 |
| 441.5 | -0.003 | 0.004 | 0.002 | 0.002 | 0.002 | 0.001 |
| 442 | -0.003 | 0.004 | 0.002 | 0.002 | 0.002 | 0.001 |
| 442.5 | -0.003 | 0.004 | 0.002 | 0.002 | 0.002 | 0.001 |
| 443 | -0.003 | 0.004 | 0.002 | 0.002 | 0.002 | 0.001 |
| 443.5 | -0.003 | 0.004 | 0.002 | 0.002 | 0.002 | 0.001 |
| 444 | -0.003 | 0.004 | 0.002 | 0.002 | 0.002 | 0.001 |
| 444.5 | -0.003 | 0.004 | 0.002 | 0.002 | 0.002 | 0.001 |
| 445 | -0.003 | 0.004 | 0.002 | 0.002 | 0.002 | 0.001 |
| 445.5 | -0.003 | 0.004 | 0.002 | 0.002 | 0.002 | 0.001 |
| 446 | -0.003 | 0.004 | 0.002 | 0.002 | 0.002 | 0.001 |
| 446.5 | -0.003 | 0.004 | 0.002 | 0.002 | 0.002 | 0.001 |
| 447 | -0.003 | 0.004 | 0.002 | 0.002 | 0.002 | 0.001 |
| 447.5 | -0.003 | 0.004 | 0.002 | 0.002 | 0.002 | 0.001 |
| 448 | -0.003 | 0.004 | 0.002 | 0.002 | 0.002 | 0.001 |
| 448.5 | -0.003 | 0.004 | 0.002 | 0.002 | 0.002 | 0.001 |
| 449 | -0.003 | 0.004 | 0.002 | 0.002 | 0.002 | 0.001 |
| 449.5 | -0.003 | 0.004 | 0.002 | 0.002 | 0.002 | 0.001 |
| 450 | -0.003 | 0.004 | 0.002 | 0.002 | 0.002 | 0.001 |
| 450.5 | -0.003 | 0.004 | 0.002 | 0.002 | 0.002 | 0.001 |
| 451 | -0.003 | 0.004 | 0.002 | 0.002 | 0.002 | 0.001 |
| 451.5 | -0.003 | 0.004 | 0.002 | 0.002 | 0.002 | 0.001 |
| 452 | -0.003 | 0.004 | 0.002 | 0.001 | 0.002 | 0.001 |
| 452.5 | -0.003 | 0.004 | 0.002 | 0.002 | 0.002 | 0.001 |
| 453 | -0.003 | 0.004 | 0.002 | 0.002 | 0.002 | 0.001 |
| 453.5 | -0.003 | 0.004 | 0.002 | 0.002 | 0.002 | 0.001 |
| 454 | -0.003 | 0.004 | 0.002 | 0.002 | 0.002 | 0.001 |
| 454.5 | -0.003 | 0.004 | 0.002 | 0.002 | 0.002 | 0.001 |
| 455 | -0.003 | 0.004 | 0.002 | 0.002 | 0.002 | 0.001 |
| 455.5 | -0.003 | 0.004 | 0.002 | 0.002 | 0.002 | 0.001 |
| 456 | -0.003 | 0.004 | 0.002 | 0.002 | 0.002 | 0.001 |
| 456.5 | -0.003 | 0.004 | 0.002 | 0.002 | 0.002 | 0.001 |
| 457 | -0.003 | 0.004 | 0.002 | 0.002 | 0.002 | 0.001 |
| 457.5 | -0.003 | 0.004 | 0.002 | 0.002 | 0.002 | 0.001 |
| 458 | -0.003 | 0.004 | 0.002 | 0.002 | 0.002 | 0.001 |
| 458.5 | -0.003 | 0.004 | 0.002 | 0.002 | 0.002 | 0.001 |
| 459 | -0.003 | 0.004 | 0.002 | 0.002 | 0.002 | 0.001 |
| 459.5 | -0.003 | 0.004 | 0.002 | 0.002 | 0.002 | 0.001 |
| 460 | -0.003 | 0.004 | 0.002 | 0.002 | 0.002 | 0.001 |
| 460.5 | -0.003 | 0.004 | 0.002 | 0.002 | 0.002 | 0.001 |
| 461 | -0.003 | 0.004 | 0.002 | 0.002 | 0.002 | 0.001 |
| 461.5 | -0.003 | 0.004 | 0.002 | 0.002 | 0.002 | 0.001 |
| 462 | -0.003 | 0.004 | 0.002 | 0.002 | 0.002 | 0.001 |
| 462.5 | -0.003 | 0.004 | 0.002 | 0.002 | 0.002 | 0.001 |
| 463 | -0.003 | 0.004 | 0.002 | 0.002 | 0.002 | 0.001 |
| 463.5 | -0.003 | 0.004 | 0.002 | 0.002 | 0.002 | 0.001 |
| 464 | -0.003 | 0.004 | 0.002 | 0.002 | 0.002 | 0.001 |
| 464.5 | -0.003 | 0.004 | 0.002 | 0.002 | 0.002 | 0.001 |
| 465 | -0.003 | 0.004 | 0.002 | 0.002 | 0.002 | 0.001 |
| 465.5 | -0.003 | 0.004 | 0.002 | 0.002 | 0.002 | 0.001 |
| 466 | -0.003 | 0.004 | 0.002 | 0.002 | 0.002 | 0.001 |
| 466.5 | -0.003 | 0.004 | 0.002 | 0.002 | 0.002 | 0.001 |
| 467 | -0.003 | 0.004 | 0.002 | 0.002 | 0.002 | 0.001 |
| 467.5 | -0.003 | 0.004 | 0.002 | 0.002 | 0.002 | 0.001 |
| 468 | -0.003 | 0.004 | 0.002 | 0.002 | 0.002 | 0.001 |
| 468.5 | -0.003 | 0.004 | 0.002 | 0.002 | 0.002 | 0.001 |
| 469 | -0.003 | 0.004 | 0.002 | 0.002 | 0.002 | 0.001 |
| 469.5 | -0.003 | 0.004 | 0.002 | 0.002 | 0.002 | 0.001 |
| 470 | -0.003 | 0.004 | 0.002 | 0.002 | 0.002 | 0.001 |
| 470.5 | -0.003 | 0.004 | 0.002 | 0.002 | 0.002 | 0.001 |
| 471 | -0.003 | 0.004 | 0.002 | 0.002 | 0.002 | 0.001 |
| 471.5 | -0.003 | 0.004 | 0.002 | 0.002 | 0.002 | 0.001 |
| 472 | -0.003 | 0.004 | 0.002 | 0.002 | 0.002 | 0.001 |
| 472.5 | -0.003 | 0.004 | 0.002 | 0.002 | 0.002 | 0.001 |
| 473 | -0.003 | 0.004 | 0.002 | 0.002 | 0.002 | 0.001 |
| 473.5 | -0.003 | 0.004 | 0.002 | 0.002 | 0.002 | 0.001 |
| 474 | -0.003 | 0.004 | 0.002 | 0.002 | 0.002 | 0.001 |
| 474.5 | -0.003 | 0.004 | 0.002 | 0.002 | 0.002 | 0.001 |
| 475 | -0.003 | 0.004 | 0.002 | 0.002 | 0.002 | 0.001 |
| 475.5 | -0.003 | 0.004 | 0.002 | 0.002 | 0.002 | 0.001 |
| 476 | -0.003 | 0.004 | 0.002 | 0.002 | 0.002 | 0.001 |
| 476.5 | -0.003 | 0.004 | 0.002 | 0.002 | 0.002 | 0.001 |
| 477 | -0.003 | 0.004 | 0.002 | 0.002 | 0.002 | 0.001 |
| 477.5 | -0.003 | 0.004 | 0.002 | 0.002 | 0.002 | 0.001 |
| 478 | -0.003 | 0.004 | 0.002 | 0.002 | 0.002 | 0.001 |
| 478.5 | -0.003 | 0.004 | 0.002 | 0.002 | 0.002 | 0.001 |
| 479 | -0.003 | 0.004 | 0.002 | 0.002 | 0.002 | 0.001 |
| 479.5 | -0.003 | 0.004 | 0.002 | 0.002 | 0.002 | 0.001 |
| 480 | -0.003 | 0.004 | 0.002 | 0.002 | 0.002 | 0.001 |
| 480.5 | -0.003 | 0.004 | 0.002 | 0.002 | 0.002 | 0.001 |
| 481 | -0.003 | 0.004 | 0.002 | 0.002 | 0.002 | 0.001 |
| 481.5 | -0.003 | 0.004 | 0.002 | 0.002 | 0.002 | 0.001 |
| 482 | -0.003 | 0.004 | 0.002 | 0.002 | 0.002 | 0.001 |
| 482.5 | -0.003 | 0.004 | 0.002 | 0.002 | 0.002 | 0.001 |
| 483 | -0.003 | 0.004 | 0.002 | 0.002 | 0.002 | 0.001 |
| 483.5 | -0.003 | 0.004 | 0.002 | 0.002 | 0.002 | 0.001 |
| 484 | -0.002 | 0.004 | 0.002 | 0.002 | 0.002 | 0.001 |
| 484.5 | -0.002 | 0.004 | 0.002 | 0.002 | 0.002 | 0.001 |
| 485 | -0.002 | 0.004 | 0.002 | 0.002 | 0.002 | 0.001 |
| 485.5 | -0.003 | 0.004 | 0.002 | 0.002 | 0.002 | 0.001 |
| 486 | -0.003 | 0.004 | 0.002 | 0.002 | 0.002 | 0.001 |
| 486.5 | -0.003 | 0.004 | 0.002 | 0.002 | 0.002 | 0.001 |
| 487 | -0.003 | 0.004 | 0.002 | 0.002 | 0.002 | 0.001 |
| 487.5 | -0.003 | 0.004 | 0.002 | 0.002 | 0.002 | 0.001 |
| 488 | -0.003 | 0.004 | 0.002 | 0.002 | 0.002 | 0.001 |
| 488.5 | -0.003 | 0.004 | 0.002 | 0.002 | 0.002 | 0.001 |
| 489 | -0.003 | 0.004 | 0.002 | 0.002 | 0.002 | 0.001 |
| 489.5 | -0.003 | 0.004 | 0.002 | 0.002 | 0.002 | 0.001 |
| 490 | -0.003 | 0.004 | 0.002 | 0.002 | 0.002 | 0.001 |
| 490.5 | -0.003 | 0.004 | 0.002 | 0.002 | 0.002 | 0.001 |
| 491 | -0.003 | 0.004 | 0.002 | 0.002 | 0.002 | 0.001 |
| 491.5 | -0.003 | 0.004 | 0.002 | 0.002 | 0.002 | 0.001 |
| 492 | -0.003 | 0.004 | 0.002 | 0.002 | 0.002 | 0.001 |
| 492.5 | -0.003 | 0.004 | 0.002 | 0.002 | 0.002 | 0.001 |
| 493 | -0.003 | 0.004 | 0.002 | 0.002 | 0.002 | 0.001 |
| 493.5 | -0.003 | 0.004 | 0.002 | 0.002 | 0.002 | 0.001 |
| 494 | -0.003 | 0.004 | 0.002 | 0.002 | 0.002 | 0.001 |
| 494.5 | -0.003 | 0.004 | 0.002 | 0.002 | 0.002 | 0.001 |
| 495 | -0.003 | 0.004 | 0.002 | 0.002 | 0.002 | 0.001 |
| 495.5 | -0.003 | 0.004 | 0.002 | 0.002 | 0.002 | 0.001 |
| 496 | -0.003 | 0.004 | 0.002 | 0.002 | 0.002 | 0.001 |
| 496.5 | -0.003 | 0.004 | 0.002 | 0.002 | 0.002 | 0.001 |
| 497 | -0.003 | 0.004 | 0.002 | 0.002 | 0.002 | 0.001 |
| 497.5 | -0.002 | 0.004 | 0.002 | 0.002 | 0.002 | 0.001 |
| 498 | -0.002 | 0.004 | 0.002 | 0.002 | 0.002 | 0.001 |
| 498.5 | -0.003 | 0.004 | 0.002 | 0.002 | 0.002 | 0.001 |
| 499 | -0.003 | 0.004 | 0.002 | 0.002 | 0.002 | 0.001 |
| 499.5 | -0.003 | 0.004 | 0.002 | 0.002 | 0.002 | 0.001 |
| 500 | -0.003 | 0.004 | 0.002 | 0.002 | 0.002 | 0.001 |
| 500.5 | -0.003 | 0.004 | 0.002 | 0.002 | 0.002 | 0.001 |
| 501 | -0.003 | 0.004 | 0.002 | 0.002 | 0.002 | 0.001 |
| 501.5 | -0.003 | 0.004 | 0.002 | 0.002 | 0.002 | 0.001 |
| 502 | -0.003 | 0.004 | 0.002 | 0.002 | 0.002 | 0.001 |
| 502.5 | -0.003 | 0.004 | 0.002 | 0.002 | 0.002 | 0.001 |
| 503 | -0.002 | 0.004 | 0.002 | 0.002 | 0.002 | 0.001 |
| 503.5 | -0.002 | 0.004 | 0.002 | 0.002 | 0.002 | 0.001 |
| 504 | -0.002 | 0.004 | 0.002 | 0.002 | 0.002 | 0.001 |
| 504.5 | -0.003 | 0.004 | 0.002 | 0.002 | 0.002 | 0.001 |
| 505 | -0.003 | 0.004 | 0.002 | 0.002 | 0.002 | 0.001 |
| 505.5 | -0.003 | 0.004 | 0.002 | 0.002 | 0.002 | 0.001 |
| 506 | -0.003 | 0.004 | 0.002 | 0.002 | 0.002 | 0.001 |
| 506.5 | -0.002 | 0.004 | 0.002 | 0.002 | 0.002 | 0.001 |
| 507 | -0.002 | 0.004 | 0.002 | 0.002 | 0.002 | 0.001 |
| 507.5 | -0.002 | 0.004 | 0.002 | 0.002 | 0.002 | 0.001 |
| 508 | -0.003 | 0.004 | 0.002 | 0.002 | 0.002 | 0.001 |
| 508.5 | -0.003 | 0.004 | 0.002 | 0.002 | 0.002 | 0.001 |
| 509 | -0.003 | 0.004 | 0.002 | 0.002 | 0.002 | 0.001 |
| 509.5 | -0.002 | 0.004 | 0.002 | 0.002 | 0.002 | 0.001 |
| 510 | -0.002 | 0.004 | 0.002 | 0.002 | 0.002 | 0.001 |
| 510.5 | -0.003 | 0.004 | 0.002 | 0.002 | 0.002 | 0.001 |
| 511 | -0.003 | 0.004 | 0.002 | 0.002 | 0.002 | 0.001 |
| 511.5 | -0.002 | 0.004 | 0.002 | 0.002 | 0.002 | 0.001 |
| 512 | -0.002 | 0.004 | 0.002 | 0.002 | 0.002 | 0.001 |
| 512.5 | -0.002 | 0.005 | 0.002 | 0.002 | 0.002 | 0.001 |
| 513 | -0.002 | 0.004 | 0.002 | 0.002 | 0.002 | 0.001 |
| 513.5 | -0.002 | 0.004 | 0.002 | 0.002 | 0.002 | 0.001 |
| 514 | -0.002 | 0.004 | 0.002 | 0.002 | 0.002 | 0.001 |
| 514.5 | -0.002 | 0.004 | 0.002 | 0.002 | 0.002 | 0.001 |
| 515 | -0.002 | 0.004 | 0.002 | 0.002 | 0.002 | 0.001 |
| 515.5 | -0.002 | 0.005 | 0.002 | 0.002 | 0.002 | 0.001 |
| 516 | -0.002 | 0.004 | 0.002 | 0.002 | 0.002 | 0.001 |
| 516.5 | -0.002 | 0.004 | 0.002 | 0.002 | 0.002 | 0.001 |
| 517 | -0.002 | 0.004 | 0.002 | 0.002 | 0.002 | 0.001 |
| 517.5 | -0.002 | 0.004 | 0.002 | 0.002 | 0.002 | 0.001 |
| 518 | -0.002 | 0.004 | 0.002 | 0.002 | 0.002 | 0.001 |
| 518.5 | -0.002 | 0.004 | 0.002 | 0.002 | 0.002 | 0.001 |
| 519 | -0.002 | 0.004 | 0.002 | 0.002 | 0.002 | 0.001 |
| 519.5 | -0.002 | 0.004 | 0.002 | 0.002 | 0.002 | 0.001 |
| 520 | -0.002 | 0.004 | 0.002 | 0.002 | 0.002 | 0.001 |
| 520.5 | -0.002 | 0.004 | 0.002 | 0.002 | 0.002 | 0.001 |
| 521 | -0.002 | 0.004 | 0.002 | 0.002 | 0.002 | 0.001 |
| 521.5 | -0.002 | 0.004 | 0.002 | 0.002 | 0.002 | 0.001 |
| 522 | -0.002 | 0.004 | 0.002 | 0.002 | 0.002 | 0.001 |
| 522.5 | -0.002 | 0.004 | 0.002 | 0.002 | 0.002 | 0.001 |
| 523 | -0.002 | 0.005 | 0.002 | 0.002 | 0.002 | 0.001 |
| 523.5 | -0.002 | 0.005 | 0.002 | 0.002 | 0.002 | 0.001 |
| 524 | -0.002 | 0.005 | 0.002 | 0.002 | 0.002 | 0.001 |
| 524.5 | -0.002 | 0.005 | 0.002 | 0.002 | 0.002 | 0.001 |
| 525 | -0.002 | 0.004 | 0.002 | 0.002 | 0.002 | 0.001 |
| 525.5 | -0.002 | 0.004 | 0.002 | 0.002 | 0.002 | 0.001 |
| 526 | -0.002 | 0.004 | 0.002 | 0.002 | 0.002 | 0.001 |
| 526.5 | -0.002 | 0.004 | 0.002 | 0.002 | 0.002 | 0.001 |
| 527 | -0.002 | 0.005 | 0.002 | 0.002 | 0.002 | 0.001 |
| 527.5 | -0.002 | 0.005 | 0.002 | 0.002 | 0.002 | 0.001 |
| 528 | -0.002 | 0.005 | 0.002 | 0.002 | 0.002 | 0.001 |
| 528.5 | -0.002 | 0.005 | 0.002 | 0.002 | 0.002 | 0.001 |
| 529 | -0.002 | 0.005 | 0.002 | 0.002 | 0.002 | 0.001 |
| 529.5 | -0.002 | 0.005 | 0.002 | 0.002 | 0.002 | 0.001 |
| 530 | -0.002 | 0.005 | 0.002 | 0.002 | 0.002 | 0.001 |
| 530.5 | -0.002 | 0.005 | 0.002 | 0.002 | 0.002 | 0.001 |
| 531 | -0.002 | 0.005 | 0.002 | 0.002 | 0.002 | 0.001 |
| 531.5 | -0.002 | 0.005 | 0.002 | 0.002 | 0.002 | 0.001 |
| 532 | -0.002 | 0.005 | 0.002 | 0.002 | 0.002 | 0.001 |
| 532.5 | -0.002 | 0.005 | 0.002 | 0.002 | 0.002 | 0.001 |
| 533 | -0.002 | 0.005 | 0.002 | 0.002 | 0.002 | 0.001 |
| 533.5 | -0.002 | 0.005 | 0.002 | 0.002 | 0.002 | 0.001 |
| 534 | -0.002 | 0.005 | 0.002 | 0.002 | 0.002 | 0.001 |
| 534.5 | -0.002 | 0.005 | 0.002 | 0.002 | 0.002 | 0.001 |
| 535 | -0.002 | 0.005 | 0.002 | 0.002 | 0.002 | 0.001 |
| 535.5 | -0.002 | 0.005 | 0.002 | 0.002 | 0.002 | 0.001 |
| 536 | -0.002 | 0.005 | 0.002 | 0.002 | 0.002 | 0.001 |
| 536.5 | -0.002 | 0.005 | 0.002 | 0.002 | 0.002 | 0.001 |
| 537 | -0.002 | 0.005 | 0.002 | 0.002 | 0.002 | 0.001 |
| 537.5 | -0.002 | 0.005 | 0.002 | 0.002 | 0.002 | 0.001 |
| 538 | -0.002 | 0.005 | 0.002 | 0.002 | 0.002 | 0.001 |
| 538.5 | -0.002 | 0.005 | 0.002 | 0.002 | 0.002 | 0.001 |
| 539 | -0.002 | 0.005 | 0.002 | 0.002 | 0.002 | 0.001 |
| 539.5 | -0.002 | 0.005 | 0.003 | 0.002 | 0.002 | 0.001 |
| 540 | -0.002 | 0.005 | 0.003 | 0.003 | 0.003 | 0.002 |
| 540.5 | -0.002 | 0.005 | 0.003 | 0.002 | 0.002 | 0.001 |
| 541 | -0.002 | 0.005 | 0.002 | 0.002 | 0.002 | 0.001 |
| 541.5 | -0.002 | 0.005 | 0.002 | 0.002 | 0.002 | 0.001 |
| 542 | -0.002 | 0.005 | 0.002 | 0.002 | 0.002 | 0.001 |
| 542.5 | -0.002 | 0.005 | 0.002 | 0.002 | 0.002 | 0.001 |
| 543 | -0.002 | 0.005 | 0.002 | 0.002 | 0.002 | 0.001 |
| 543.5 | -0.002 | 0.005 | 0.002 | 0.002 | 0.002 | 0.001 |
| 544 | -0.002 | 0.005 | 0.002 | 0.002 | 0.002 | 0.001 |
| 544.5 | -0.002 | 0.005 | 0.002 | 0.002 | 0.002 | 0.001 |
| 545 | -0.002 | 0.005 | 0.002 | 0.002 | 0.002 | 0.001 |
| 545.5 | -0.002 | 0.005 | 0.002 | 0.002 | 0.002 | 0.001 |
| 546 | -0.002 | 0.005 | 0.002 | 0.002 | 0.002 | 0.001 |
| 546.5 | -0.002 | 0.005 | 0.002 | 0.002 | 0.002 | 0.001 |
| 547 | -0.002 | 0.005 | 0.002 | 0.002 | 0.002 | 0.001 |
| 547.5 | -0.002 | 0.005 | 0.002 | 0.002 | 0.002 | 0.001 |
| 548 | -0.002 | 0.005 | 0.002 | 0.002 | 0.002 | 0.001 |
| 548.5 | -0.002 | 0.005 | 0.002 | 0.002 | 0.002 | 0.001 |
| 549 | -0.002 | 0.005 | 0.002 | 0.002 | 0.002 | 0.001 |
| 549.5 | -0.002 | 0.005 | 0.002 | 0.002 | 0.002 | 0.001 |
| 550 | -0.002 | 0.005 | 0.002 | 0.002 | 0.002 | 0.001 |
| 550.5 | -0.002 | 0.005 | 0.002 | 0.002 | 0.002 | 0.001 |
| 551 | -0.002 | 0.005 | 0.002 | 0.002 | 0.002 | 0.001 |
| 551.5 | -0.002 | 0.005 | 0.002 | 0.002 | 0.002 | 0.001 |
| 552 | -0.002 | 0.005 | 0.002 | 0.002 | 0.002 | 0.001 |
| 552.5 | -0.002 | 0.005 | 0.002 | 0.002 | 0.002 | 0.001 |
| 553 | -0.002 | 0.005 | 0.002 | 0.002 | 0.002 | 0.001 |
| 553.5 | -0.002 | 0.005 | 0.002 | 0.002 | 0.002 | 0.001 |
| 554 | -0.002 | 0.005 | 0.002 | 0.002 | 0.002 | 0.001 |
| 554.5 | -0.002 | 0.005 | 0.002 | 0.002 | 0.002 | 0.001 |
| 555 | -0.002 | 0.005 | 0.002 | 0.002 | 0.002 | 0.001 |
| 555.5 | -0.002 | 0.005 | 0.002 | 0.002 | 0.002 | 0.001 |
| 556 | -0.002 | 0.005 | 0.002 | 0.002 | 0.002 | 0.001 |
| 556.5 | -0.002 | 0.005 | 0.002 | 0.002 | 0.002 | 0.001 |
| 557 | -0.002 | 0.005 | 0.002 | 0.002 | 0.002 | 0.001 |
| 557.5 | -0.002 | 0.005 | 0.002 | 0.002 | 0.002 | 0.001 |
| 558 | -0.002 | 0.005 | 0.002 | 0.002 | 0.002 | 0.001 |
| 558.5 | -0.002 | 0.005 | 0.002 | 0.002 | 0.002 | 0.001 |
| 559 | -0.002 | 0.005 | 0.002 | 0.002 | 0.002 | 0.001 |
| 559.5 | -0.002 | 0.005 | 0.002 | 0.002 | 0.002 | 0.001 |
| 560 | -0.002 | 0.005 | 0.002 | 0.002 | 0.002 | 0.001 |
| 560.5 | -0.002 | 0.005 | 0.002 | 0.002 | 0.002 | 0.001 |
| 561 | -0.002 | 0.005 | 0.002 | 0.002 | 0.002 | 0.001 |
| 561.5 | -0.002 | 0.005 | 0.002 | 0.002 | 0.002 | 0.001 |
| 562 | -0.002 | 0.005 | 0.002 | 0.002 | 0.002 | 0.001 |
| 562.5 | -0.002 | 0.005 | 0.002 | 0.002 | 0.002 | 0.001 |
| 563 | -0.002 | 0.005 | 0.002 | 0.002 | 0.002 | 0.001 |
| 563.5 | -0.002 | 0.005 | 0.002 | 0.002 | 0.002 | 0.001 |
| 564 | -0.002 | 0.005 | 0.002 | 0.002 | 0.002 | 0.001 |
| 564.5 | -0.002 | 0.005 | 0.002 | 0.002 | 0.002 | 0.001 |
| 565 | -0.002 | 0.005 | 0.002 | 0.002 | 0.002 | 0.001 |
| 565.5 | -0.002 | 0.005 | 0.002 | 0.002 | 0.002 | 0.001 |
| 566 | -0.002 | 0.005 | 0.002 | 0.002 | 0.002 | 0.001 |
| 566.5 | -0.002 | 0.005 | 0.002 | 0.002 | 0.002 | 0.001 |
| 567 | -0.002 | 0.005 | 0.002 | 0.002 | 0.002 | 0.001 |
| 567.5 | -0.002 | 0.005 | 0.002 | 0.002 | 0.002 | 0.001 |
| 568 | -0.002 | 0.005 | 0.002 | 0.002 | 0.002 | 0.001 |
| 568.5 | -0.003 | 0.004 | 0.002 | 0.001 | 0.001 | 0 |
| 569 | -0.003 | 0.004 | 0.002 | 0.001 | 0.001 | 0 |
| 569.5 | -0.002 | 0.004 | 0.002 | 0.001 | 0.002 | 0.001 |
| 570 | -0.002 | 0.005 | 0.002 | 0.001 | 0.002 | 0.001 |
| 570.5 | -0.002 | 0.005 | 0.002 | 0.002 | 0.002 | 0.001 |
| 571 | -0.002 | 0.005 | 0.002 | 0.002 | 0.002 | 0.001 |
| 571.5 | -0.002 | 0.005 | 0.002 | 0.002 | 0.002 | 0.001 |
| 572 | -0.002 | 0.005 | 0.002 | 0.002 | 0.002 | 0.001 |
| 572.5 | -0.002 | 0.005 | 0.002 | 0.002 | 0.002 | 0.001 |
| 573 | -0.002 | 0.005 | 0.002 | 0.002 | 0.002 | 0.001 |
| 573.5 | -0.002 | 0.005 | 0.002 | 0.002 | 0.002 | 0.001 |
| 574 | -0.002 | 0.005 | 0.002 | 0.002 | 0.002 | 0.001 |
| 574.5 | -0.002 | 0.005 | 0.002 | 0.002 | 0.002 | 0.001 |
| 575 | -0.002 | 0.005 | 0.002 | 0.002 | 0.002 | 0.001 |
| 575.5 | -0.002 | 0.005 | 0.002 | 0.002 | 0.002 | 0.001 |
| 576 | -0.002 | 0.005 | 0.002 | 0.002 | 0.002 | 0.001 |
| 576.5 | -0.002 | 0.005 | 0.002 | 0.002 | 0.002 | 0.001 |
| 577 | -0.002 | 0.005 | 0.002 | 0.002 | 0.002 | 0.001 |
| 577.5 | -0.002 | 0.005 | 0.002 | 0.002 | 0.002 | 0.001 |
| 578 | -0.002 | 0.005 | 0.002 | 0.002 | 0.002 | 0.001 |
| 578.5 | -0.002 | 0.005 | 0.002 | 0.002 | 0.002 | 0.001 |
| 579 | -0.002 | 0.005 | 0.002 | 0.002 | 0.002 | 0.001 |
| 579.5 | -0.002 | 0.005 | 0.002 | 0.002 | 0.002 | 0.001 |
| 580 | -0.002 | 0.005 | 0.002 | 0.002 | 0.002 | 0.001 |
| 580.5 | -0.002 | 0.005 | 0.002 | 0.002 | 0.002 | 0.001 |
| 581 | -0.002 | 0.005 | 0.002 | 0.002 | 0.002 | 0.001 |
| 581.5 | -0.002 | 0.005 | 0.002 | 0.002 | 0.002 | 0.001 |
| 582 | -0.002 | 0.005 | 0.002 | 0.002 | 0.002 | 0.001 |
| 582.5 | -0.002 | 0.005 | 0.002 | 0.002 | 0.002 | 0.001 |
| 583 | -0.002 | 0.005 | 0.002 | 0.002 | 0.002 | 0.001 |
| 583.5 | -0.002 | 0.005 | 0.002 | 0.002 | 0.002 | 0.001 |
| 584 | -0.002 | 0.005 | 0.002 | 0.002 | 0.002 | 0.001 |
| 584.5 | -0.002 | 0.005 | 0.002 | 0.002 | 0.002 | 0.001 |
| 585 | -0.002 | 0.005 | 0.002 | 0.002 | 0.002 | 0.001 |
| 585.5 | -0.002 | 0.005 | 0.002 | 0.002 | 0.002 | 0.001 |
| 586 | -0.002 | 0.005 | 0.002 | 0.002 | 0.002 | 0.002 |
| 586.5 | -0.002 | 0.005 | 0.002 | 0.002 | 0.002 | 0.002 |
| 587 | -0.002 | 0.005 | 0.003 | 0.002 | 0.002 | 0.002 |
| 587.5 | -0.002 | 0.005 | 0.003 | 0.002 | 0.002 | 0.002 |
| 588 | -0.002 | 0.005 | 0.003 | 0.002 | 0.002 | 0.002 |
| 588.5 | -0.002 | 0.005 | 0.002 | 0.002 | 0.002 | 0.002 |
| 589 | -0.002 | 0.005 | 0.002 | 0.002 | 0.002 | 0.002 |
| 589.5 | -0.002 | 0.005 | 0.002 | 0.002 | 0.002 | 0.002 |
| 590 | -0.002 | 0.005 | 0.003 | 0.002 | 0.002 | 0.002 |
| 590.5 | -0.002 | 0.005 | 0.003 | 0.002 | 0.002 | 0.002 |
| 591 | -0.002 | 0.005 | 0.003 | 0.002 | 0.002 | 0.002 |
| 591.5 | -0.002 | 0.005 | 0.003 | 0.002 | 0.002 | 0.002 |
| 592 | -0.002 | 0.005 | 0.003 | 0.002 | 0.002 | 0.002 |
| 592.5 | -0.002 | 0.005 | 0.003 | 0.002 | 0.002 | 0.002 |
| 593 | -0.002 | 0.005 | 0.003 | 0.002 | 0.002 | 0.002 |
| 593.5 | -0.002 | 0.005 | 0.003 | 0.002 | 0.002 | 0.002 |
| 594 | -0.002 | 0.005 | 0.003 | 0.002 | 0.002 | 0.002 |
| 594.5 | -0.002 | 0.005 | 0.003 | 0.002 | 0.002 | 0.002 |
| 595 | -0.002 | 0.005 | 0.003 | 0.002 | 0.002 | 0.002 |
| 595.5 | -0.002 | 0.005 | 0.003 | 0.002 | 0.002 | 0.002 |
| 596 | -0.002 | 0.005 | 0.003 | 0.002 | 0.002 | 0.002 |
| 596.5 | -0.002 | 0.005 | 0.003 | 0.002 | 0.002 | 0.002 |
| 597 | -0.002 | 0.005 | 0.003 | 0.002 | 0.002 | 0.002 |
| 597.5 | -0.002 | 0.005 | 0.003 | 0.002 | 0.002 | 0.002 |
| 598 | -0.002 | 0.005 | 0.003 | 0.002 | 0.002 | 0.002 |
| 598.5 | -0.002 | 0.005 | 0.003 | 0.002 | 0.002 | 0.002 |
| 599 | -0.001 | 0.005 | 0.003 | 0.002 | 0.002 | 0.002 |
| 599.5 | -0.001 | 0.005 | 0.003 | 0.002 | 0.002 | 0.002 |
| 600 | -0.001 | 0.005 | 0.003 | 0.002 | 0.002 | 0.002 |
| 600.5 | -0.001 | 0.005 | 0.003 | 0.002 | 0.002 | 0.002 |
| 601 | -0.002 | 0.005 | 0.003 | 0.002 | 0.002 | 0.002 |
| 601.5 | -0.002 | 0.005 | 0.003 | 0.002 | 0.002 | 0.002 |
| 602 | -0.002 | 0.005 | 0.003 | 0.002 | 0.002 | 0.002 |
| 602.5 | -0.002 | 0.005 | 0.003 | 0.002 | 0.002 | 0.002 |
| 603 | -0.001 | 0.005 | 0.003 | 0.002 | 0.002 | 0.002 |
| 603.5 | -0.002 | 0.005 | 0.003 | 0.002 | 0.002 | 0.002 |
| 604 | -0.002 | 0.005 | 0.003 | 0.002 | 0.002 | 0.002 |
| 604.5 | -0.001 | 0.005 | 0.003 | 0.002 | 0.002 | 0.002 |
| 605 | -0.001 | 0.005 | 0.003 | 0.002 | 0.002 | 0.002 |
| 605.5 | -0.001 | 0.005 | 0.003 | 0.002 | 0.002 | 0.002 |
| 606 | -0.001 | 0.005 | 0.003 | 0.002 | 0.002 | 0.002 |
| 606.5 | -0.001 | 0.005 | 0.003 | 0.002 | 0.002 | 0.002 |
| 607 | -0.001 | 0.005 | 0.003 | 0.002 | 0.002 | 0.002 |
| 607.5 | -0.001 | 0.005 | 0.003 | 0.002 | 0.002 | 0.002 |
| 608 | -0.001 | 0.005 | 0.003 | 0.002 | 0.002 | 0.002 |
| 608.5 | -0.001 | 0.005 | 0.003 | 0.002 | 0.002 | 0.002 |
| 609 | -0.001 | 0.005 | 0.003 | 0.002 | 0.002 | 0.002 |
| 609.5 | -0.001 | 0.005 | 0.003 | 0.002 | 0.002 | 0.002 |
| 610 | -0.001 | 0.005 | 0.003 | 0.002 | 0.002 | 0.002 |
| 610.5 | -0.001 | 0.005 | 0.003 | 0.002 | 0.002 | 0.002 |
| 611 | -0.001 | 0.005 | 0.003 | 0.002 | 0.002 | 0.002 |
| 611.5 | -0.001 | 0.005 | 0.003 | 0.002 | 0.002 | 0.002 |
| 612 | -0.001 | 0.005 | 0.003 | 0.002 | 0.002 | 0.002 |
| 612.5 | -0.001 | 0.005 | 0.003 | 0.002 | 0.002 | 0.002 |
| 613 | -0.001 | 0.005 | 0.003 | 0.002 | 0.002 | 0.002 |
| 613.5 | -0.001 | 0.005 | 0.003 | 0.002 | 0.002 | 0.002 |
| 614 | -0.001 | 0.005 | 0.003 | 0.002 | 0.002 | 0.002 |
| 614.5 | -0.001 | 0.005 | 0.003 | 0.002 | 0.002 | 0.002 |
| 615 | -0.001 | 0.005 | 0.003 | 0.002 | 0.002 | 0.002 |
| 615.5 | -0.001 | 0.005 | 0.003 | 0.002 | 0.002 | 0.002 |
| 616 | -0.001 | 0.005 | 0.003 | 0.002 | 0.002 | 0.002 |
| 616.5 | -0.001 | 0.005 | 0.003 | 0.002 | 0.002 | 0.002 |
| 617 | -0.001 | 0.005 | 0.003 | 0.002 | 0.002 | 0.002 |
| 617.5 | -0.001 | 0.005 | 0.003 | 0.002 | 0.002 | 0.002 |
| 618 | -0.001 | 0.005 | 0.003 | 0.002 | 0.002 | 0.002 |
| 618.5 | -0.001 | 0.005 | 0.003 | 0.002 | 0.002 | 0.002 |
| 619 | -0.001 | 0.005 | 0.003 | 0.002 | 0.002 | 0.002 |
| 619.5 | -0.001 | 0.005 | 0.003 | 0.002 | 0.002 | 0.002 |
| 620 | -0.001 | 0.005 | 0.003 | 0.002 | 0.002 | 0.002 |
| 620.5 | -0.001 | 0.005 | 0.003 | 0.002 | 0.002 | 0.002 |
| 621 | -0.001 | 0.005 | 0.003 | 0.002 | 0.002 | 0.002 |
| 621.5 | -0.001 | 0.005 | 0.003 | 0.002 | 0.002 | 0.002 |
| 622 | -0.001 | 0.005 | 0.003 | 0.002 | 0.002 | 0.002 |
| 622.5 | -0.001 | 0.005 | 0.003 | 0.002 | 0.002 | 0.002 |
| 623 | -0.001 | 0.005 | 0.003 | 0.002 | 0.002 | 0.002 |
| 623.5 | -0.001 | 0.005 | 0.003 | 0.002 | 0.002 | 0.002 |
| 624 | -0.001 | 0.006 | 0.003 | 0.002 | 0.002 | 0.002 |
| 624.5 | -0.001 | 0.006 | 0.003 | 0.002 | 0.002 | 0.002 |
| 625 | -0.001 | 0.005 | 0.003 | 0.002 | 0.002 | 0.002 |
| 625.5 | -0.001 | 0.005 | 0.003 | 0.002 | 0.002 | 0.002 |
| 626 | -0.001 | 0.006 | 0.003 | 0.002 | 0.003 | 0.002 |
| 626.5 | -0.001 | 0.006 | 0.003 | 0.002 | 0.003 | 0.002 |
| 627 | -0.001 | 0.006 | 0.003 | 0.002 | 0.003 | 0.002 |
| 627.5 | -0.001 | 0.006 | 0.003 | 0.002 | 0.003 | 0.002 |
| 628 | -0.001 | 0.006 | 0.003 | 0.002 | 0.003 | 0.002 |
| 628.5 | -0.001 | 0.006 | 0.003 | 0.002 | 0.003 | 0.002 |
| 629 | -0.001 | 0.006 | 0.003 | 0.003 | 0.003 | 0.003 |
| 629.5 | -0.001 | 0.006 | 0.003 | 0.003 | 0.003 | 0.003 |
| 630 | -0.001 | 0.006 | 0.003 | 0.003 | 0.003 | 0.003 |
| 630.5 | -0.001 | 0.006 | 0.003 | 0.003 | 0.003 | 0.003 |
| 631 | -0.001 | 0.006 | 0.003 | 0.003 | 0.003 | 0.003 |
| 631.5 | -0.001 | 0.006 | 0.004 | 0.003 | 0.003 | 0.003 |
| 632 | -0.001 | 0.006 | 0.004 | 0.003 | 0.003 | 0.003 |
| 632.5 | -0.001 | 0.006 | 0.004 | 0.003 | 0.003 | 0.003 |
| 633 | -0.001 | 0.006 | 0.004 | 0.003 | 0.003 | 0.003 |
| 633.5 | -0.001 | 0.006 | 0.004 | 0.003 | 0.003 | 0.003 |
| 634 | -0.001 | 0.006 | 0.004 | 0.003 | 0.003 | 0.003 |
| 634.5 | -0.001 | 0.006 | 0.004 | 0.003 | 0.003 | 0.003 |
| 635 | -0.001 | 0.006 | 0.004 | 0.003 | 0.003 | 0.003 |
| 635.5 | -0.001 | 0.006 | 0.004 | 0.003 | 0.003 | 0.003 |
| 636 | -0.001 | 0.006 | 0.004 | 0.003 | 0.003 | 0.003 |
| 636.5 | -0.001 | 0.006 | 0.004 | 0.003 | 0.003 | 0.003 |
| 637 | -0.001 | 0.006 | 0.004 | 0.003 | 0.003 | 0.003 |
| 637.5 | -0.001 | 0.006 | 0.004 | 0.003 | 0.003 | 0.003 |
| 638 | 0 | 0.006 | 0.004 | 0.003 | 0.003 | 0.003 |
| 638.5 | 0 | 0.006 | 0.004 | 0.003 | 0.003 | 0.003 |
| 639 | -0.001 | 0.006 | 0.004 | 0.003 | 0.003 | 0.003 |
| 639.5 | -0.001 | 0.006 | 0.004 | 0.003 | 0.003 | 0.003 |
| 640 | 0 | 0.006 | 0.004 | 0.003 | 0.004 | 0.003 |
| 640.5 | 0 | 0.006 | 0.004 | 0.003 | 0.004 | 0.003 |
| 641 | 0 | 0.006 | 0.004 | 0.003 | 0.004 | 0.003 |
| 641.5 | 0 | 0.007 | 0.004 | 0.003 | 0.004 | 0.003 |
| 642 | 0 | 0.007 | 0.004 | 0.003 | 0.004 | 0.003 |
| 642.5 | 0 | 0.007 | 0.004 | 0.003 | 0.004 | 0.003 |
| 643 | 0 | 0.007 | 0.004 | 0.003 | 0.004 | 0.003 |
| 643.5 | 0 | 0.007 | 0.004 | 0.003 | 0.004 | 0.003 |
| 644 | 0 | 0.007 | 0.004 | 0.003 | 0.004 | 0.003 |
| 644.5 | 0 | 0.007 | 0.004 | 0.003 | 0.004 | 0.004 |
| 645 | 0 | 0.007 | 0.004 | 0.003 | 0.004 | 0.004 |
| 645.5 | 0 | 0.007 | 0.004 | 0.003 | 0.004 | 0.003 |
| 646 | 0 | 0.007 | 0.004 | 0.003 | 0.004 | 0.003 |
| 646.5 | 0 | 0.007 | 0.004 | 0.003 | 0.004 | 0.003 |
| 647 | 0 | 0.007 | 0.004 | 0.003 | 0.004 | 0.004 |
| 647.5 | 0 | 0.007 | 0.004 | 0.003 | 0.004 | 0.004 |
| 648 | 0 | 0.007 | 0.004 | 0.003 | 0.004 | 0.004 |
| 648.5 | 0 | 0.007 | 0.004 | 0.003 | 0.004 | 0.003 |
| 649 | 0 | 0.007 | 0.004 | 0.003 | 0.004 | 0.003 |
| 649.5 | 0 | 0.007 | 0.004 | 0.003 | 0.004 | 0.004 |
| 650 | 0 | 0.007 | 0.004 | 0.003 | 0.004 | 0.004 |
| 650.5 | 0 | 0.007 | 0.004 | 0.003 | 0.004 | 0.004 |
| 651 | 0 | 0.007 | 0.004 | 0.004 | 0.004 | 0.004 |
| 651.5 | 0 | 0.007 | 0.004 | 0.004 | 0.004 | 0.004 |
| 652 | 0 | 0.007 | 0.004 | 0.004 | 0.004 | 0.004 |
| 652.5 | 0 | 0.007 | 0.004 | 0.004 | 0.004 | 0.004 |
| 653 | 0 | 0.007 | 0.004 | 0.004 | 0.004 | 0.004 |
| 653.5 | 0 | 0.007 | 0.004 | 0.003 | 0.004 | 0.004 |
| 654 | 0 | 0.007 | 0.004 | 0.003 | 0.004 | 0.004 |
| 654.5 | 0 | 0.007 | 0.004 | 0.004 | 0.004 | 0.004 |
| 655 | 0 | 0.007 | 0.004 | 0.004 | 0.004 | 0.004 |
| 655.5 | 0 | 0.007 | 0.004 | 0.004 | 0.004 | 0.004 |
| 656 | 0 | 0.007 | 0.004 | 0.004 | 0.004 | 0.004 |
| 656.5 | 0 | 0.007 | 0.004 | 0.004 | 0.004 | 0.004 |
| 657 | 0 | 0.007 | 0.004 | 0.004 | 0.004 | 0.004 |
| 657.5 | 0 | 0.007 | 0.004 | 0.004 | 0.004 | 0.004 |
| 658 | 0 | 0.007 | 0.004 | 0.004 | 0.004 | 0.004 |
| 658.5 | 0 | 0.007 | 0.004 | 0.004 | 0.004 | 0.004 |
| 659 | 0 | 0.007 | 0.004 | 0.003 | 0.004 | 0.003 |
| 659.5 | 0 | 0.007 | 0.004 | 0.003 | 0.004 | 0.003 |
| 660 | 0 | 0.007 | 0.004 | 0.003 | 0.004 | 0.003 |
| 660.5 | 0 | 0.007 | 0.004 | 0.003 | 0.004 | 0.003 |
| 661 | 0 | 0.007 | 0.004 | 0.003 | 0.004 | 0.003 |
| 661.5 | 0 | 0.007 | 0.004 | 0.003 | 0.004 | 0.003 |
| 662 | 0 | 0.007 | 0.004 | 0.003 | 0.004 | 0.003 |
| 662.5 | 0 | 0.007 | 0.004 | 0.003 | 0.004 | 0.003 |
| 663 | 0 | 0.007 | 0.004 | 0.003 | 0.004 | 0.003 |
| 663.5 | 0 | 0.007 | 0.004 | 0.003 | 0.004 | 0.003 |
| 664 | 0 | 0.007 | 0.004 | 0.003 | 0.004 | 0.003 |
| 664.5 | 0 | 0.007 | 0.004 | 0.003 | 0.004 | 0.003 |
| 665 | -0.001 | 0.007 | 0.004 | 0.003 | 0.004 | 0.003 |
| 665.5 | -0.001 | 0.007 | 0.004 | 0.003 | 0.004 | 0.003 |
| 666 | -0.001 | 0.007 | 0.004 | 0.003 | 0.004 | 0.003 |
| 666.5 | -0.001 | 0.007 | 0.004 | 0.003 | 0.004 | 0.003 |
| 667 | -0.001 | 0.007 | 0.004 | 0.003 | 0.004 | 0.003 |
| 667.5 | -0.001 | 0.006 | 0.004 | 0.003 | 0.004 | 0.003 |
| 668 | -0.001 | 0.006 | 0.004 | 0.003 | 0.004 | 0.003 |
| 668.5 | -0.001 | 0.006 | 0.004 | 0.003 | 0.004 | 0.003 |
| 669 | -0.001 | 0.006 | 0.004 | 0.003 | 0.004 | 0.003 |
| 669.5 | -0.001 | 0.006 | 0.004 | 0.003 | 0.004 | 0.003 |
| 670 | -0.001 | 0.006 | 0.004 | 0.003 | 0.003 | 0.003 |
| 670.5 | -0.001 | 0.006 | 0.004 | 0.003 | 0.003 | 0.003 |
| 671 | -0.001 | 0.006 | 0.004 | 0.003 | 0.003 | 0.003 |
| 671.5 | -0.001 | 0.006 | 0.004 | 0.003 | 0.003 | 0.003 |
| 672 | -0.001 | 0.006 | 0.004 | 0.003 | 0.003 | 0.003 |
| 672.5 | -0.001 | 0.006 | 0.004 | 0.003 | 0.003 | 0.003 |
| 673 | -0.001 | 0.006 | 0.004 | 0.003 | 0.003 | 0.003 |
| 673.5 | -0.001 | 0.006 | 0.004 | 0.003 | 0.003 | 0.003 |
| 674 | -0.001 | 0.006 | 0.003 | 0.003 | 0.003 | 0.003 |
| 674.5 | -0.001 | 0.006 | 0.003 | 0.003 | 0.003 | 0.003 |
| 675 | -0.001 | 0.006 | 0.003 | 0.003 | 0.003 | 0.002 |
| 675.5 | -0.001 | 0.006 | 0.003 | 0.003 | 0.003 | 0.002 |
| 676 | -0.001 | 0.006 | 0.003 | 0.003 | 0.003 | 0.002 |
| 676.5 | -0.001 | 0.006 | 0.003 | 0.003 | 0.003 | 0.002 |
| 677 | -0.001 | 0.006 | 0.003 | 0.003 | 0.003 | 0.002 |
| 677.5 | -0.001 | 0.006 | 0.003 | 0.003 | 0.003 | 0.002 |
| 678 | -0.001 | 0.006 | 0.003 | 0.003 | 0.003 | 0.002 |
| 678.5 | -0.001 | 0.006 | 0.003 | 0.003 | 0.003 | 0.002 |
| 679 | -0.001 | 0.006 | 0.003 | 0.003 | 0.003 | 0.002 |
| 679.5 | -0.001 | 0.006 | 0.003 | 0.003 | 0.003 | 0.002 |
| 680 | -0.001 | 0.006 | 0.003 | 0.003 | 0.003 | 0.002 |
| 680.5 | -0.001 | 0.006 | 0.003 | 0.003 | 0.003 | 0.002 |
| 681 | -0.001 | 0.006 | 0.003 | 0.003 | 0.003 | 0.002 |
| 681.5 | -0.001 | 0.006 | 0.003 | 0.003 | 0.003 | 0.002 |
| 682 | -0.002 | 0.006 | 0.003 | 0.003 | 0.003 | 0.002 |
| 682.5 | -0.002 | 0.006 | 0.003 | 0.003 | 0.003 | 0.002 |
| 683 | -0.002 | 0.006 | 0.003 | 0.002 | 0.003 | 0.002 |
| 683.5 | -0.002 | 0.006 | 0.003 | 0.002 | 0.003 | 0.002 |
| 684 | -0.002 | 0.006 | 0.003 | 0.002 | 0.003 | 0.002 |
| 684.5 | -0.002 | 0.006 | 0.003 | 0.002 | 0.003 | 0.002 |
| 685 | -0.002 | 0.006 | 0.003 | 0.002 | 0.003 | 0.002 |
| 685.5 | -0.002 | 0.006 | 0.003 | 0.002 | 0.003 | 0.002 |
| 686 | -0.002 | 0.006 | 0.003 | 0.002 | 0.003 | 0.002 |
| 686.5 | -0.002 | 0.006 | 0.003 | 0.002 | 0.003 | 0.002 |
| 687 | -0.002 | 0.006 | 0.003 | 0.002 | 0.003 | 0.002 |
| 687.5 | -0.002 | 0.006 | 0.003 | 0.002 | 0.003 | 0.002 |
| 688 | -0.002 | 0.006 | 0.003 | 0.002 | 0.003 | 0.002 |
| 688.5 | -0.002 | 0.006 | 0.003 | 0.002 | 0.003 | 0.002 |
| 689 | -0.002 | 0.006 | 0.003 | 0.002 | 0.003 | 0.002 |
| 689.5 | -0.002 | 0.006 | 0.003 | 0.002 | 0.003 | 0.002 |
| 690 | -0.002 | 0.006 | 0.003 | 0.002 | 0.003 | 0.002 |
| 690.5 | -0.002 | 0.006 | 0.003 | 0.002 | 0.003 | 0.002 |
| 691 | -0.002 | 0.006 | 0.003 | 0.002 | 0.003 | 0.002 |
| 691.5 | -0.002 | 0.006 | 0.003 | 0.002 | 0.002 | 0.002 |
| 692 | -0.002 | 0.006 | 0.003 | 0.002 | 0.002 | 0.002 |
| 692.5 | -0.002 | 0.006 | 0.003 | 0.002 | 0.002 | 0.002 |
| 693 | -0.002 | 0.006 | 0.003 | 0.002 | 0.002 | 0.002 |
| 693.5 | -0.002 | 0.006 | 0.003 | 0.002 | 0.002 | 0.002 |
| 694 | -0.002 | 0.006 | 0.003 | 0.002 | 0.002 | 0.002 |
| 694.5 | -0.002 | 0.006 | 0.002 | 0.002 | 0.002 | 0.001 |
| 695 | -0.002 | 0.006 | 0.002 | 0.002 | 0.002 | 0.001 |
| 695.5 | -0.002 | 0.006 | 0.002 | 0.002 | 0.002 | 0.001 |
| 696 | -0.002 | 0.006 | 0.002 | 0.002 | 0.002 | 0.002 |
| 696.5 | -0.002 | 0.006 | 0.002 | 0.002 | 0.002 | 0.001 |
| 697 | -0.002 | 0.006 | 0.002 | 0.002 | 0.002 | 0.001 |
| 697.5 | -0.002 | 0.006 | 0.002 | 0.002 | 0.002 | 0.001 |
| 698 | -0.002 | 0.006 | 0.002 | 0.002 | 0.002 | 0.001 |
| 698.5 | -0.002 | 0.006 | 0.002 | 0.002 | 0.002 | 0.001 |
| 699 | -0.002 | 0.006 | 0.002 | 0.002 | 0.002 | 0.001 |
| 699.5 | -0.002 | 0.006 | 0.002 | 0.002 | 0.002 | 0.001 |
| 700 | -0.002 | 0.006 | 0.002 | 0.002 | 0.002 | 0.001 |
| 700.5 | -0.002 | 0.006 | 0.002 | 0.002 | 0.002 | 0.001 |
| 701 | -0.002 | 0.005 | 0.002 | 0.002 | 0.002 | 0.001 |
| 701.5 | -0.002 | 0.005 | 0.002 | 0.002 | 0.002 | 0.001 |
| 702 | -0.002 | 0.005 | 0.002 | 0.002 | 0.002 | 0.001 |
| 702.5 | -0.002 | 0.005 | 0.002 | 0.002 | 0.002 | 0.001 |
| 703 | -0.002 | 0.006 | 0.002 | 0.002 | 0.002 | 0.001 |
| 703.5 | -0.002 | 0.006 | 0.002 | 0.002 | 0.002 | 0.001 |
| 704 | -0.002 | 0.006 | 0.002 | 0.002 | 0.002 | 0.001 |
| 704.5 | -0.002 | 0.006 | 0.002 | 0.002 | 0.002 | 0.001 |
| 705 | -0.002 | 0.006 | 0.002 | 0.002 | 0.002 | 0.001 |
| 705.5 | -0.002 | 0.006 | 0.002 | 0.002 | 0.002 | 0.001 |
| 706 | -0.002 | 0.005 | 0.002 | 0.002 | 0.002 | 0.001 |
| 706.5 | -0.002 | 0.005 | 0.002 | 0.002 | 0.002 | 0.001 |
| 707 | -0.002 | 0.005 | 0.002 | 0.002 | 0.002 | 0.001 |
| 707.5 | -0.002 | 0.005 | 0.002 | 0.002 | 0.002 | 0.001 |
| 708 | -0.002 | 0.005 | 0.002 | 0.002 | 0.002 | 0.001 |
| 708.5 | -0.002 | 0.005 | 0.002 | 0.002 | 0.002 | 0.001 |
| 709 | -0.002 | 0.005 | 0.002 | 0.002 | 0.002 | 0.001 |
| 709.5 | -0.002 | 0.005 | 0.002 | 0.002 | 0.002 | 0.001 |
| 710 | -0.002 | 0.005 | 0.002 | 0.002 | 0.002 | 0.001 |
| 710.5 | -0.002 | 0.005 | 0.002 | 0.002 | 0.002 | 0.001 |
| 711 | -0.002 | 0.005 | 0.002 | 0.002 | 0.002 | 0.001 |
| 711.5 | -0.002 | 0.005 | 0.002 | 0.002 | 0.002 | 0.001 |
| 712 | -0.002 | 0.005 | 0.002 | 0.002 | 0.002 | 0.001 |
| 712.5 | -0.002 | 0.005 | 0.002 | 0.002 | 0.002 | 0.001 |
| 713 | -0.002 | 0.005 | 0.002 | 0.002 | 0.002 | 0.001 |
| 713.5 | -0.002 | 0.005 | 0.002 | 0.002 | 0.002 | 0.001 |
| 714 | -0.002 | 0.005 | 0.002 | 0.002 | 0.002 | 0.001 |
| 714.5 | -0.002 | 0.005 | 0.002 | 0.002 | 0.002 | 0.001 |
| 715 | -0.002 | 0.005 | 0.002 | 0.002 | 0.002 | 0.001 |
| 715.5 | -0.002 | 0.005 | 0.002 | 0.002 | 0.002 | 0.001 |
| 716 | -0.002 | 0.005 | 0.002 | 0.002 | 0.002 | 0.001 |
| 716.5 | -0.002 | 0.005 | 0.002 | 0.002 | 0.002 | 0.001 |
| 717 | -0.002 | 0.005 | 0.002 | 0.002 | 0.002 | 0.001 |
| 717.5 | -0.002 | 0.005 | 0.002 | 0.002 | 0.002 | 0.001 |
| 718 | -0.002 | 0.005 | 0.002 | 0.002 | 0.002 | 0.001 |
| 718.5 | -0.002 | 0.005 | 0.002 | 0.002 | 0.002 | 0.001 |
| 719 | -0.002 | 0.005 | 0.002 | 0.002 | 0.002 | 0.001 |
| 719.5 | -0.002 | 0.005 | 0.002 | 0.002 | 0.002 | 0.001 |
| 720 | -0.002 | 0.005 | 0.002 | 0.002 | 0.002 | 0.001 |
| 720.5 | -0.002 | 0.005 | 0.002 | 0.002 | 0.002 | 0.001 |
| 721 | -0.002 | 0.005 | 0.002 | 0.002 | 0.002 | 0.001 |
| 721.5 | -0.002 | 0.005 | 0.002 | 0.002 | 0.002 | 0.001 |
| 722 | -0.002 | 0.005 | 0.002 | 0.002 | 0.002 | 0.001 |
| 722.5 | -0.002 | 0.005 | 0.002 | 0.002 | 0.002 | 0.001 |
| 723 | -0.002 | 0.005 | 0.002 | 0.002 | 0.002 | 0.001 |
| 723.5 | -0.002 | 0.005 | 0.002 | 0.002 | 0.002 | 0.001 |
| 724 | -0.002 | 0.005 | 0.002 | 0.002 | 0.002 | 0.001 |
| 724.5 | -0.002 | 0.005 | 0.002 | 0.002 | 0.002 | 0.001 |
| 725 | -0.002 | 0.005 | 0.002 | 0.002 | 0.002 | 0.001 |
| 725.5 | -0.002 | 0.005 | 0.002 | 0.002 | 0.002 | 0.001 |
| 726 | -0.002 | 0.005 | 0.002 | 0.002 | 0.002 | 0.001 |
| 726.5 | -0.002 | 0.005 | 0.002 | 0.002 | 0.002 | 0.001 |
| 727 | -0.002 | 0.005 | 0.002 | 0.002 | 0.002 | 0.001 |
| 727.5 | -0.002 | 0.005 | 0.002 | 0.002 | 0.002 | 0.001 |
| 728 | -0.002 | 0.005 | 0.002 | 0.002 | 0.002 | 0.001 |
| 728.5 | -0.002 | 0.005 | 0.002 | 0.002 | 0.002 | 0.001 |
| 729 | -0.002 | 0.005 | 0.002 | 0.002 | 0.002 | 0.001 |
| 729.5 | -0.002 | 0.005 | 0.002 | 0.002 | 0.002 | 0.001 |
| 730 | -0.002 | 0.005 | 0.002 | 0.002 | 0.002 | 0.001 |
| 730.5 | -0.002 | 0.005 | 0.002 | 0.002 | 0.002 | 0.001 |
| 731 | -0.002 | 0.005 | 0.002 | 0.002 | 0.002 | 0.001 |
| 731.5 | -0.002 | 0.005 | 0.002 | 0.002 | 0.002 | 0.001 |
| 732 | -0.002 | 0.006 | 0.002 | 0.002 | 0.002 | 0.001 |
| 732.5 | -0.002 | 0.006 | 0.002 | 0.002 | 0.002 | 0.001 |
| 733 | -0.002 | 0.005 | 0.002 | 0.002 | 0.002 | 0.001 |
| 733.5 | -0.002 | 0.005 | 0.002 | 0.002 | 0.002 | 0.001 |
| 734 | -0.002 | 0.005 | 0.002 | 0.002 | 0.002 | 0.001 |
| 734.5 | -0.002 | 0.005 | 0.002 | 0.002 | 0.002 | 0.001 |
| 735 | -0.002 | 0.005 | 0.002 | 0.002 | 0.002 | 0.001 |
| 735.5 | -0.002 | 0.005 | 0.002 | 0.002 | 0.002 | 0.001 |
| 736 | -0.002 | 0.005 | 0.002 | 0.002 | 0.002 | 0.001 |
| 736.5 | -0.002 | 0.005 | 0.002 | 0.002 | 0.002 | 0.001 |
| 737 | -0.002 | 0.005 | 0.002 | 0.002 | 0.002 | 0.001 |
| 737.5 | -0.002 | 0.005 | 0.002 | 0.002 | 0.002 | 0.001 |
| 738 | -0.002 | 0.005 | 0.002 | 0.002 | 0.002 | 0.001 |
| 738.5 | -0.002 | 0.005 | 0.002 | 0.002 | 0.002 | 0.001 |
| 739 | -0.002 | 0.005 | 0.002 | 0.002 | 0.002 | 0.001 |
| 739.5 | -0.002 | 0.005 | 0.002 | 0.002 | 0.002 | 0.001 |
| 740 | -0.002 | 0.005 | 0.002 | 0.002 | 0.002 | 0.001 |
| 740.5 | -0.002 | 0.005 | 0.002 | 0.002 | 0.002 | 0.001 |
| 741 | -0.002 | 0.005 | 0.002 | 0.002 | 0.002 | 0.001 |
| 741.5 | -0.002 | 0.005 | 0.002 | 0.002 | 0.002 | 0.001 |
| 742 | -0.002 | 0.005 | 0.002 | 0.002 | 0.002 | 0.001 |
| 742.5 | -0.002 | 0.005 | 0.002 | 0.002 | 0.002 | 0 |
| 743 | -0.002 | 0.005 | 0.002 | 0.002 | 0.002 | 0 |
| 743.5 | -0.002 | 0.005 | 0.002 | 0.002 | 0.002 | 0.001 |
| 744 | -0.002 | 0.005 | 0.002 | 0.002 | 0.002 | 0.001 |
| 744.5 | -0.002 | 0.005 | 0.002 | 0.002 | 0.002 | 0.001 |
| 745 | -0.002 | 0.005 | 0.002 | 0.002 | 0.002 | 0 |
| 745.5 | -0.002 | 0.005 | 0.002 | 0.002 | 0.002 | 0 |
| 746 | -0.002 | 0.005 | 0.002 | 0.002 | 0.002 | 0 |
| 746.5 | -0.002 | 0.005 | 0.002 | 0.002 | 0.002 | 0 |
| 747 | -0.002 | 0.005 | 0.002 | 0.002 | 0.002 | 0 |
| 747.5 | -0.002 | 0.005 | 0.002 | 0.002 | 0.002 | 0 |
| 748 | -0.002 | 0.005 | 0.002 | 0.002 | 0.002 | 0 |
| 748.5 | -0.002 | 0.005 | 0.002 | 0.002 | 0.002 | 0 |
| 749 | -0.002 | 0.005 | 0.002 | 0.002 | 0.002 | 0 |
| 749.5 | -0.002 | 0.005 | 0.002 | 0.002 | 0.002 | 0 |
| 750 | -0.002 | 0.005 | 0.002 | 0.002 | 0.002 | 0 |
| 750.5 | -0.002 | 0.005 | 0.002 | 0.002 | 0.002 | 0 |
| 751 | -0.003 | 0.005 | 0.002 | 0.002 | 0.002 | 0 |
| 751.5 | -0.003 | 0.005 | 0.002 | 0.002 | 0.002 | 0 |
| 752 | -0.003 | 0.005 | 0.002 | 0.002 | 0.002 | 0 |
| 752.5 | -0.003 | 0.005 | 0.002 | 0.002 | 0.002 | 0 |
| 753 | -0.002 | 0.005 | 0.002 | 0.002 | 0.002 | 0 |
| 753.5 | -0.002 | 0.005 | 0.002 | 0.002 | 0.002 | 0 |
| 754 | -0.002 | 0.005 | 0.002 | 0.002 | 0.002 | 0 |
| 754.5 | -0.002 | 0.005 | 0.002 | 0.002 | 0.002 | 0 |
| 755 | -0.002 | 0.005 | 0.002 | 0.002 | 0.002 | 0 |
| 755.5 | -0.002 | 0.005 | 0.002 | 0.002 | 0.002 | 0 |
| 756 | -0.002 | 0.005 | 0.002 | 0.002 | 0.002 | 0 |
| 756.5 | -0.002 | 0.005 | 0.002 | 0.002 | 0.002 | 0 |
| 757 | -0.002 | 0.005 | 0.002 | 0.002 | 0.002 | 0 |
| 757.5 | -0.002 | 0.005 | 0.002 | 0.002 | 0.002 | 0 |
| 758 | -0.002 | 0.005 | 0.002 | 0.002 | 0.002 | 0 |
| 758.5 | -0.002 | 0.005 | 0.002 | 0.002 | 0.002 | 0 |
| 759 | -0.002 | 0.005 | 0.002 | 0.002 | 0.002 | 0 |
| 759.5 | -0.002 | 0.005 | 0.002 | 0.002 | 0.002 | 0 |
| 760 | -0.002 | 0.005 | 0.002 | 0.002 | 0.002 | 0 |
| 760.5 | -0.002 | 0.005 | 0.002 | 0.002 | 0.002 | 0 |
| 761 | -0.002 | 0.005 | 0.002 | 0.002 | 0.002 | 0 |
| 761.5 | -0.002 | 0.005 | 0.002 | 0.002 | 0.002 | 0 |
| 762 | -0.002 | 0.005 | 0.002 | 0.002 | 0.002 | 0 |
| 762.5 | -0.002 | 0.005 | 0.002 | 0.002 | 0.002 | 0 |
| 763 | -0.002 | 0.005 | 0.002 | 0.002 | 0.002 | 0 |
| 763.5 | -0.002 | 0.005 | 0.002 | 0.002 | 0.002 | 0 |
| 764 | -0.002 | 0.005 | 0.002 | 0.002 | 0.002 | 0 |
| 764.5 | -0.002 | 0.005 | 0.002 | 0.002 | 0.002 | 0 |
| 765 | -0.002 | 0.005 | 0.002 | 0.002 | 0.002 | 0 |
| 765.5 | -0.002 | 0.005 | 0.002 | 0.002 | 0.002 | 0 |
| 766 | -0.002 | 0.005 | 0.002 | 0.002 | 0.002 | 0 |
| 766.5 | -0.002 | 0.005 | 0.002 | 0.002 | 0.002 | 0 |
| 767 | -0.002 | 0.005 | 0.002 | 0.002 | 0.002 | 0.001 |
| 767.5 | -0.002 | 0.005 | 0.002 | 0.002 | 0.002 | 0.001 |
| 768 | -0.002 | 0.005 | 0.002 | 0.002 | 0.002 | 0.001 |
| 768.5 | -0.002 | 0.005 | 0.002 | 0.002 | 0.002 | 0.001 |
| 769 | -0.002 | 0.005 | 0.002 | 0.002 | 0.002 | 0.001 |
| 769.5 | -0.002 | 0.005 | 0.002 | 0.002 | 0.002 | 0.001 |
| 770 | -0.002 | 0.005 | 0.002 | 0.002 | 0.002 | 0.001 |
| 770.5 | -0.002 | 0.005 | 0.002 | 0.002 | 0.002 | 0.001 |
| 771 | -0.002 | 0.005 | 0.003 | 0.002 | 0.002 | 0.001 |
| 771.5 | -0.002 | 0.005 | 0.003 | 0.002 | 0.002 | 0.001 |
| 772 | -0.002 | 0.005 | 0.003 | 0.002 | 0.002 | 0.001 |
| 772.5 | -0.002 | 0.005 | 0.002 | 0.002 | 0.002 | 0.001 |
| 773 | -0.002 | 0.005 | 0.002 | 0.002 | 0.002 | 0 |
| 773.5 | -0.002 | 0.005 | 0.002 | 0.002 | 0.002 | 0 |
| 774 | -0.002 | 0.005 | 0.003 | 0.002 | 0.002 | 0.001 |
| 774.5 | -0.002 | 0.005 | 0.003 | 0.002 | 0.002 | 0.001 |
| 775 | -0.002 | 0.005 | 0.003 | 0.002 | 0.002 | 0 |
| 775.5 | -0.002 | 0.005 | 0.003 | 0.002 | 0.002 | 0 |
| 776 | -0.002 | 0.005 | 0.002 | 0.002 | 0.002 | 0 |
| 776.5 | -0.002 | 0.005 | 0.002 | 0.002 | 0.002 | 0 |
| 777 | -0.002 | 0.005 | 0.002 | 0.002 | 0.002 | 0 |
| 777.5 | -0.002 | 0.005 | 0.002 | 0.002 | 0.002 | 0 |
| 778 | -0.002 | 0.005 | 0.002 | 0.002 | 0.002 | 0 |
| 778.5 | -0.002 | 0.005 | 0.002 | 0.002 | 0.002 | 0 |
| 779 | -0.002 | 0.005 | 0.002 | 0.002 | 0.002 | 0 |
| 779.5 | -0.002 | 0.005 | 0.002 | 0.002 | 0.002 | 0 |
| 780 | -0.002 | 0.005 | 0.002 | 0.002 | 0.002 | 0 |
| 780.5 | -0.002 | 0.005 | 0.002 | 0.002 | 0.002 | 0 |
| 781 | -0.002 | 0.005 | 0.002 | 0.002 | 0.002 | 0 |
| 781.5 | -0.002 | 0.005 | 0.002 | 0.002 | 0.002 | 0 |
| 782 | -0.002 | 0.005 | 0.002 | 0.002 | 0.002 | 0 |
| 782.5 | -0.002 | 0.005 | 0.002 | 0.002 | 0.002 | 0 |
| 783 | -0.002 | 0.005 | 0.002 | 0.002 | 0.002 | 0 |
| 783.5 | -0.002 | 0.005 | 0.002 | 0.002 | 0.002 | 0 |
| 784 | -0.002 | 0.005 | 0.002 | 0.002 | 0.002 | 0 |
| 784.5 | -0.002 | 0.005 | 0.002 | 0.002 | 0.002 | 0 |
| 785 | -0.002 | 0.005 | 0.002 | 0.001 | 0.002 | 0 |
| 785.5 | -0.002 | 0.005 | 0.002 | 0.001 | 0.002 | 0 |
| 786 | -0.002 | 0.005 | 0.002 | 0.001 | 0.002 | 0 |
| 786.5 | -0.002 | 0.005 | 0.002 | 0.001 | 0.002 | 0 |
| 787 | -0.002 | 0.005 | 0.002 | 0.001 | 0.002 | 0 |
| 787.5 | -0.003 | 0.005 | 0.002 | 0.001 | 0.002 | 0 |
| 788 | -0.003 | 0.005 | 0.002 | 0.001 | 0.002 | 0 |
| 788.5 | -0.003 | 0.005 | 0.002 | 0.001 | 0.002 | 0 |
| 789 | -0.003 | 0.005 | 0.002 | 0.001 | 0.002 | 0 |
| 789.5 | -0.003 | 0.005 | 0.002 | 0.001 | 0.002 | 0 |
| 790 | -0.003 | 0.005 | 0.002 | 0.001 | 0.002 | 0 |
| 790.5 | -0.003 | 0.005 | 0.002 | 0.001 | 0.002 | 0 |
| 791 | -0.003 | 0.005 | 0.002 | 0.001 | 0.002 | 0 |
| 791.5 | -0.003 | 0.005 | 0.002 | 0.001 | 0.002 | 0 |
| 792 | -0.003 | 0.005 | 0.002 | 0.001 | 0.002 | 0 |
| 792.5 | -0.003 | 0.005 | 0.002 | 0.001 | 0.002 | 0 |
| 793 | -0.003 | 0.005 | 0.002 | 0.001 | 0.002 | 0 |
| 793.5 | -0.003 | 0.005 | 0.002 | 0.001 | 0.002 | 0 |
| 794 | -0.003 | 0.005 | 0.002 | 0.001 | 0.002 | 0 |
| 794.5 | -0.003 | 0.005 | 0.002 | 0.001 | 0.002 | 0 |
| 795 | -0.003 | 0.005 | 0.002 | 0.001 | 0.002 | 0 |
| 795.5 | -0.003 | 0.005 | 0.002 | 0.001 | 0.002 | 0 |
| 796 | -0.003 | 0.005 | 0.002 | 0.001 | 0.002 | 0 |
| 796.5 | -0.003 | 0.005 | 0.002 | 0.001 | 0.002 | 0 |
| 797 | -0.003 | 0.005 | 0.002 | 0.001 | 0.002 | 0 |
| 797.5 | -0.003 | 0.005 | 0.002 | 0.001 | 0.002 | 0 |
| 798 | -0.003 | 0.005 | 0.002 | 0.001 | 0.002 | 0 |
| 798.5 | -0.003 | 0.005 | 0.002 | 0.001 | 0.002 | 0 |
| 799 | -0.003 | 0.005 | 0.002 | 0.001 | 0.002 | 0 |
| 799.5 | -0.003 | 0.005 | 0.002 | 0.001 | 0.002 | 0 |
| 800 | -0.003 | 0.005 | 0.002 | 0.001 | 0.002 | 0 |

**Table S3.** UV-vis absorption data of single amino acid derivative of tyrosine peptide (BTTP) on UV-A light irradiation with respect to time.

| **Wavelength (nm)** | **Absorbance (a.u)** | | | | | |
| --- | --- | --- | --- | --- | --- | --- |
|  | **5 min** | **10 min** | **15 min** | **20 min** | **25 min** | **30 min** |
| 600 | -0.024 | -0.276 | -0.276 | -0.276 | -0.276 | -0.276 |
| 599.5 | -0.024 | -0.278 | -0.278 | -0.278 | -0.278 | -0.278 |
| 599 | -0.024 | -0.282 | -0.282 | -0.282 | -0.282 | -0.282 |
| 598.5 | -0.024 | -0.285 | -0.285 | -0.285 | -0.285 | -0.285 |
| 598 | -0.024 | -0.289 | -0.289 | -0.289 | -0.289 | -0.289 |
| 597.5 | -0.024 | -0.294 | -0.294 | -0.294 | -0.294 | -0.294 |
| 597 | -0.024 | -0.299 | -0.299 | -0.299 | -0.299 | -0.299 |
| 596.5 | -0.024 | -0.304 | -0.304 | -0.304 | -0.304 | -0.304 |
| 596 | -0.024 | -0.308 | -0.308 | -0.308 | -0.308 | -0.308 |
| 595.5 | -0.023 | -0.312 | -0.312 | -0.312 | -0.312 | -0.312 |
| 595 | -0.023 | -0.316 | -0.316 | -0.316 | -0.316 | -0.316 |
| 594.5 | -0.023 | -0.32 | -0.32 | -0.32 | -0.32 | -0.32 |
| 594 | -0.023 | -0.323 | -0.323 | -0.323 | -0.323 | -0.323 |
| 593.5 | -0.023 | -0.326 | -0.326 | -0.326 | -0.326 | -0.326 |
| 593 | -0.023 | -0.33 | -0.33 | -0.33 | -0.33 | -0.33 |
| 592.5 | -0.023 | -0.335 | -0.335 | -0.335 | -0.335 | -0.335 |
| 592 | -0.023 | -0.339 | -0.339 | -0.339 | -0.339 | -0.339 |
| 591.5 | -0.023 | -0.342 | -0.342 | -0.342 | -0.342 | -0.342 |
| 591 | -0.023 | -0.345 | -0.345 | -0.345 | -0.345 | -0.345 |
| 590.5 | -0.023 | -0.349 | -0.349 | -0.349 | -0.349 | -0.349 |
| 590 | -0.023 | -0.351 | -0.351 | -0.351 | -0.351 | -0.351 |
| 589.5 | -0.023 | -0.354 | -0.354 | -0.354 | -0.354 | -0.354 |
| 589 | -0.023 | -0.357 | -0.357 | -0.357 | -0.357 | -0.357 |
| 588.5 | -0.023 | -0.36 | -0.36 | -0.36 | -0.36 | -0.36 |
| 588 | -0.023 | -0.363 | -0.363 | -0.363 | -0.363 | -0.363 |
| 587.5 | -0.023 | -0.366 | -0.366 | -0.366 | -0.366 | -0.366 |
| 587 | -0.023 | -0.369 | -0.369 | -0.369 | -0.369 | -0.369 |
| 586.5 | -0.022 | -0.371 | -0.371 | -0.371 | -0.371 | -0.371 |
| 586 | -0.022 | -0.374 | -0.374 | -0.374 | -0.374 | -0.374 |
| 585.5 | -0.022 | -0.376 | -0.376 | -0.376 | -0.376 | -0.376 |
| 585 | -0.022 | -0.378 | -0.378 | -0.378 | -0.378 | -0.378 |
| 584.5 | -0.022 | -0.38 | -0.38 | -0.38 | -0.38 | -0.38 |
| 584 | -0.022 | -0.382 | -0.382 | -0.382 | -0.382 | -0.382 |
| 583.5 | -0.022 | -0.384 | -0.384 | -0.384 | -0.384 | -0.384 |
| 583 | -0.022 | -0.386 | -0.386 | -0.386 | -0.386 | -0.386 |
| 582.5 | -0.022 | -0.389 | -0.389 | -0.389 | -0.389 | -0.389 |
| 582 | -0.022 | -0.391 | -0.391 | -0.391 | -0.391 | -0.391 |
| 581.5 | -0.022 | -0.393 | -0.393 | -0.393 | -0.393 | -0.393 |
| 581 | -0.022 | -0.395 | -0.395 | -0.395 | -0.395 | -0.395 |
| 580.5 | -0.021 | -0.396 | -0.396 | -0.396 | -0.396 | -0.396 |
| 580 | -0.021 | -0.398 | -0.398 | -0.398 | -0.398 | -0.398 |
| 579.5 | -0.021 | -0.399 | -0.399 | -0.399 | -0.399 | -0.399 |
| 579 | -0.021 | -0.401 | -0.401 | -0.401 | -0.401 | -0.401 |
| 578.5 | -0.021 | -0.402 | -0.402 | -0.402 | -0.402 | -0.402 |
| 578 | -0.021 | -0.404 | -0.404 | -0.404 | -0.404 | -0.404 |
| 577.5 | -0.021 | -0.405 | -0.405 | -0.405 | -0.405 | -0.405 |
| 577 | -0.021 | -0.406 | -0.406 | -0.406 | -0.406 | -0.406 |
| 576.5 | -0.021 | -0.408 | -0.408 | -0.408 | -0.408 | -0.408 |
| 576 | -0.02 | -0.409 | -0.409 | -0.409 | -0.409 | -0.409 |
| 575.5 | -0.02 | -0.411 | -0.411 | -0.411 | -0.411 | -0.411 |
| 575 | -0.02 | -0.412 | -0.412 | -0.412 | -0.412 | -0.412 |
| 574.5 | -0.02 | -0.414 | -0.414 | -0.414 | -0.414 | -0.414 |
| 574 | -0.02 | -0.415 | -0.415 | -0.415 | -0.415 | -0.415 |
| 573.5 | -0.02 | -0.416 | -0.416 | -0.416 | -0.416 | -0.416 |
| 573 | -0.02 | -0.417 | -0.417 | -0.417 | -0.417 | -0.417 |
| 572.5 | -0.02 | -0.418 | -0.418 | -0.418 | -0.418 | -0.418 |
| 572 | -0.019 | -0.42 | -0.42 | -0.42 | -0.42 | -0.42 |
| 571.5 | -0.019 | -0.421 | -0.421 | -0.421 | -0.421 | -0.421 |
| 571 | -0.019 | -0.422 | -0.422 | -0.422 | -0.422 | -0.422 |
| 570.5 | -0.019 | -0.424 | -0.424 | -0.424 | -0.424 | -0.424 |
| 570 | -0.019 | -0.425 | -0.425 | -0.425 | -0.425 | -0.425 |
| 569.5 | -0.019 | -0.426 | -0.426 | -0.426 | -0.426 | -0.426 |
| 569 | -0.018 | -0.427 | -0.427 | -0.427 | -0.427 | -0.427 |
| 568.5 | -0.018 | -0.428 | -0.428 | -0.428 | -0.428 | -0.428 |
| 568 | -0.018 | -0.429 | -0.429 | -0.429 | -0.429 | -0.429 |
| 567.5 | -0.018 | -0.43 | -0.43 | -0.43 | -0.43 | -0.43 |
| 567 | -0.018 | -0.432 | -0.432 | -0.432 | -0.432 | -0.432 |
| 566.5 | -0.018 | -0.433 | -0.433 | -0.433 | -0.433 | -0.433 |
| 566 | -0.018 | -0.434 | -0.434 | -0.434 | -0.434 | -0.434 |
| 565.5 | -0.017 | -0.435 | -0.435 | -0.435 | -0.435 | -0.435 |
| 565 | -0.017 | -0.436 | -0.436 | -0.436 | -0.436 | -0.436 |
| 564.5 | -0.017 | -0.437 | -0.437 | -0.437 | -0.437 | -0.437 |
| 564 | -0.017 | -0.439 | -0.439 | -0.439 | -0.439 | -0.439 |
| 563.5 | -0.017 | -0.44 | -0.44 | -0.44 | -0.44 | -0.44 |
| 563 | -0.017 | -0.441 | -0.441 | -0.441 | -0.441 | -0.441 |
| 562.5 | -0.017 | -0.442 | -0.442 | -0.442 | -0.442 | -0.442 |
| 562 | -0.016 | -0.443 | -0.443 | -0.443 | -0.443 | -0.443 |
| 561.5 | -0.016 | -0.445 | -0.445 | -0.445 | -0.445 | -0.445 |
| 561 | -0.016 | -0.442 | -0.446 | -0.446 | -0.446 | -0.446 |
| 560.5 | -0.016 | -0.427 | -0.439 | -0.44 | -0.447 | -0.438 |
| 560 | -0.016 | -0.408 | -0.422 | -0.423 | -0.438 | -0.42 |
| 559.5 | -0.015 | -0.389 | -0.402 | -0.403 | -0.418 | -0.4 |
| 559 | -0.015 | -0.369 | -0.383 | -0.384 | -0.398 | -0.381 |
| 558.5 | -0.015 | -0.35 | -0.363 | -0.364 | -0.377 | -0.362 |
| 558 | -0.015 | -0.332 | -0.344 | -0.344 | -0.358 | -0.343 |
| 557.5 | -0.015 | -0.315 | -0.326 | -0.325 | -0.339 | -0.326 |
| 557 | -0.015 | -0.3 | -0.31 | -0.31 | -0.322 | -0.31 |
| 556.5 | -0.015 | -0.285 | -0.295 | -0.294 | -0.306 | -0.295 |
| 556 | -0.014 | -0.271 | -0.28 | -0.279 | -0.291 | -0.28 |
| 555.5 | -0.014 | -0.257 | -0.266 | -0.265 | -0.277 | -0.267 |
| 555 | -0.014 | -0.244 | -0.253 | -0.251 | -0.262 | -0.254 |
| 554.5 | -0.014 | -0.232 | -0.24 | -0.238 | -0.249 | -0.241 |
| 554 | -0.014 | -0.221 | -0.229 | -0.226 | -0.238 | -0.23 |
| 553.5 | -0.013 | -0.21 | -0.217 | -0.215 | -0.226 | -0.22 |
| 553 | -0.013 | -0.2 | -0.206 | -0.204 | -0.214 | -0.209 |
| 552.5 | -0.013 | -0.191 | -0.197 | -0.194 | -0.204 | -0.2 |
| 552 | -0.013 | -0.182 | -0.188 | -0.185 | -0.195 | -0.192 |
| 551.5 | -0.013 | -0.175 | -0.18 | -0.176 | -0.187 | -0.183 |
| 551 | -0.013 | -0.167 | -0.173 | -0.169 | -0.178 | -0.176 |
| 550.5 | -0.013 | -0.16 | -0.166 | -0.161 | -0.17 | -0.17 |
| 550 | -0.012 | -0.153 | -0.159 | -0.154 | -0.163 | -0.163 |
| 549.5 | -0.012 | -0.147 | -0.152 | -0.149 | -0.157 | -0.157 |
| 549 | -0.012 | -0.142 | -0.147 | -0.143 | -0.151 | -0.152 |
| 548.5 | -0.012 | -0.136 | -0.141 | -0.137 | -0.145 | -0.147 |
| 548 | -0.012 | -0.131 | -0.136 | -0.132 | -0.14 | -0.142 |
| 547.5 | -0.012 | -0.127 | -0.132 | -0.127 | -0.136 | -0.138 |
| 547 | -0.012 | -0.123 | -0.127 | -0.123 | -0.131 | -0.134 |
| 546.5 | -0.011 | -0.119 | -0.123 | -0.119 | -0.127 | -0.13 |
| 546 | -0.011 | -0.115 | -0.119 | -0.115 | -0.123 | -0.126 |
| 545.5 | -0.011 | -0.112 | -0.116 | -0.111 | -0.119 | -0.123 |
| 545 | -0.011 | -0.108 | -0.112 | -0.108 | -0.115 | -0.12 |
| 544.5 | -0.011 | -0.104 | -0.109 | -0.104 | -0.112 | -0.117 |
| 544 | -0.011 | -0.102 | -0.105 | -0.101 | -0.109 | -0.114 |
| 543.5 | -0.011 | -0.098 | -0.102 | -0.097 | -0.105 | -0.11 |
| 543 | -0.011 | -0.095 | -0.098 | -0.094 | -0.101 | -0.107 |
| 542.5 | -0.01 | -0.091 | -0.095 | -0.09 | -0.097 | -0.104 |
| 542 | -0.01 | -0.088 | -0.091 | -0.086 | -0.094 | -0.101 |
| 541.5 | -0.01 | -0.084 | -0.088 | -0.083 | -0.091 | -0.097 |
| 541 | -0.01 | -0.081 | -0.084 | -0.08 | -0.087 | -0.094 |
| 540.5 | -0.01 | -0.078 | -0.081 | -0.076 | -0.083 | -0.091 |
| 540 | -0.009 | -0.075 | -0.078 | -0.073 | -0.08 | -0.088 |
| 539.5 | -0.01 | -0.071 | -0.075 | -0.07 | -0.077 | -0.084 |
| 539 | -0.01 | -0.068 | -0.071 | -0.066 | -0.073 | -0.081 |
| 538.5 | -0.01 | -0.065 | -0.068 | -0.063 | -0.07 | -0.078 |
| 538 | -0.01 | -0.062 | -0.065 | -0.06 | -0.067 | -0.075 |
| 537.5 | -0.01 | -0.058 | -0.061 | -0.056 | -0.064 | -0.072 |
| 537 | -0.01 | -0.055 | -0.058 | -0.053 | -0.06 | -0.069 |
| 536.5 | -0.01 | -0.052 | -0.055 | -0.05 | -0.056 | -0.066 |
| 536 | -0.009 | -0.049 | -0.052 | -0.047 | -0.053 | -0.063 |
| 535.5 | -0.009 | -0.047 | -0.049 | -0.044 | -0.051 | -0.061 |
| 535 | -0.009 | -0.044 | -0.046 | -0.041 | -0.048 | -0.058 |
| 534.5 | -0.009 | -0.041 | -0.044 | -0.038 | -0.045 | -0.056 |
| 534 | -0.009 | -0.039 | -0.042 | -0.036 | -0.043 | -0.053 |
| 533.5 | -0.009 | -0.037 | -0.04 | -0.035 | -0.042 | -0.052 |
| 533 | -0.009 | -0.035 | -0.038 | -0.033 | -0.04 | -0.05 |
| 532.5 | -0.009 | -0.034 | -0.037 | -0.032 | -0.039 | -0.049 |
| 532 | -0.009 | -0.033 | -0.035 | -0.03 | -0.037 | -0.047 |
| 531.5 | -0.009 | -0.031 | -0.034 | -0.029 | -0.036 | -0.046 |
| 531 | -0.009 | -0.03 | -0.032 | -0.027 | -0.034 | -0.045 |
| 530.5 | -0.009 | -0.028 | -0.03 | -0.025 | -0.032 | -0.043 |
| 530 | -0.009 | -0.025 | -0.028 | -0.023 | -0.029 | -0.041 |
| 529.5 | -0.009 | -0.023 | -0.026 | -0.021 | -0.027 | -0.039 |
| 529 | -0.009 | -0.021 | -0.023 | -0.018 | -0.025 | -0.037 |
| 528.5 | -0.008 | -0.019 | -0.021 | -0.017 | -0.023 | -0.035 |
| 528 | -0.008 | -0.018 | -0.02 | -0.015 | -0.022 | -0.034 |
| 527.5 | -0.008 | -0.016 | -0.019 | -0.014 | -0.021 | -0.032 |
| 527 | -0.008 | -0.016 | -0.018 | -0.014 | -0.02 | -0.031 |
| 526.5 | -0.008 | -0.016 | -0.018 | -0.013 | -0.019 | -0.031 |
| 526 | -0.008 | -0.016 | -0.018 | -0.013 | -0.02 | -0.031 |
| 525.5 | -0.008 | -0.016 | -0.018 | -0.014 | -0.021 | -0.032 |
| 525 | -0.008 | -0.016 | -0.019 | -0.014 | -0.021 | -0.033 |
| 524.5 | -0.008 | -0.017 | -0.02 | -0.015 | -0.022 | -0.033 |
| 524 | -0.008 | -0.018 | -0.02 | -0.016 | -0.022 | -0.034 |
| 523.5 | -0.008 | -0.018 | -0.02 | -0.016 | -0.023 | -0.034 |
| 523 | -0.008 | -0.018 | -0.02 | -0.016 | -0.023 | -0.034 |
| 522.5 | -0.008 | -0.018 | -0.02 | -0.016 | -0.023 | -0.034 |
| 522 | -0.008 | -0.018 | -0.02 | -0.016 | -0.023 | -0.034 |
| 521.5 | -0.008 | -0.018 | -0.02 | -0.016 | -0.023 | -0.033 |
| 521 | -0.007 | -0.017 | -0.02 | -0.016 | -0.022 | -0.033 |
| 520.5 | -0.007 | -0.016 | -0.018 | -0.015 | -0.021 | -0.032 |
| 520 | -0.007 | -0.015 | -0.017 | -0.013 | -0.02 | -0.03 |
| 519.5 | -0.007 | -0.014 | -0.015 | -0.012 | -0.018 | -0.029 |
| 519 | -0.007 | -0.012 | -0.014 | -0.01 | -0.017 | -0.027 |
| 518.5 | -0.007 | -0.01 | -0.013 | -0.009 | -0.015 | -0.025 |
| 518 | -0.007 | -0.008 | -0.011 | -0.007 | -0.014 | -0.024 |
| 517.5 | -0.007 | -0.007 | -0.009 | -0.006 | -0.012 | -0.022 |
| 517 | -0.007 | -0.006 | -0.008 | -0.005 | -0.011 | -0.021 |
| 516.5 | -0.007 | -0.005 | -0.008 | -0.004 | -0.01 | -0.02 |
| 516 | -0.007 | -0.005 | -0.007 | -0.004 | -0.01 | -0.019 |
| 515.5 | -0.007 | -0.005 | -0.007 | -0.004 | -0.01 | -0.019 |
| 515 | -0.007 | -0.005 | -0.008 | -0.004 | -0.011 | -0.019 |
| 514.5 | -0.007 | -0.006 | -0.009 | -0.006 | -0.012 | -0.02 |
| 514 | -0.006 | -0.007 | -0.01 | -0.006 | -0.013 | -0.021 |
| 513.5 | -0.006 | -0.008 | -0.011 | -0.008 | -0.014 | -0.022 |
| 513 | -0.006 | -0.009 | -0.012 | -0.009 | -0.016 | -0.023 |
| 512.5 | -0.006 | -0.01 | -0.013 | -0.01 | -0.016 | -0.024 |
| 512 | -0.006 | -0.011 | -0.013 | -0.01 | -0.017 | -0.024 |
| 511.5 | -0.006 | -0.011 | -0.014 | -0.011 | -0.018 | -0.024 |
| 511 | -0.006 | -0.012 | -0.015 | -0.012 | -0.018 | -0.024 |
| 510.5 | -0.006 | -0.012 | -0.014 | -0.012 | -0.018 | -0.024 |
| 510 | -0.006 | -0.012 | -0.014 | -0.011 | -0.018 | -0.024 |
| 509.5 | -0.006 | -0.012 | -0.014 | -0.011 | -0.018 | -0.024 |
| 509 | -0.006 | -0.011 | -0.014 | -0.011 | -0.018 | -0.023 |
| 508.5 | -0.006 | -0.011 | -0.014 | -0.011 | -0.018 | -0.023 |
| 508 | -0.005 | -0.011 | -0.013 | -0.011 | -0.017 | -0.023 |
| 507.5 | -0.005 | -0.01 | -0.013 | -0.01 | -0.017 | -0.022 |
| 507 | -0.005 | -0.01 | -0.012 | -0.01 | -0.016 | -0.021 |
| 506.5 | -0.005 | -0.01 | -0.012 | -0.01 | -0.016 | -0.021 |
| 506 | -0.005 | -0.009 | -0.012 | -0.009 | -0.016 | -0.02 |
| 505.5 | -0.005 | -0.009 | -0.012 | -0.009 | -0.015 | -0.019 |
| 505 | -0.005 | -0.009 | -0.011 | -0.009 | -0.015 | -0.019 |
| 504.5 | -0.005 | -0.008 | -0.011 | -0.008 | -0.015 | -0.018 |
| 504 | -0.005 | -0.008 | -0.01 | -0.008 | -0.014 | -0.018 |
| 503.5 | -0.005 | -0.007 | -0.01 | -0.007 | -0.014 | -0.017 |
| 503 | -0.005 | -0.007 | -0.01 | -0.007 | -0.014 | -0.017 |
| 502.5 | -0.005 | -0.007 | -0.009 | -0.007 | -0.013 | -0.016 |
| 502 | -0.005 | -0.006 | -0.009 | -0.007 | -0.013 | -0.016 |
| 501.5 | -0.005 | -0.006 | -0.009 | -0.006 | -0.013 | -0.016 |
| 501 | -0.005 | -0.006 | -0.008 | -0.006 | -0.012 | -0.015 |
| 500.5 | -0.004 | -0.005 | -0.008 | -0.006 | -0.012 | -0.015 |
| 500 | -0.004 | -0.005 | -0.008 | -0.006 | -0.012 | -0.014 |
| 499.5 | -0.004 | -0.005 | -0.008 | -0.005 | -0.011 | -0.014 |
| 499 | -0.004 | -0.005 | -0.007 | -0.005 | -0.011 | -0.014 |
| 498.5 | -0.004 | -0.005 | -0.007 | -0.005 | -0.011 | -0.013 |
| 498 | -0.004 | -0.005 | -0.007 | -0.005 | -0.011 | -0.013 |
| 497.5 | -0.004 | -0.004 | -0.007 | -0.005 | -0.011 | -0.013 |
| 497 | -0.004 | -0.004 | -0.007 | -0.004 | -0.01 | -0.012 |
| 496.5 | -0.004 | -0.003 | -0.006 | -0.004 | -0.01 | -0.012 |
| 496 | -0.004 | -0.003 | -0.006 | -0.004 | -0.01 | -0.011 |
| 495.5 | -0.004 | -0.003 | -0.005 | -0.003 | -0.01 | -0.011 |
| 495 | -0.003 | -0.003 | -0.005 | -0.003 | -0.009 | -0.011 |
| 494.5 | -0.003 | -0.002 | -0.005 | -0.003 | -0.009 | -0.01 |
| 494 | -0.003 | -0.002 | -0.005 | -0.003 | -0.009 | -0.01 |
| 493.5 | -0.003 | -0.002 | -0.005 | -0.003 | -0.009 | -0.01 |
| 493 | -0.003 | -0.002 | -0.005 | -0.003 | -0.009 | -0.01 |
| 492.5 | -0.003 | -0.002 | -0.005 | -0.002 | -0.008 | -0.01 |
| 492 | -0.003 | -0.002 | -0.004 | -0.002 | -0.008 | -0.01 |
| 491.5 | -0.003 | -0.001 | -0.004 | -0.002 | -0.008 | -0.009 |
| 491 | -0.003 | -0.001 | -0.004 | -0.002 | -0.008 | -0.009 |
| 490.5 | -0.003 | -0.001 | -0.004 | -0.002 | -0.008 | -0.009 |
| 490 | -0.003 | -0.001 | -0.003 | -0.001 | -0.007 | -0.008 |
| 489.5 | -0.003 | 0 | -0.003 | -0.001 | -0.007 | -0.008 |
| 489 | -0.002 | 0 | -0.003 | -0.001 | -0.007 | -0.008 |
| 488.5 | -0.002 | 0 | -0.003 | -0.001 | -0.006 | -0.008 |
| 488 | -0.002 | 0 | -0.002 | -0.001 | -0.006 | -0.007 |
| 487.5 | -0.002 | 0 | -0.002 | 0 | -0.006 | -0.007 |
| 487 | -0.002 | 0 | -0.002 | -0.001 | -0.006 | -0.007 |
| 486.5 | -0.002 | 0 | -0.002 | 0 | -0.006 | -0.007 |
| 486 | -0.002 | 0.001 | -0.002 | 0 | -0.006 | -0.007 |
| 485.5 | -0.002 | 0.001 | -0.002 | 0 | -0.006 | -0.007 |
| 485 | -0.002 | 0.001 | -0.002 | 0 | -0.005 | -0.007 |
| 484.5 | -0.002 | 0.001 | -0.001 | 0.001 | -0.005 | -0.006 |
| 484 | -0.002 | 0.002 | -0.001 | 0.001 | -0.005 | -0.006 |
| 483.5 | -0.002 | 0.002 | -0.001 | 0.001 | -0.004 | -0.006 |
| 483 | -0.002 | 0.002 | 0 | 0.001 | -0.004 | -0.005 |
| 482.5 | -0.002 | 0.002 | 0 | 0.002 | -0.004 | -0.005 |
| 482 | -0.002 | 0.003 | 0 | 0.002 | -0.004 | -0.005 |
| 481.5 | -0.002 | 0.003 | 0 | 0.002 | -0.003 | -0.004 |
| 481 | -0.002 | 0.003 | 0.001 | 0.002 | -0.003 | -0.004 |
| 480.5 | -0.002 | 0.004 | 0.001 | 0.003 | -0.003 | -0.004 |
| 480 | -0.001 | 0.004 | 0.001 | 0.003 | -0.002 | -0.003 |
| 479.5 | -0.001 | 0.004 | 0.001 | 0.003 | -0.002 | -0.003 |
| 479 | -0.001 | 0.005 | 0.002 | 0.004 | -0.002 | -0.002 |
| 478.5 | -0.001 | 0.005 | 0.003 | 0.005 | -0.001 | -0.002 |
| 478 | -0.001 | 0.005 | 0.003 | 0.005 | -0.001 | -0.002 |
| 477.5 | -0.001 | 0.005 | 0.003 | 0.005 | -0.001 | -0.001 |
| 477 | -0.001 | 0.006 | 0.003 | 0.005 | 0 | -0.001 |
| 476.5 | -0.001 | 0.006 | 0.004 | 0.005 | 0 | -0.001 |
| 476 | -0.001 | 0.006 | 0.004 | 0.006 | 0 | 0 |
| 475.5 | -0.001 | 0.007 | 0.005 | 0.006 | 0.001 | 0 |
| 475 | -0.001 | 0.007 | 0.005 | 0.006 | 0.001 | 0 |
| 474.5 | -0.001 | 0.008 | 0.005 | 0.007 | 0.002 | 0.001 |
| 474 | 0 | 0.008 | 0.006 | 0.007 | 0.002 | 0.001 |
| 473.5 | 0 | 0.008 | 0.006 | 0.008 | 0.003 | 0.001 |
| 473 | 0 | 0.009 | 0.006 | 0.008 | 0.003 | 0.002 |
| 472.5 | 0 | 0.009 | 0.007 | 0.009 | 0.003 | 0.002 |
| 472 | 0 | 0.009 | 0.007 | 0.009 | 0.003 | 0.003 |
| 471.5 | 0 | 0.009 | 0.007 | 0.009 | 0.003 | 0.003 |
| 471 | 0 | 0.009 | 0.007 | 0.009 | 0.004 | 0.003 |
| 470.5 | 0 | 0.01 | 0.008 | 0.009 | 0.004 | 0.003 |
| 470 | 0 | 0.01 | 0.008 | 0.01 | 0.005 | 0.004 |
| 469.5 | 0 | 0.01 | 0.008 | 0.01 | 0.005 | 0.004 |
| 469 | 0 | 0.011 | 0.009 | 0.01 | 0.005 | 0.004 |
| 468.5 | 0 | 0.011 | 0.009 | 0.011 | 0.006 | 0.005 |
| 468 | 0 | 0.011 | 0.009 | 0.011 | 0.006 | 0.005 |
| 467.5 | 0 | 0.011 | 0.009 | 0.011 | 0.006 | 0.005 |
| 467 | 0 | 0.011 | 0.008 | 0.01 | 0.006 | 0.005 |
| 466.5 | 0 | 0.011 | 0.009 | 0.01 | 0.005 | 0.005 |
| 466 | 0 | 0.011 | 0.009 | 0.011 | 0.006 | 0.005 |
| 465.5 | 0.001 | 0.011 | 0.009 | 0.011 | 0.006 | 0.005 |
| 465 | 0.001 | 0.011 | 0.01 | 0.011 | 0.006 | 0.005 |
| 464.5 | 0.001 | 0.012 | 0.01 | 0.012 | 0.007 | 0.006 |
| 464 | 0.001 | 0.012 | 0.01 | 0.012 | 0.007 | 0.006 |
| 463.5 | 0.001 | 0.012 | 0.011 | 0.012 | 0.007 | 0.006 |
| 463 | 0.001 | 0.013 | 0.011 | 0.013 | 0.008 | 0.006 |
| 462.5 | 0.001 | 0.013 | 0.01 | 0.013 | 0.008 | 0.006 |
| 462 | 0.001 | 0.013 | 0.011 | 0.013 | 0.008 | 0.007 |
| 461.5 | 0.001 | 0.013 | 0.011 | 0.013 | 0.008 | 0.007 |
| 461 | 0.001 | 0.013 | 0.011 | 0.013 | 0.008 | 0.008 |
| 460.5 | 0.001 | 0.014 | 0.012 | 0.014 | 0.009 | 0.008 |
| 460 | 0.001 | 0.014 | 0.012 | 0.014 | 0.009 | 0.008 |
| 459.5 | 0.001 | 0.014 | 0.012 | 0.014 | 0.009 | 0.008 |
| 459 | 0.001 | 0.014 | 0.012 | 0.014 | 0.009 | 0.008 |
| 458.5 | 0.001 | 0.014 | 0.012 | 0.014 | 0.009 | 0.008 |
| 458 | 0.001 | 0.014 | 0.012 | 0.014 | 0.009 | 0.008 |
| 457.5 | 0.001 | 0.014 | 0.013 | 0.014 | 0.01 | 0.009 |
| 457 | 0.002 | 0.015 | 0.013 | 0.015 | 0.01 | 0.009 |
| 456.5 | 0.002 | 0.014 | 0.013 | 0.015 | 0.01 | 0.009 |
| 456 | 0.002 | 0.015 | 0.013 | 0.015 | 0.01 | 0.009 |
| 455.5 | 0.001 | 0.015 | 0.013 | 0.015 | 0.01 | 0.009 |
| 455 | 0.001 | 0.015 | 0.013 | 0.015 | 0.01 | 0.009 |
| 454.5 | 0.002 | 0.015 | 0.014 | 0.015 | 0.011 | 0.01 |
| 454 | 0.002 | 0.016 | 0.014 | 0.016 | 0.01 | 0.01 |
| 453.5 | 0.002 | 0.016 | 0.014 | 0.016 | 0.01 | 0.01 |
| 453 | 0.002 | 0.016 | 0.015 | 0.017 | 0.011 | 0.011 |
| 452.5 | 0.002 | 0.016 | 0.014 | 0.017 | 0.011 | 0.01 |
| 452 | 0.002 | 0.016 | 0.014 | 0.017 | 0.011 | 0.01 |
| 451.5 | 0.002 | 0.016 | 0.014 | 0.017 | 0.012 | 0.011 |
| 451 | 0.002 | 0.016 | 0.015 | 0.017 | 0.012 | 0.011 |
| 450.5 | 0.002 | 0.016 | 0.015 | 0.017 | 0.012 | 0.011 |
| 450 | 0.002 | 0.017 | 0.015 | 0.017 | 0.012 | 0.011 |
| 449.5 | 0.002 | 0.017 | 0.015 | 0.017 | 0.012 | 0.011 |
| 449 | 0.003 | 0.016 | 0.015 | 0.017 | 0.012 | 0.011 |
| 448.5 | 0.003 | 0.017 | 0.015 | 0.017 | 0.012 | 0.011 |
| 448 | 0.003 | 0.017 | 0.015 | 0.017 | 0.012 | 0.011 |
| 447.5 | 0.003 | 0.016 | 0.015 | 0.017 | 0.012 | 0.012 |
| 447 | 0.003 | 0.017 | 0.015 | 0.017 | 0.012 | 0.012 |
| 446.5 | 0.003 | 0.017 | 0.015 | 0.018 | 0.013 | 0.012 |
| 446 | 0.003 | 0.017 | 0.015 | 0.018 | 0.012 | 0.011 |
| 445.5 | 0.003 | 0.017 | 0.015 | 0.018 | 0.013 | 0.012 |
| 445 | 0.003 | 0.017 | 0.015 | 0.018 | 0.013 | 0.012 |
| 444.5 | 0.003 | 0.017 | 0.015 | 0.018 | 0.012 | 0.012 |
| 444 | 0.003 | 0.017 | 0.015 | 0.018 | 0.012 | 0.012 |
| 443.5 | 0.003 | 0.016 | 0.015 | 0.018 | 0.013 | 0.012 |
| 443 | 0.003 | 0.016 | 0.015 | 0.018 | 0.013 | 0.012 |
| 442.5 | 0.003 | 0.017 | 0.015 | 0.018 | 0.013 | 0.012 |
| 442 | 0.004 | 0.017 | 0.016 | 0.018 | 0.013 | 0.012 |
| 441.5 | 0.004 | 0.017 | 0.015 | 0.018 | 0.013 | 0.012 |
| 441 | 0.004 | 0.016 | 0.015 | 0.017 | 0.013 | 0.012 |
| 440.5 | 0.004 | 0.017 | 0.016 | 0.018 | 0.012 | 0.012 |
| 440 | 0.004 | 0.017 | 0.016 | 0.018 | 0.013 | 0.012 |
| 439.5 | 0.004 | 0.017 | 0.016 | 0.018 | 0.013 | 0.013 |
| 439 | 0.004 | 0.016 | 0.016 | 0.019 | 0.013 | 0.012 |
| 438.5 | 0.004 | 0.017 | 0.016 | 0.018 | 0.013 | 0.012 |
| 438 | 0.004 | 0.017 | 0.016 | 0.018 | 0.013 | 0.012 |
| 437.5 | 0.004 | 0.017 | 0.015 | 0.018 | 0.013 | 0.012 |
| 437 | 0.004 | 0.017 | 0.015 | 0.018 | 0.013 | 0.012 |
| 436.5 | 0.004 | 0.016 | 0.015 | 0.018 | 0.013 | 0.012 |
| 436 | 0.004 | 0.017 | 0.015 | 0.018 | 0.013 | 0.012 |
| 435.5 | 0.005 | 0.017 | 0.015 | 0.018 | 0.013 | 0.012 |
| 435 | 0.005 | 0.016 | 0.015 | 0.018 | 0.013 | 0.012 |
| 434.5 | 0.005 | 0.016 | 0.015 | 0.018 | 0.012 | 0.012 |
| 434 | 0.005 | 0.016 | 0.015 | 0.018 | 0.012 | 0.012 |
| 433.5 | 0.005 | 0.016 | 0.015 | 0.018 | 0.013 | 0.012 |
| 433 | 0.005 | 0.016 | 0.015 | 0.018 | 0.013 | 0.012 |
| 432.5 | 0.005 | 0.017 | 0.015 | 0.018 | 0.013 | 0.012 |
| 432 | 0.005 | 0.016 | 0.015 | 0.017 | 0.012 | 0.012 |
| 431.5 | 0.005 | 0.016 | 0.015 | 0.017 | 0.012 | 0.012 |
| 431 | 0.006 | 0.016 | 0.015 | 0.017 | 0.012 | 0.011 |
| 430.5 | 0.006 | 0.015 | 0.014 | 0.017 | 0.012 | 0.011 |
| 430 | 0.005 | 0.015 | 0.014 | 0.017 | 0.012 | 0.011 |
| 429.5 | 0.005 | 0.015 | 0.014 | 0.017 | 0.012 | 0.011 |
| 429 | 0.006 | 0.015 | 0.014 | 0.017 | 0.012 | 0.011 |
| 428.5 | 0.006 | 0.016 | 0.014 | 0.017 | 0.012 | 0.011 |
| 428 | 0.006 | 0.015 | 0.014 | 0.017 | 0.012 | 0.011 |
| 427.5 | 0.006 | 0.015 | 0.014 | 0.017 | 0.012 | 0.011 |
| 427 | 0.006 | 0.015 | 0.014 | 0.016 | 0.011 | 0.011 |
| 426.5 | 0.006 | 0.014 | 0.014 | 0.016 | 0.011 | 0.01 |
| 426 | 0.006 | 0.015 | 0.014 | 0.016 | 0.011 | 0.01 |
| 425.5 | 0.006 | 0.015 | 0.014 | 0.016 | 0.011 | 0.01 |
| 425 | 0.006 | 0.015 | 0.014 | 0.016 | 0.011 | 0.01 |
| 424.5 | 0.006 | 0.015 | 0.013 | 0.016 | 0.011 | 0.01 |
| 424 | 0.006 | 0.014 | 0.013 | 0.016 | 0.011 | 0.01 |
| 423.5 | 0.006 | 0.014 | 0.013 | 0.016 | 0.011 | 0.01 |
| 423 | 0.007 | 0.014 | 0.013 | 0.016 | 0.011 | 0.01 |
| 422.5 | 0.007 | 0.014 | 0.013 | 0.016 | 0.01 | 0.01 |
| 422 | 0.007 | 0.014 | 0.013 | 0.015 | 0.01 | 0.01 |
| 421.5 | 0.007 | 0.014 | 0.013 | 0.015 | 0.01 | 0.01 |
| 421 | 0.007 | 0.014 | 0.013 | 0.015 | 0.01 | 0.01 |
| 420.5 | 0.007 | 0.014 | 0.013 | 0.015 | 0.01 | 0.01 |
| 420 | 0.007 | 0.013 | 0.012 | 0.015 | 0.01 | 0.01 |
| 419.5 | 0.007 | 0.013 | 0.012 | 0.015 | 0.01 | 0.009 |
| 419 | 0.007 | 0.013 | 0.012 | 0.015 | 0.01 | 0.009 |
| 418.5 | 0.007 | 0.013 | 0.012 | 0.015 | 0.01 | 0.009 |
| 418 | 0.007 | 0.013 | 0.012 | 0.015 | 0.01 | 0.009 |
| 417.5 | 0.007 | 0.013 | 0.012 | 0.015 | 0.01 | 0.009 |
| 417 | 0.007 | 0.013 | 0.012 | 0.015 | 0.01 | 0.009 |
| 416.5 | 0.007 | 0.013 | 0.012 | 0.014 | 0.009 | 0.009 |
| 416 | 0.007 | 0.013 | 0.012 | 0.014 | 0.009 | 0.009 |
| 415.5 | 0.007 | 0.012 | 0.011 | 0.014 | 0.009 | 0.009 |
| 415 | 0.008 | 0.012 | 0.011 | 0.014 | 0.009 | 0.008 |
| 414.5 | 0.008 | 0.012 | 0.011 | 0.014 | 0.009 | 0.008 |
| 414 | 0.008 | 0.012 | 0.011 | 0.014 | 0.009 | 0.008 |
| 413.5 | 0.008 | 0.012 | 0.011 | 0.014 | 0.009 | 0.008 |
| 413 | 0.008 | 0.012 | 0.011 | 0.014 | 0.009 | 0.008 |
| 412.5 | 0.008 | 0.012 | 0.011 | 0.014 | 0.009 | 0.008 |
| 412 | 0.008 | 0.012 | 0.011 | 0.014 | 0.009 | 0.008 |
| 411.5 | 0.008 | 0.012 | 0.011 | 0.014 | 0.009 | 0.008 |
| 411 | 0.008 | 0.012 | 0.011 | 0.014 | 0.009 | 0.008 |
| 410.5 | 0.008 | 0.011 | 0.011 | 0.013 | 0.008 | 0.008 |
| 410 | 0.008 | 0.011 | 0.01 | 0.013 | 0.008 | 0.008 |
| 409.5 | 0.008 | 0.011 | 0.01 | 0.013 | 0.008 | 0.008 |
| 409 | 0.008 | 0.011 | 0.01 | 0.013 | 0.008 | 0.008 |
| 408.5 | 0.008 | 0.011 | 0.01 | 0.013 | 0.008 | 0.007 |
| 408 | 0.009 | 0.011 | 0.01 | 0.013 | 0.008 | 0.007 |
| 407.5 | 0.009 | 0.011 | 0.01 | 0.013 | 0.008 | 0.007 |
| 407 | 0.009 | 0.011 | 0.01 | 0.013 | 0.008 | 0.007 |
| 406.5 | 0.009 | 0.011 | 0.01 | 0.013 | 0.008 | 0.007 |
| 406 | 0.009 | 0.011 | 0.01 | 0.013 | 0.008 | 0.007 |
| 405.5 | 0.009 | 0.011 | 0.01 | 0.013 | 0.008 | 0.007 |
| 405 | 0.009 | 0.011 | 0.01 | 0.013 | 0.008 | 0.007 |
| 404.5 | 0.009 | 0.01 | 0.01 | 0.012 | 0.008 | 0.007 |
| 404 | 0.009 | 0.01 | 0.01 | 0.012 | 0.007 | 0.007 |
| 403.5 | 0.009 | 0.01 | 0.01 | 0.012 | 0.007 | 0.007 |
| 403 | 0.009 | 0.01 | 0.009 | 0.012 | 0.007 | 0.007 |
| 402.5 | 0.01 | 0.01 | 0.009 | 0.012 | 0.007 | 0.006 |
| 402 | 0.01 | 0.01 | 0.009 | 0.012 | 0.007 | 0.006 |
| 401.5 | 0.01 | 0.01 | 0.009 | 0.012 | 0.007 | 0.006 |
| 401 | 0.01 | 0.01 | 0.009 | 0.012 | 0.007 | 0.006 |
| 400.5 | 0.01 | 0.01 | 0.009 | 0.012 | 0.007 | 0.006 |
| 400 | 0.01 | 0.01 | 0.009 | 0.012 | 0.007 | 0.006 |
| 399.5 | 0.01 | 0.01 | 0.009 | 0.012 | 0.007 | 0.006 |
| 399 | 0.01 | 0.01 | 0.009 | 0.012 | 0.007 | 0.006 |
| 398.5 | 0.01 | 0.01 | 0.009 | 0.011 | 0.007 | 0.006 |
| 398 | 0.01 | 0.01 | 0.009 | 0.011 | 0.007 | 0.006 |
| 397.5 | 0.01 | 0.01 | 0.009 | 0.011 | 0.006 | 0.006 |
| 397 | 0.01 | 0.009 | 0.009 | 0.011 | 0.006 | 0.006 |
| 396.5 | 0.011 | 0.009 | 0.009 | 0.011 | 0.006 | 0.006 |
| 396 | 0.011 | 0.009 | 0.008 | 0.011 | 0.006 | 0.006 |
| 395.5 | 0.011 | 0.009 | 0.008 | 0.011 | 0.006 | 0.006 |
| 395 | 0.011 | 0.009 | 0.008 | 0.011 | 0.006 | 0.005 |
| 394.5 | 0.011 | 0.009 | 0.008 | 0.011 | 0.006 | 0.005 |
| 394 | 0.011 | 0.009 | 0.008 | 0.011 | 0.006 | 0.006 |
| 393.5 | 0.012 | 0.009 | 0.008 | 0.011 | 0.006 | 0.006 |
| 393 | 0.012 | 0.009 | 0.008 | 0.011 | 0.006 | 0.005 |
| 392.5 | 0.012 | 0.009 | 0.008 | 0.011 | 0.006 | 0.005 |
| 392 | 0.012 | 0.009 | 0.008 | 0.011 | 0.006 | 0.005 |
| 391.5 | 0.012 | 0.009 | 0.008 | 0.01 | 0.006 | 0.005 |
| 391 | 0.012 | 0.009 | 0.008 | 0.01 | 0.006 | 0.005 |
| 390.5 | 0.012 | 0.008 | 0.008 | 0.01 | 0.006 | 0.005 |
| 390 | 0.012 | 0.008 | 0.008 | 0.01 | 0.005 | 0.005 |
| 389.5 | 0.013 | 0.008 | 0.008 | 0.01 | 0.005 | 0.005 |
| 389 | 0.013 | 0.008 | 0.008 | 0.01 | 0.005 | 0.005 |
| 388.5 | 0.013 | 0.008 | 0.008 | 0.01 | 0.005 | 0.005 |
| 388 | 0.013 | 0.008 | 0.007 | 0.01 | 0.005 | 0.005 |
| 387.5 | 0.013 | 0.008 | 0.007 | 0.01 | 0.005 | 0.005 |
| 387 | 0.014 | 0.008 | 0.007 | 0.01 | 0.005 | 0.005 |
| 386.5 | 0.014 | 0.008 | 0.007 | 0.01 | 0.005 | 0.005 |
| 386 | 0.014 | 0.008 | 0.007 | 0.01 | 0.005 | 0.005 |
| 385.5 | 0.014 | 0.008 | 0.007 | 0.01 | 0.005 | 0.004 |
| 385 | 0.014 | 0.008 | 0.007 | 0.01 | 0.005 | 0.005 |
| 384.5 | 0.014 | 0.008 | 0.007 | 0.01 | 0.005 | 0.004 |
| 384 | 0.015 | 0.008 | 0.007 | 0.01 | 0.005 | 0.004 |
| 383.5 | 0.015 | 0.008 | 0.007 | 0.01 | 0.005 | 0.004 |
| 383 | 0.015 | 0.008 | 0.007 | 0.01 | 0.005 | 0.005 |
| 382.5 | 0.015 | 0.008 | 0.007 | 0.01 | 0.005 | 0.004 |
| 382 | 0.015 | 0.008 | 0.007 | 0.01 | 0.005 | 0.004 |
| 381.5 | 0.016 | 0.008 | 0.007 | 0.01 | 0.005 | 0.004 |
| 381 | 0.016 | 0.008 | 0.007 | 0.01 | 0.005 | 0.004 |
| 380.5 | 0.016 | 0.008 | 0.007 | 0.01 | 0.005 | 0.004 |
| 380 | 0.016 | 0.007 | 0.007 | 0.01 | 0.005 | 0.004 |
| 379.5 | 0.017 | 0.007 | 0.007 | 0.01 | 0.005 | 0.004 |
| 379 | 0.017 | 0.007 | 0.007 | 0.009 | 0.005 | 0.004 |
| 378.5 | 0.017 | 0.007 | 0.006 | 0.009 | 0.004 | 0.004 |
| 378 | 0.017 | 0.007 | 0.006 | 0.009 | 0.004 | 0.004 |
| 377.5 | 0.018 | 0.007 | 0.006 | 0.009 | 0.004 | 0.004 |
| 377 | 0.018 | 0.007 | 0.006 | 0.009 | 0.004 | 0.004 |
| 376.5 | 0.018 | 0.007 | 0.006 | 0.009 | 0.004 | 0.004 |
| 376 | 0.018 | 0.007 | 0.006 | 0.009 | 0.004 | 0.004 |
| 375.5 | 0.019 | 0.007 | 0.006 | 0.009 | 0.004 | 0.004 |
| 375 | 0.019 | 0.007 | 0.006 | 0.009 | 0.004 | 0.003 |
| 374.5 | 0.019 | 0.007 | 0.006 | 0.009 | 0.004 | 0.003 |
| 374 | 0.02 | 0.006 | 0.006 | 0.009 | 0.004 | 0.003 |
| 373.5 | 0.02 | 0.006 | 0.006 | 0.009 | 0.004 | 0.003 |
| 373 | 0.02 | 0.006 | 0.006 | 0.008 | 0.004 | 0.003 |
| 372.5 | 0.021 | 0.006 | 0.006 | 0.008 | 0.003 | 0.003 |
| 372 | 0.021 | 0.006 | 0.005 | 0.008 | 0.003 | 0.003 |
| 371.5 | 0.021 | 0.006 | 0.005 | 0.008 | 0.003 | 0.003 |
| 371 | 0.021 | 0.006 | 0.005 | 0.008 | 0.003 | 0.003 |
| 370.5 | 0.021 | 0.006 | 0.005 | 0.008 | 0.003 | 0.003 |
| 370 | 0.022 | 0.006 | 0.005 | 0.008 | 0.003 | 0.003 |
| 369.5 | 0.022 | 0.006 | 0.005 | 0.008 | 0.003 | 0.003 |
| 369 | 0.022 | 0.006 | 0.006 | 0.008 | 0.003 | 0.003 |
| 368.5 | 0.023 | 0.006 | 0.005 | 0.008 | 0.003 | 0.003 |
| 368 | 0.023 | 0.006 | 0.005 | 0.008 | 0.003 | 0.002 |
| 367.5 | 0.023 | 0.005 | 0.005 | 0.008 | 0.003 | 0.002 |
| 367 | 0.024 | 0.005 | 0.005 | 0.007 | 0.003 | 0.002 |
| 366.5 | 0.024 | 0.005 | 0.005 | 0.007 | 0.003 | 0.002 |
| 366 | 0.024 | 0.005 | 0.005 | 0.007 | 0.003 | 0.002 |
| 365.5 | 0.024 | 0.005 | 0.005 | 0.007 | 0.002 | 0.002 |
| 365 | 0.025 | 0.005 | 0.004 | 0.007 | 0.002 | 0.002 |
| 364.5 | 0.025 | 0.005 | 0.004 | 0.007 | 0.002 | 0.002 |
| 364 | 0.025 | 0.005 | 0.004 | 0.007 | 0.002 | 0.002 |
| 363.5 | 0.026 | 0.005 | 0.004 | 0.007 | 0.002 | 0.002 |
| 363 | 0.026 | 0.005 | 0.004 | 0.007 | 0.002 | 0.002 |
| 362.5 | 0.026 | 0.005 | 0.004 | 0.007 | 0.002 | 0.001 |
| 362 | 0.026 | 0.005 | 0.004 | 0.007 | 0.002 | 0.001 |
| 361.5 | 0.027 | 0.005 | 0.004 | 0.006 | 0.002 | 0.001 |
| 361 | 0.027 | 0.004 | 0.004 | 0.006 | 0.002 | 0.001 |
| 360.5 | 0.027 | 0.004 | 0.004 | 0.006 | 0.002 | 0.001 |
| 360 | 0.027 | 0.004 | 0.004 | 0.006 | 0.002 | 0.001 |
| 359.5 | 0.027 | 0.004 | 0.004 | 0.006 | 0.002 | 0.001 |
| 359 | 0.028 | 0.004 | 0.004 | 0.006 | 0.001 | 0.001 |
| 358.5 | 0.028 | 0.004 | 0.003 | 0.006 | 0.001 | 0.001 |
| 358 | 0.029 | 0.004 | 0.003 | 0.006 | 0.001 | 0.001 |
| 357.5 | 0.029 | 0.004 | 0.003 | 0.006 | 0.001 | 0.001 |
| 357 | 0.029 | 0.004 | 0.003 | 0.006 | 0.001 | 0.001 |
| 356.5 | 0.029 | 0.004 | 0.003 | 0.006 | 0.001 | 0.001 |
| 356 | 0.029 | 0.004 | 0.003 | 0.006 | 0.001 | 0.001 |
| 355.5 | 0.03 | 0.004 | 0.003 | 0.006 | 0.001 | 0 |
| 355 | 0.03 | 0.003 | 0.003 | 0.006 | 0.001 | 0 |
| 354.5 | 0.03 | 0.003 | 0.003 | 0.005 | 0.001 | 0 |
| 354 | 0.03 | 0.003 | 0.003 | 0.005 | 0.001 | 0 |
| 353.5 | 0.031 | 0.003 | 0.003 | 0.005 | 0.001 | 0 |
| 353 | 0.031 | 0.003 | 0.003 | 0.005 | 0.001 | 0 |
| 352.5 | 0.031 | 0.003 | 0.003 | 0.005 | 0.001 | 0 |
| 352 | 0.031 | 0.003 | 0.003 | 0.005 | 0 | 0 |
| 351.5 | 0.032 | 0.003 | 0.002 | 0.005 | 0 | 0 |
| 351 | 0.032 | 0.003 | 0.002 | 0.005 | 0 | 0 |
| 350.5 | 0.032 | 0.003 | 0.002 | 0.005 | 0 | 0 |
| 350 | 0.032 | 0.003 | 0.002 | 0.005 | 0 | 0 |
| 349.5 | 0.032 | 0.003 | 0.002 | 0.005 | 0 | 0 |
| 349 | 0.032 | 0.003 | 0.002 | 0.005 | 0 | 0 |
| 348.5 | 0.032 | 0.003 | 0.002 | 0.005 | 0 | -0.001 |
| 348 | 0.033 | 0.002 | 0.002 | 0.005 | 0 | -0.001 |
| 347.5 | 0.033 | 0.002 | 0.002 | 0.005 | 0 | -0.001 |
| 347 | 0.033 | 0.002 | 0.002 | 0.004 | 0 | -0.001 |
| 346.5 | 0.033 | 0.002 | 0.002 | 0.004 | 0 | -0.001 |
| 346 | 0.033 | 0.002 | 0.002 | 0.004 | 0 | -0.001 |
| 345.5 | 0.034 | 0.002 | 0.001 | 0.004 | -0.001 | -0.001 |
| 345 | 0.033 | 0.002 | 0.001 | 0.004 | -0.001 | -0.001 |
| 344.5 | 0.033 | 0.002 | 0.001 | 0.004 | -0.001 | -0.001 |
| 344 | 0.033 | 0.002 | 0.001 | 0.004 | -0.001 | -0.001 |
| 343.5 | 0.034 | 0.002 | 0.001 | 0.004 | -0.001 | -0.001 |
| 343 | 0.034 | 0.002 | 0.001 | 0.004 | -0.001 | -0.001 |
| 342.5 | 0.034 | 0.002 | 0.001 | 0.004 | -0.001 | -0.001 |
| 342 | 0.033 | 0.002 | 0.001 | 0.004 | -0.001 | -0.001 |
| 341.5 | 0.033 | 0.002 | 0.001 | 0.004 | -0.001 | -0.001 |
| 341 | 0.034 | 0.002 | 0.001 | 0.004 | -0.001 | -0.001 |
| 340.5 | 0.034 | 0.002 | 0.001 | 0.004 | -0.001 | -0.001 |
| 340 | 0.034 | 0.002 | 0.001 | 0.004 | -0.001 | -0.001 |
| 339.5 | 0.034 | 0.002 | 0.001 | 0.004 | -0.001 | -0.001 |
| 339 | 0.034 | 0.001 | 0.001 | 0.004 | -0.001 | -0.001 |
| 338.5 | 0.034 | 0.001 | 0.001 | 0.003 | -0.001 | -0.001 |
| 338 | 0.034 | 0.001 | 0.001 | 0.003 | -0.001 | -0.002 |
| 337.5 | 0.034 | 0.001 | 0.001 | 0.003 | -0.001 | -0.002 |
| 337 | 0.033 | 0.001 | 0.001 | 0.003 | -0.001 | -0.002 |
| 336.5 | 0.033 | 0.001 | 0.001 | 0.003 | -0.001 | -0.002 |
| 336 | 0.033 | 0.001 | 0.001 | 0.003 | -0.001 | -0.002 |
| 335.5 | 0.033 | 0.001 | 0.001 | 0.003 | -0.001 | -0.002 |
| 335 | 0.033 | 0.001 | 0.001 | 0.003 | -0.001 | -0.002 |
| 334.5 | 0.033 | 0.001 | 0.001 | 0.003 | -0.001 | -0.002 |
| 334 | 0.033 | 0.001 | 0.001 | 0.003 | -0.002 | -0.002 |
| 333.5 | 0.033 | 0.001 | 0 | 0.003 | -0.002 | -0.002 |
| 333 | 0.033 | 0.001 | 0 | 0.003 | -0.002 | -0.002 |
| 332.5 | 0.034 | 0.001 | 0 | 0.003 | -0.002 | -0.002 |
| 332 | 0.033 | 0.001 | 0 | 0.003 | -0.002 | -0.002 |
| 331.5 | 0.033 | 0 | 0 | 0.003 | -0.002 | -0.002 |
| 331 | 0.033 | 0 | 0 | 0.002 | -0.002 | -0.002 |
| 330.5 | 0.033 | 0 | 0 | 0.002 | -0.002 | -0.002 |
| 330 | 0.033 | 0 | 0 | 0.002 | -0.002 | -0.002 |
| 329.5 | 0.033 | 0 | 0 | 0.002 | -0.002 | -0.002 |
| 329 | 0.033 | 0 | 0 | 0.002 | -0.002 | -0.003 |
| 328.5 | 0.033 | 0 | 0 | 0.002 | -0.002 | -0.003 |
| 328 | 0.033 | 0 | 0 | 0.002 | -0.002 | -0.003 |
| 327.5 | 0.033 | 0 | 0 | 0.002 | -0.002 | -0.003 |
| 327 | 0.033 | 0 | 0 | 0.002 | -0.002 | -0.003 |
| 326.5 | 0.032 | 0 | 0 | 0.002 | -0.002 | -0.003 |
| 326 | 0.032 | 0 | 0 | 0.002 | -0.003 | -0.003 |
| 325.5 | 0.032 | 0 | -0.001 | 0.002 | -0.003 | -0.003 |
| 325 | 0.031 | 0 | -0.001 | 0.002 | -0.003 | -0.003 |
| 324.5 | 0.031 | 0 | -0.001 | 0.002 | -0.003 | -0.003 |
| 324 | 0.031 | -0.001 | -0.001 | 0.001 | -0.003 | -0.003 |
| 323.5 | 0.03 | -0.001 | -0.001 | 0.001 | -0.003 | -0.003 |
| 323 | 0.031 | -0.001 | -0.001 | 0.001 | -0.003 | -0.004 |
| 322.5 | 0.03 | -0.001 | -0.001 | 0.001 | -0.003 | -0.004 |
| 322 | 0.03 | -0.001 | -0.001 | 0.001 | -0.003 | -0.004 |
| 321.5 | 0.03 | -0.001 | -0.001 | 0.001 | -0.003 | -0.004 |
| 321 | 0.03 | -0.001 | -0.001 | 0.001 | -0.003 | -0.004 |
| 320.5 | 0.029 | -0.001 | -0.001 | 0.001 | -0.003 | -0.004 |
| 320 | 0.029 | -0.001 | -0.001 | 0.001 | -0.004 | -0.004 |
| 319.5 | 0.029 | -0.001 | -0.002 | 0.001 | -0.004 | -0.004 |
| 319 | 0.028 | -0.001 | -0.002 | 0 | -0.004 | -0.004 |
| 318.5 | 0.028 | -0.001 | -0.002 | 0 | -0.004 | -0.004 |
| 318 | 0.028 | -0.001 | -0.002 | 0 | -0.004 | -0.004 |
| 317.5 | 0.028 | -0.002 | -0.002 | 0 | -0.004 | -0.004 |
| 317 | 0.028 | -0.002 | -0.002 | 0 | -0.004 | -0.004 |
| 316.5 | 0.028 | -0.002 | -0.002 | 0 | -0.004 | -0.005 |
| 316 | 0.027 | -0.002 | -0.002 | 0 | -0.004 | -0.005 |
| 315.5 | 0.027 | -0.002 | -0.002 | 0 | -0.004 | -0.005 |
| 315 | 0.027 | -0.002 | -0.002 | 0 | -0.004 | -0.005 |
| 314.5 | 0.027 | -0.002 | -0.002 | 0 | -0.004 | -0.005 |
| 314 | 0.026 | -0.002 | -0.002 | 0 | -0.004 | -0.005 |
| 313.5 | 0.026 | -0.002 | -0.002 | 0 | -0.004 | -0.005 |
| 313 | 0.026 | -0.002 | -0.002 | 0 | -0.004 | -0.005 |
| 312.5 | 0.026 | -0.002 | -0.002 | 0 | -0.005 | -0.005 |
| 312 | 0.026 | -0.002 | -0.003 | -0.001 | -0.005 | -0.005 |
| 311.5 | 0.026 | -0.002 | -0.003 | -0.001 | -0.005 | -0.005 |
| 311 | 0.026 | -0.002 | -0.003 | -0.001 | -0.005 | -0.005 |
| 310.5 | 0.026 | -0.002 | -0.003 | -0.001 | -0.005 | -0.005 |
| 310 | 0.025 | -0.003 | -0.003 | -0.001 | -0.005 | -0.005 |
| 309.5 | 0.024 | -0.003 | -0.003 | -0.001 | -0.005 | -0.005 |
| 309 | 0.024 | -0.003 | -0.003 | -0.001 | -0.005 | -0.005 |
| 308.5 | 0.024 | -0.003 | -0.003 | -0.001 | -0.005 | -0.005 |
| 308 | 0.024 | -0.003 | -0.003 | -0.001 | -0.005 | -0.006 |
| 307.5 | 0.024 | -0.003 | -0.003 | -0.001 | -0.005 | -0.006 |
| 307 | 0.023 | -0.003 | -0.003 | -0.001 | -0.005 | -0.006 |
| 306.5 | 0.023 | -0.003 | -0.003 | -0.001 | -0.005 | -0.006 |
| 306 | 0.023 | -0.003 | -0.004 | -0.001 | -0.005 | -0.006 |
| 305.5 | 0.023 | -0.003 | -0.004 | -0.002 | -0.005 | -0.006 |
| 305 | 0.022 | -0.003 | -0.004 | -0.002 | -0.005 | -0.006 |
| 304.5 | 0.022 | -0.003 | -0.004 | -0.002 | -0.006 | -0.006 |
| 304 | 0.022 | -0.004 | -0.004 | -0.002 | -0.006 | -0.006 |
| 303.5 | 0.021 | -0.004 | -0.004 | -0.002 | -0.006 | -0.006 |
| 303 | 0.021 | -0.004 | -0.004 | -0.002 | -0.006 | -0.006 |
| 302.5 | 0.02 | -0.004 | -0.004 | -0.002 | -0.006 | -0.006 |
| 302 | 0.02 | -0.004 | -0.004 | -0.002 | -0.006 | -0.007 |
| 301.5 | 0.02 | -0.004 | -0.004 | -0.002 | -0.006 | -0.007 |
| 301 | 0.019 | -0.004 | -0.004 | -0.002 | -0.006 | -0.007 |
| 300.5 | 0.019 | -0.004 | -0.004 | -0.003 | -0.006 | -0.007 |
| 300 | 0.019 | -0.004 | -0.005 | -0.003 | -0.006 | -0.007 |
| 299.5 | 0.018 | -0.004 | -0.005 | -0.003 | -0.007 | -0.007 |
| 299 | 0.018 | -0.004 | -0.005 | -0.003 | -0.007 | -0.007 |
| 298.5 | 0.017 | -0.004 | -0.005 | -0.003 | -0.007 | -0.007 |
| 298 | 0.016 | -0.005 | -0.005 | -0.003 | -0.007 | -0.007 |
| 297.5 | 0.016 | -0.005 | -0.005 | -0.003 | -0.007 | -0.007 |
| 297 | 0.016 | -0.005 | -0.005 | -0.003 | -0.007 | -0.007 |
| 296.5 | 0.015 | -0.005 | -0.005 | -0.003 | -0.007 | -0.008 |
| 296 | 0.014 | -0.005 | -0.005 | -0.003 | -0.007 | -0.008 |
| 295.5 | 0.014 | -0.005 | -0.005 | -0.003 | -0.007 | -0.008 |
| 295 | 0.013 | -0.005 | -0.005 | -0.004 | -0.007 | -0.008 |
| 294.5 | 0.012 | -0.005 | -0.005 | -0.004 | -0.007 | -0.008 |
| 294 | 0.012 | -0.005 | -0.005 | -0.004 | -0.007 | -0.008 |
| 293.5 | 0.011 | -0.005 | -0.006 | -0.004 | -0.008 | -0.008 |
| 293 | 0.011 | -0.005 | -0.006 | -0.004 | -0.008 | -0.008 |
| 292.5 | 0.01 | -0.005 | -0.006 | -0.004 | -0.008 | -0.008 |
| 292 | 0.009 | -0.005 | -0.006 | -0.004 | -0.008 | -0.008 |
| 291.5 | 0.008 | -0.005 | -0.006 | -0.004 | -0.008 | -0.008 |
| 291 | 0.008 | -0.005 | -0.006 | -0.004 | -0.008 | -0.008 |
| 290.5 | 0.008 | -0.006 | -0.006 | -0.004 | -0.008 | -0.008 |
| 290 | 0.007 | -0.006 | -0.006 | -0.005 | -0.008 | -0.009 |
| 289.5 | 0.007 | -0.006 | -0.006 | -0.005 | -0.008 | -0.009 |
| 289 | 0.007 | -0.006 | -0.006 | -0.005 | -0.008 | -0.009 |
| 288.5 | 0.007 | -0.006 | -0.006 | -0.005 | -0.008 | -0.009 |
| 288 | 0.007 | -0.006 | -0.006 | -0.005 | -0.008 | -0.009 |
| 287.5 | 0.007 | -0.006 | -0.006 | -0.005 | -0.008 | -0.009 |
| 287 | 0.008 | -0.006 | -0.007 | -0.005 | -0.008 | -0.009 |
| 286.5 | 0.009 | -0.006 | -0.007 | -0.005 | -0.009 | -0.009 |
| 286 | 0.01 | -0.006 | -0.007 | -0.005 | -0.009 | -0.009 |
| 285.5 | 0.01 | -0.006 | -0.007 | -0.005 | -0.009 | -0.009 |
| 285 | 0.011 | -0.007 | -0.007 | -0.005 | -0.009 | -0.009 |
| 284.5 | 0.011 | -0.007 | -0.007 | -0.005 | -0.009 | -0.009 |
| 284 | 0.01 | -0.007 | -0.007 | -0.005 | -0.009 | -0.01 |
| 283.5 | 0.01 | -0.007 | -0.007 | -0.006 | -0.009 | -0.01 |
| 283 | 0.009 | -0.007 | -0.007 | -0.006 | -0.009 | -0.01 |
| 282.5 | 0.007 | -0.007 | -0.007 | -0.006 | -0.009 | -0.01 |
| 282 | 0.005 | -0.007 | -0.007 | -0.006 | -0.009 | -0.01 |
| 281.5 | 0.003 | -0.007 | -0.007 | -0.006 | -0.01 | -0.01 |
| 281 | 0.001 | -0.007 | -0.008 | -0.006 | -0.01 | -0.01 |
| 280.5 | -0.001 | -0.007 | -0.008 | -0.006 | -0.01 | -0.01 |
| 280 | -0.002 | -0.007 | -0.008 | -0.006 | -0.01 | -0.01 |
| 279.5 | -0.004 | -0.007 | -0.008 | -0.006 | -0.01 | -0.01 |
| 279 | -0.005 | -0.007 | -0.008 | -0.006 | -0.01 | -0.01 |
| 278.5 | -0.006 | -0.007 | -0.008 | -0.007 | -0.01 | -0.01 |
| 278 | -0.006 | -0.007 | -0.008 | -0.007 | -0.01 | -0.01 |
| 277.5 | -0.007 | -0.007 | -0.008 | -0.007 | -0.01 | -0.01 |
| 277 | -0.007 | -0.008 | -0.008 | -0.007 | -0.01 | -0.01 |
| 276.5 | -0.007 | -0.008 | -0.008 | -0.007 | -0.01 | -0.01 |
| 276 | -0.007 | -0.008 | -0.008 | -0.007 | -0.01 | -0.011 |
| 275.5 | -0.007 | -0.008 | -0.008 | -0.007 | -0.01 | -0.011 |
| 275 | -0.007 | -0.008 | -0.008 | -0.007 | -0.01 | -0.011 |
| 274.5 | -0.007 | -0.008 | -0.008 | -0.007 | -0.01 | -0.011 |
| 274 | -0.007 | -0.008 | -0.008 | -0.007 | -0.01 | -0.011 |
| 273.5 | -0.007 | -0.008 | -0.009 | -0.007 | -0.01 | -0.011 |
| 273 | -0.008 | -0.008 | -0.009 | -0.007 | -0.01 | -0.011 |
| 272.5 | -0.009 | -0.008 | -0.009 | -0.008 | -0.011 | -0.011 |
| 272 | -0.01 | -0.008 | -0.009 | -0.008 | -0.011 | -0.011 |
| 271.5 | -0.012 | -0.008 | -0.009 | -0.008 | -0.011 | -0.011 |
| 271 | -0.014 | -0.008 | -0.009 | -0.008 | -0.011 | -0.011 |
| 270.5 | -0.016 | -0.008 | -0.009 | -0.008 | -0.011 | -0.011 |
| 270 | -0.019 | -0.009 | -0.009 | -0.008 | -0.011 | -0.012 |
| 269.5 | -0.021 | -0.009 | -0.009 | -0.008 | -0.011 | -0.012 |
| 269 | -0.023 | -0.009 | -0.009 | -0.008 | -0.011 | -0.012 |
| 268.5 | -0.024 | -0.009 | -0.009 | -0.008 | -0.011 | -0.012 |
| 268 | -0.026 | -0.009 | -0.009 | -0.008 | -0.011 | -0.012 |
| 267.5 | -0.027 | -0.009 | -0.009 | -0.008 | -0.011 | -0.012 |
| 267 | -0.029 | -0.009 | -0.009 | -0.008 | -0.011 | -0.012 |
| 266.5 | -0.03 | -0.009 | -0.01 | -0.008 | -0.012 | -0.012 |
| 266 | -0.032 | -0.009 | -0.01 | -0.009 | -0.012 | -0.012 |
| 265.5 | -0.034 | -0.009 | -0.01 | -0.009 | -0.012 | -0.012 |
| 265 | -0.037 | -0.009 | -0.01 | -0.009 | -0.012 | -0.012 |
| 264.5 | -0.039 | -0.009 | -0.01 | -0.009 | -0.012 | -0.012 |
| 264 | -0.042 | -0.009 | -0.01 | -0.009 | -0.012 | -0.012 |
| 263.5 | -0.045 | -0.009 | -0.01 | -0.009 | -0.012 | -0.013 |
| 263 | -0.048 | -0.01 | -0.01 | -0.009 | -0.012 | -0.013 |
| 262.5 | -0.052 | -0.01 | -0.01 | -0.009 | -0.012 | -0.013 |
| 262 | -0.055 | -0.01 | -0.01 | -0.01 | -0.012 | -0.013 |
| 261.5 | -0.057 | -0.01 | -0.01 | -0.01 | -0.012 | -0.013 |
| 261 | -0.06 | -0.01 | -0.01 | -0.01 | -0.013 | -0.013 |
| 260.5 | -0.063 | -0.01 | -0.01 | -0.009 | -0.012 | -0.013 |
| 260 | -0.067 | -0.009 | -0.01 | -0.009 | -0.012 | -0.012 |
| 259.5 | -0.07 | -0.01 | -0.01 | -0.01 | -0.012 | -0.013 |
| 259 | -0.073 | -0.01 | -0.01 | -0.01 | -0.013 | -0.013 |
| 258.5 | -0.076 | -0.01 | -0.011 | -0.01 | -0.013 | -0.013 |
| 258 | -0.079 | -0.01 | -0.011 | -0.01 | -0.013 | -0.014 |
| 257.5 | -0.082 | -0.01 | -0.011 | -0.01 | -0.013 | -0.014 |
| 257 | -0.085 | -0.01 | -0.011 | -0.01 | -0.013 | -0.014 |
| 256.5 | -0.089 | -0.011 | -0.011 | -0.011 | -0.013 | -0.014 |
| 256 | -0.092 | -0.011 | -0.011 | -0.011 | -0.013 | -0.014 |
| 255.5 | -0.094 | -0.011 | -0.011 | -0.011 | -0.014 | -0.014 |
| 255 | -0.098 | -0.011 | -0.012 | -0.011 | -0.014 | -0.014 |
| 254.5 | -0.101 | -0.011 | -0.012 | -0.011 | -0.014 | -0.015 |
| 254 | -0.104 | -0.011 | -0.012 | -0.011 | -0.014 | -0.015 |
| 253.5 | -0.107 | -0.011 | -0.012 | -0.011 | -0.014 | -0.015 |
| 253 | -0.111 | -0.011 | -0.012 | -0.011 | -0.014 | -0.015 |
| 252.5 | -0.115 | -0.012 | -0.012 | -0.012 | -0.014 | -0.015 |
| 252 | -0.119 | -0.012 | -0.012 | -0.012 | -0.015 | -0.015 |
| 251.5 | -0.124 | -0.012 | -0.012 | -0.012 | -0.015 | -0.015 |
| 251 | -0.129 | -0.012 | -0.013 | -0.012 | -0.015 | -0.015 |
| 250.5 | -0.134 | -0.012 | -0.013 | -0.012 | -0.015 | -0.016 |
| 250 | -0.14 | -0.012 | -0.013 | -0.012 | -0.015 | -0.016 |
| 249.5 | -0.146 | -0.013 | -0.013 | -0.013 | -0.015 | -0.016 |
| 249 | -0.152 | -0.013 | -0.013 | -0.013 | -0.015 | -0.016 |
| 248.5 | -0.159 | -0.013 | -0.013 | -0.013 | -0.016 | -0.016 |
| 248 | -0.167 | -0.013 | -0.013 | -0.013 | -0.016 | -0.016 |
| 247.5 | -0.175 | -0.013 | -0.014 | -0.013 | -0.016 | -0.017 |
| 247 | -0.183 | -0.013 | -0.014 | -0.013 | -0.016 | -0.017 |
| 246.5 | -0.193 | -0.013 | -0.014 | -0.013 | -0.016 | -0.017 |
| 246 | -0.203 | -0.014 | -0.014 | -0.014 | -0.016 | -0.017 |
| 245.5 | -0.213 | -0.014 | -0.014 | -0.014 | -0.017 | -0.017 |
| 245 | -0.225 | -0.014 | -0.015 | -0.014 | -0.017 | -0.017 |
| 244.5 | -0.237 | -0.014 | -0.015 | -0.014 | -0.017 | -0.018 |
| 244 | -0.25 | -0.014 | -0.015 | -0.015 | -0.017 | -0.018 |
| 243.5 | -0.263 | -0.015 | -0.015 | -0.015 | -0.017 | -0.018 |
| 243 | -0.277 | -0.015 | -0.015 | -0.015 | -0.018 | -0.018 |
| 242.5 | -0.291 | -0.015 | -0.015 | -0.015 | -0.018 | -0.018 |
| 242 | -0.307 | -0.015 | -0.016 | -0.015 | -0.018 | -0.019 |
| 241.5 | -0.324 | -0.015 | -0.016 | -0.015 | -0.018 | -0.019 |
| 241 | -0.343 | -0.015 | -0.016 | -0.016 | -0.018 | -0.019 |
| 240.5 | -0.361 | -0.015 | -0.016 | -0.016 | -0.018 | -0.019 |
| 240 | -0.379 | -0.016 | -0.016 | -0.016 | -0.019 | -0.019 |
| 239.5 | -0.398 | -0.016 | -0.016 | -0.016 | -0.019 | -0.02 |
| 239 | -0.418 | -0.016 | -0.017 | -0.016 | -0.019 | -0.02 |
| 238.5 | -0.436 | -0.016 | -0.017 | -0.016 | -0.019 | -0.02 |
| 238 | -0.443 | -0.016 | -0.017 | -0.017 | -0.019 | -0.02 |
| 237.5 | -0.442 | -0.017 | -0.017 | -0.017 | -0.02 | -0.02 |
| 237 | -0.441 | -0.017 | -0.017 | -0.017 | -0.02 | -0.021 |
| 236.5 | -0.44 | -0.017 | -0.017 | -0.017 | -0.02 | -0.021 |
| 236 | -0.439 | -0.017 | -0.017 | -0.017 | -0.02 | -0.021 |
| 235.5 | -0.437 | -0.017 | -0.018 | -0.017 | -0.02 | -0.021 |
| 235 | -0.436 | -0.017 | -0.018 | -0.018 | -0.02 | -0.021 |
| 234.5 | -0.435 | -0.017 | -0.018 | -0.018 | -0.02 | -0.021 |
| 234 | -0.434 | -0.018 | -0.018 | -0.018 | -0.021 | -0.021 |
| 233.5 | -0.433 | -0.018 | -0.018 | -0.018 | -0.021 | -0.022 |
| 233 | -0.432 | -0.018 | -0.018 | -0.018 | -0.021 | -0.022 |
| 232.5 | -0.43 | -0.018 | -0.019 | -0.018 | -0.021 | -0.022 |
| 232 | -0.429 | -0.018 | -0.019 | -0.019 | -0.021 | -0.022 |
| 231.5 | -0.428 | -0.018 | -0.019 | -0.019 | -0.021 | -0.022 |
| 231 | -0.427 | -0.019 | -0.019 | -0.019 | -0.022 | -0.022 |
| 230.5 | -0.426 | -0.019 | -0.019 | -0.019 | -0.022 | -0.023 |
| 230 | -0.425 | -0.019 | -0.019 | -0.019 | -0.022 | -0.023 |
| 229.5 | -0.424 | -0.019 | -0.02 | -0.019 | -0.022 | -0.023 |
| 229 | -0.422 | -0.019 | -0.02 | -0.02 | -0.022 | -0.023 |
| 228.5 | -0.421 | -0.019 | -0.02 | -0.02 | -0.022 | -0.023 |
| 228 | -0.42 | -0.019 | -0.02 | -0.02 | -0.022 | -0.023 |
| 227.5 | -0.418 | -0.02 | -0.02 | -0.02 | -0.023 | -0.023 |
| 227 | -0.417 | -0.02 | -0.02 | -0.02 | -0.023 | -0.023 |
| 226.5 | -0.416 | -0.02 | -0.02 | -0.02 | -0.023 | -0.024 |
| 226 | -0.415 | -0.02 | -0.02 | -0.02 | -0.023 | -0.024 |
| 225.5 | -0.414 | -0.02 | -0.021 | -0.021 | -0.023 | -0.024 |
| 225 | -0.412 | -0.02 | -0.021 | -0.021 | -0.023 | -0.024 |
| 224.5 | -0.411 | -0.02 | -0.021 | -0.021 | -0.023 | -0.024 |
| 224 | -0.409 | -0.021 | -0.021 | -0.021 | -0.024 | -0.025 |
| 223.5 | -0.408 | -0.021 | -0.021 | -0.021 | -0.024 | -0.025 |
| 223 | -0.406 | -0.021 | -0.021 | -0.021 | -0.024 | -0.025 |
| 222.5 | -0.405 | -0.021 | -0.021 | -0.021 | -0.024 | -0.025 |
| 222 | -0.404 | -0.021 | -0.022 | -0.022 | -0.024 | -0.025 |
| 221.5 | -0.402 | -0.021 | -0.022 | -0.022 | -0.024 | -0.025 |
| 221 | -0.401 | -0.021 | -0.022 | -0.022 | -0.025 | -0.025 |
| 220.5 | -0.399 | -0.021 | -0.022 | -0.022 | -0.025 | -0.025 |
| 220 | -0.398 | -0.022 | -0.022 | -0.022 | -0.025 | -0.026 |
| 219.5 | -0.396 | -0.022 | -0.022 | -0.022 | -0.025 | -0.026 |
| 219 | -0.395 | -0.022 | -0.022 | -0.022 | -0.025 | -0.026 |
| 218.5 | -0.393 | -0.022 | -0.022 | -0.022 | -0.025 | -0.026 |
| 218 | -0.391 | -0.022 | -0.022 | -0.022 | -0.025 | -0.026 |
| 217.5 | -0.389 | -0.022 | -0.022 | -0.023 | -0.025 | -0.026 |
| 217 | -0.386 | -0.022 | -0.023 | -0.023 | -0.025 | -0.026 |
| 216.5 | -0.384 | -0.022 | -0.023 | -0.023 | -0.025 | -0.026 |
| 216 | -0.382 | -0.022 | -0.023 | -0.023 | -0.025 | -0.026 |
| 215.5 | -0.38 | -0.022 | -0.023 | -0.023 | -0.025 | -0.026 |
| 215 | -0.378 | -0.022 | -0.023 | -0.023 | -0.026 | -0.026 |
| 214.5 | -0.376 | -0.022 | -0.023 | -0.023 | -0.026 | -0.026 |
| 214 | -0.374 | -0.022 | -0.023 | -0.023 | -0.026 | -0.027 |
| 213.5 | -0.371 | -0.023 | -0.023 | -0.023 | -0.026 | -0.027 |
| 213 | -0.369 | -0.023 | -0.023 | -0.023 | -0.026 | -0.027 |
| 212.5 | -0.366 | -0.023 | -0.023 | -0.023 | -0.026 | -0.027 |
| 212 | -0.363 | -0.023 | -0.023 | -0.023 | -0.026 | -0.027 |
| 211.5 | -0.36 | -0.023 | -0.023 | -0.023 | -0.026 | -0.027 |
| 211 | -0.357 | -0.023 | -0.023 | -0.024 | -0.026 | -0.027 |
| 210.5 | -0.354 | -0.023 | -0.024 | -0.024 | -0.026 | -0.027 |
| 210 | -0.351 | -0.023 | -0.024 | -0.024 | -0.026 | -0.027 |
| 209.5 | -0.349 | -0.023 | -0.024 | -0.024 | -0.026 | -0.027 |
| 209 | -0.345 | -0.023 | -0.024 | -0.024 | -0.026 | -0.027 |
| 208.5 | -0.342 | -0.023 | -0.024 | -0.024 | -0.026 | -0.027 |
| 208 | -0.339 | -0.023 | -0.024 | -0.024 | -0.026 | -0.027 |
| 207.5 | -0.335 | -0.023 | -0.024 | -0.024 | -0.027 | -0.027 |
| 207 | -0.33 | -0.023 | -0.024 | -0.024 | -0.027 | -0.028 |
| 206.5 | -0.326 | -0.023 | -0.024 | -0.024 | -0.027 | -0.028 |
| 206 | -0.323 | -0.023 | -0.024 | -0.024 | -0.027 | -0.028 |
| 205.5 | -0.32 | -0.024 | -0.024 | -0.024 | -0.027 | -0.028 |
| 205 | -0.316 | -0.024 | -0.024 | -0.024 | -0.027 | -0.028 |
| 204.5 | -0.312 | -0.024 | -0.024 | -0.025 | -0.027 | -0.028 |
| 204 | -0.308 | -0.024 | -0.024 | -0.025 | -0.027 | -0.028 |
| 203.5 | -0.304 | -0.024 | -0.025 | -0.025 | -0.027 | -0.028 |
| 203 | -0.299 | -0.024 | -0.025 | -0.025 | -0.027 | -0.028 |
| 202.5 | -0.294 | -0.024 | -0.025 | -0.025 | -0.027 | -0.028 |
| 202 | -0.289 | -0.024 | -0.025 | -0.025 | -0.027 | -0.028 |
| 201.5 | -0.285 | -0.024 | -0.025 | -0.025 | -0.027 | -0.028 |
| 201 | -0.282 | -0.024 | -0.025 | -0.025 | -0.028 | -0.029 |
| 200.5 | -0.278 | -0.024 | -0.025 | -0.025 | -0.028 | -0.029 |
| 200 | -0.276 | -0.025 | -0.025 | -0.025 | -0.028 | -0.029 |
